# The Molecular Basis for Ubiquitin and Ubiquitin-like Specificities in Bacterial Effector Proteases

**DOI:** 10.1016/j.molcel.2016.06.015

**Published:** 2016-07-21

**Authors:** Jonathan N. Pruneda, Charlotte H. Durkin, Paul P. Geurink, Huib Ovaa, Balaji Santhanam, David W. Holden, David Komander

**Affiliations:** 1 Division of Protein and Nucleic Acid Chemistry, MRC Laboratory of Molecular Biology, Francis Crick Avenue, Cambridge CB2 0QH, UK; 2 Section of Microbiology, MRC Centre for Molecular Bacteriology and Infection, Imperial College London, London SW7 2AZ, UK; 3 Division of Cell Biology, Netherlands Cancer Institute, Plesmanlaan 121, 1066 Amsterdam, the Netherlands; 4 Division of Structural Studies, MRC Laboratory of Molecular Biology, Francis Crick Avenue, Cambridge CB2 0QH, UK

## Abstract

Pathogenic bacteria rely on secreted effector proteins to manipulate host signaling pathways, often in creative ways. CE clan proteases, specific hydrolases for ubiquitin-like modifications (SUMO and NEDD8) in eukaryotes, reportedly serve as bacterial effector proteins with deSUMOylase, deubiquitinase, or, even, acetyltransferase activities. Here, we characterize bacterial CE protease activities, revealing K63-linkage-specific deubiquitinases in human pathogens, such as *Salmonella*, *Escherichia*, and *Shigella*, as well as ubiquitin/ubiquitin-like cross-reactive enzymes in *Chlamydia*, *Rickettsia*, and *Xanthomonas.* Five crystal structures, including ubiquitin/ubiquitin-like complexes, explain substrate specificities and redefine relationships across the CE clan. Importantly, this work identifies novel family members and provides key discoveries among previously reported effectors, such as the unexpected deubiquitinase activity in *Xanthomonas* XopD, contributed by an unstructured ubiquitin binding region. Furthermore, accessory domains regulate properties such as subcellular localization, as exemplified by a ubiquitin-binding domain in *Salmonella* Typhimurium SseL. Our work both highlights and explains the functional adaptations observed among diverse CE clan proteins.

## Highlights

• Bacterial CE proteases exhibit distinct ubiquitin/ubiquitin-like specificities
• Substrate specificity is acquired through variability in three common regions
• Structural and functional data redefine CE clan relationships across kingdoms
• CE effectors are fitted with accessory domains that modulate function

## Graphical Abstract

**Figure:**
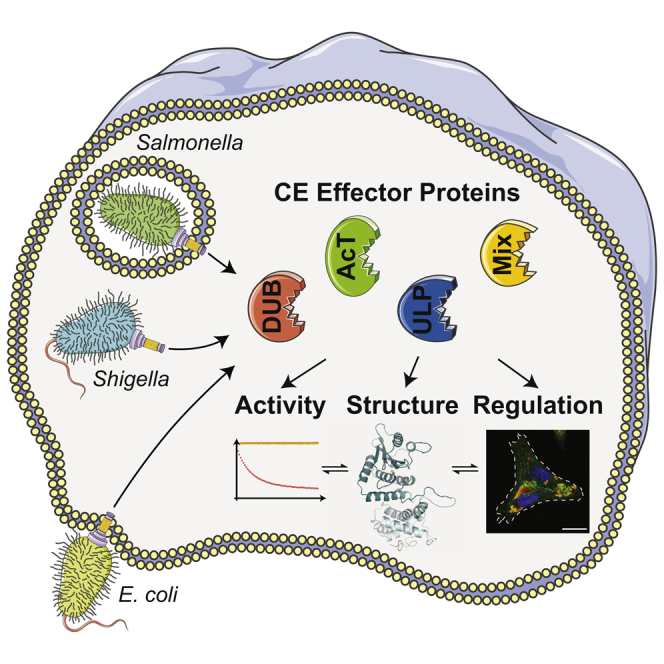

## Teaser

Focusing on examples from pathogenic bacteria, Pruneda et al. examine a family of proteases that displays remarkably distinct specificities toward ubiquitin and ubiquitin-like modifications. Leveraging structural and functional data, the authors derive mechanisms through which substrate specificity is achieved and redefine relationships within the enzyme family across kingdoms of life.

## Introduction

Bacterial colonization and proliferation within a eukaryotic host depends on processes that antagonize immune responses, ensure bacterial survival, and promote replication. For this, bacteria employ a repertoire of effector proteins, ranging in number from dozens to hundreds, which are directly delivered into the host cell by sophisticated secretion machineries (Figueira and Holden, 2012). Once inside, these effector proteins can hijack host factors and catalyze chemical modifications that are, in some cases, entirely foreign to the eukaryotic system (Salomon and Orth, 2013).

Bacterial effectors often target kinase cascades involved in inflammation (Salomon and Orth, 2013) or the eukaryotic ubiquitin (Ub) system (Corn and Vucic, 2014) to promote bacterial pathogenicity. Ub regulates countless cellular processes by forming structurally and functionally distinct polymeric chains that can be used independently or in concert during complex signaling cascades (Swatek and Komander, 2016). In addition, ubiquitin-like (Ubl) proteins, such as NEDD8, SUMO, and ISG15, play similarly important roles in host cell biology (van der Veen and Ploegh, 2012) and invasion response pathways (Liu et al., 2013, Radoshevich et al., 2015).

Ub and Ubl modifications are tightly regulated by discrete families of proteases. Humans encode ∼80 active deubiquitinases (DUBs) that are unable to hydrolyze SUMO or NEDD8, although there are a few exceptions (Clague et al., 2013). The CE protease clan in humans consists of six SUMO-specific SENPs and the NEDD8-specific NEDP1/SENP8 that are collectively termed Ubl proteases (ULPs) (Ronau et al., 2016). Mechanisms dictating the exquisite specificities of these SUMO and NEDD8 proteases have been characterized in detail (Reverter and Lima, 2004, Reverter et al., 2005, Shen et al., 2005).

Bacteria and viruses also encode CE clan enzymes as effectors to interrupt eukaryotic host response processes (Ronau et al., 2016). Importantly, the characterized members from bacteria display diverse enzymatic activities and include not only ULPs (Kim et al., 2008, Orth et al., 2000) but also DUBs (Rytkönen et al., 2007, Misaghi et al., 2006, Zhou et al., 2005, Ye et al., 2007). In addition, some CE effectors, such as *Yersinia pestis* YopJ, display an unusual Ser/Thr acetyltansferase activity (Mittal et al., 2006, Mukherjee et al., 2006, Jones et al., 2008). Hence, it appears that the CE fold is highly versatile and adaptable and has evolved in bacterial pathogens to accommodate a range of enzymatic activities. However, the proteolytic activities and level of cross-reactivity in CE clan effectors are currently unclear since comprehensive comparisons have not been performed, and the molecular basis for achieving distinct target specificities from a single enzyme fold is unknown. Published structures of individual bacterial (Chosed et al., 2007, Sheedlo et al., 2015) and viral examples (Ding et al., 1996) show a similarity to eukaryotic ULPs, but fall short of providing generalizable themes to explain diverse substrate specificities. The uncertainties regarding activity within this diverse family of enzymes have hindered a detailed understanding of the potential role(s) carried out during bacterial invasion.

To address this shortcoming, we cloned and expressed a panel of bacterial CE enzymes for in-depth biochemical and structural analysis. We identify several Ub-specific CE proteases, including the first DUBs in *Shigella* (ShiCE) and *Rickettsia* (RickCE), and show that most prefer K63-linked chains. Ub and K63 specificity is explained by a set of crystal structures, which show immense diversity in their convergent adaptations of a Ub recognition site. By comparing these bacterial examples with their Ubl-specific counterparts in eukaryotes, we identify and define three regions of variability within the CE fold that can be tailored to suit Ub or Ubl specificity. With this knowledge we revisit the *Xanthomonas* effector protein XopD, a reported deSUMOylase in plants, to identify an extended construct that targets not only SUMO but, surprisingly, also Ub modifications. Structures of Ub- and SUMO-bound XopD reveal striking plasticity in substrate recognition, due, in large part, to an N-terminal low-complexity Ub binding region (LC-UBR) that binds the Ub Ile44 patch but lacks secondary structure. The cumulative structural and functional data enable construction of a robust family dendrogram for CE clan enzymes across kingdoms. Finally, we uncover the functional importance for accessory domains outside the CE fold that, in the case of SseL, serve to target the effector to *Salmonella*-containing vacuoles (SCVs) during infection. Our comprehensive analysis of bacterial proteases redefines the roles they play during infection and establishes a framework for cross-kingdom relationships among the entire CE clan.

## Results

### Selection and Properties of Bacterial CE Clan Effectors

**Figure 1:**
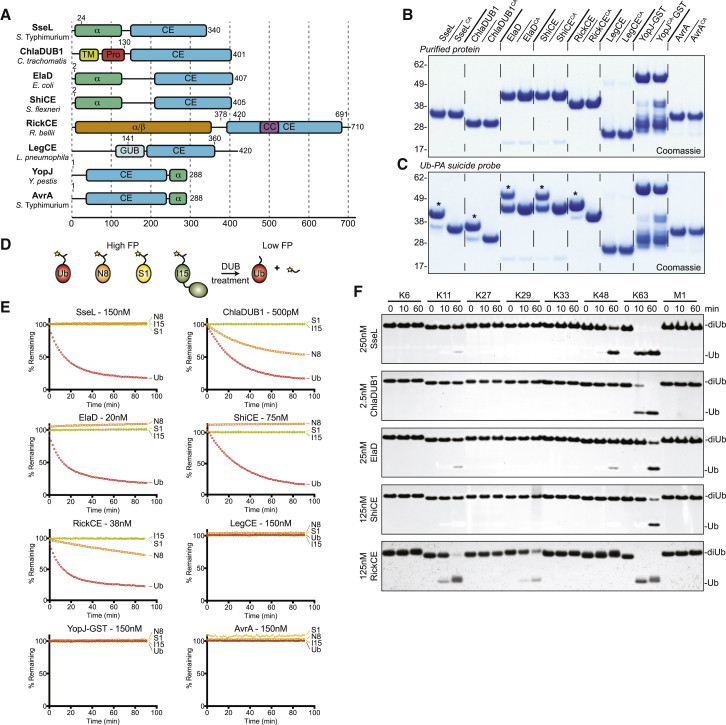
CE Effector Proteins Demonstrate Mixed Activities (A) Domain annotation of selected bacterial CE effectors. Construct boundaries and accessory domains used in this study are indicated. α, predicted α-helical domain; α/β, predicted α/β fold; TM, transmembrane helix; Pro, proline-rich sequence; GUB, wall-associated receptor kinase galacturonan-binding (GUB_WAK_bind) domain; CC, coiled coil. (B) Purified CE enzymes from bacteria. CA, catalytic Cys-to-Ala mutant. GST-YopJ contains co-purifying degradation products at lower molecular weight. (C) Suicide probe reaction following incubation with the Ub-PA probe for 1 hr at room temperature. Asterisks (^∗^) mark catalytic cysteine-dependent reactivity. (D) Schematic of the Ub/Ubl substrate cleavage assay. Ub/Ubl modifiers are isopeptide linked to a TAMRA-labeled Lys-Gly peptide, and fluorescence polarization (FP) is used to monitor cleavage over time. N8, NEDD8; S1, SUMO1; I15, ISG15. (E) Normalized FP measured as a function of time following the addition of the listed active CE enzymes to the TAMRA-linked Ub/Ubl reagents. (F) Linkage specificity analysis for bacterial CE DUBs active in the TAMRA assay. A silver-stained SDS-PAGE gel shows diUb hydrolysis over time. See also Figure S1.

While the presence of CE clan effectors in bacteria and biochemical functions for some members have been described, a comprehensive comparison of proteolytic specificities has not been performed. To fill this gap, we selected a divergent set of enzymes from human bacterial pathogens (Figure 1A), including putative DUBs SseL (*Salmonella enterica* serovar Typhimurium, hereafter *S*. Typhimurium [Rytkönen et al., 2007]), ChlaDUB1 (*Chlamydia trachomatis* [Misaghi et al., 2006]), and ElaD (*Escherichia coli* [Catic et al., 2007]), as well as Ser/Thr acetyltransferases YopJ (*Yersinia pestis* [Mukherjee et al., 2006, Mittal et al., 2006]) and AvrA (*S*. Typhimurium [Jones et al., 2008]). To this, we added entirely uncharacterized CE clan proteins RickCE (*Rickettsia bellii*, GenBank: ABE04279.1), LegCE (*Legionella pneumophila ssp. pneumophila*, GenBank: AAU28953.1), and ShiCE (*Shigella flexneri*; GenBank: EGK20985.1) (Figure 1A). In all of the selected enzymes, the catalytic CE fold is present in the context of additional domains of unknown function (Figures 1A and S1A), only few of which bear sequences similar to domains found in eukaryotes (see below). Following cloning and the subsequent *E. coli* expression of suitable constructs (Figure 1A), the panel of CE enzymes was purified to homogeneity (Figure 1B). The CE fold contains a conserved catalytic Cys (Figure S1B), which was mutated in each enzyme to Ala (e.g., SseL^CA^) and purified analogously (Figure 1B).

### Activities of CE Clan Bacterial Effectors

Cys-based DUBs can be covalently modified by Ub-based suicide probes, in which the C terminus has been modified with an electrophilic trap, such as propargylamine (Ub-PA; Ekkebus et al., 2013). SseL, ChlaDUB1, and ElaD, as well as the previously uncharacterized ShiCE and RickCE, were all covalently modified by Ub-PA in a catalytic Cys-dependent manner, while the remaining enzymes, including YopJ, AvrA, and LegCE, remained unmodified (Figure 1C).

To assess all Ub and Ubl proteolysis in a simple, parallel assay, we used fluorescence polarization (FP) to measure hydrolysis of Ub, SUMO1, NEDD8, and ISG15 substrates that were isopeptide linked to a fluorescently labeled Lys-Gly peptide (Figure 1D) (Geurink et al., 2012, Basters et al., 2014). Isopeptide linkage imposes a more chemically precise environment for a Ub/Ubl modification. When tested against the full panel, human SENP1 and NEDP1 exclusively cleaved SUMO1- and NEDD8-modified peptides, respectively (Figure S1C). YopJ, AvrA, and LegCE showed no proteolytic activity in our assays, regardless of the presence of the cofactor inositol hexakisphosphate (IP6) (Mittal et al., 2010) (Figures 1E and S1D–S1F). Furthermore, AvrA showed no sign of an interaction with Ub when measured by nuclear magnetic resonance (NMR) titration (Figure S1G). YopJ and AvrA were, however, highly active in an in vitro auto-acetylation assay with IP6, and LegCE, likewise, showed modest acetylation activity compared to its catalytically inactive mutant (Figure S1H), indicating that these effectors are dedicated acetyltransferases.

Interestingly, the remaining bacterial effectors SseL, ElaD, and ShiCE are Ub-specific proteases, and ChlaDUB1 and RickCE cleave both Ub- and, to a lesser extent, NEDD8-modified peptides (Figure 1E). Like NEDP1, ChlaDUB1 can remove the C-terminal five amino acids from pro-NEDD8 with peptidase activity (Figure S1I), but does not cleave the regulatory NEDD8 modification from the cullin RING ligase adaptor Cul1 (Figure S1J).

As there are many examples of interesting polyUb chain specificity in eukaryotic DUBs (Mevissen et al., 2013), it was important to test if any bacterial CE DUBs showed a preference for particular polyUb substrates. Early studies on SseL indicated higher activity for K63-linked over K48-linked chains (Rytkönen et al., 2007), and *Legionella pneumophila* effector SdeA prefers K63 linkages over K11 and K48 linkages (Sheedlo et al., 2015), but preference among all eight possible Ub:Ub linkages for these and all other enzymes have remained unstudied. Strikingly, we found that CE DUBs encoded by human pathogens showed strong preference for K63-linked chains, only targeting K48 and K11 chains at later time points or higher enzyme concentrations (Figures 1F and S1K–S1M), indicating significant pressure to remove this important infection-associated post-translational modification during invasion (Corn and Vucic, 2014).

### Structural Analysis of the CE Proteases

**Figure 2:**
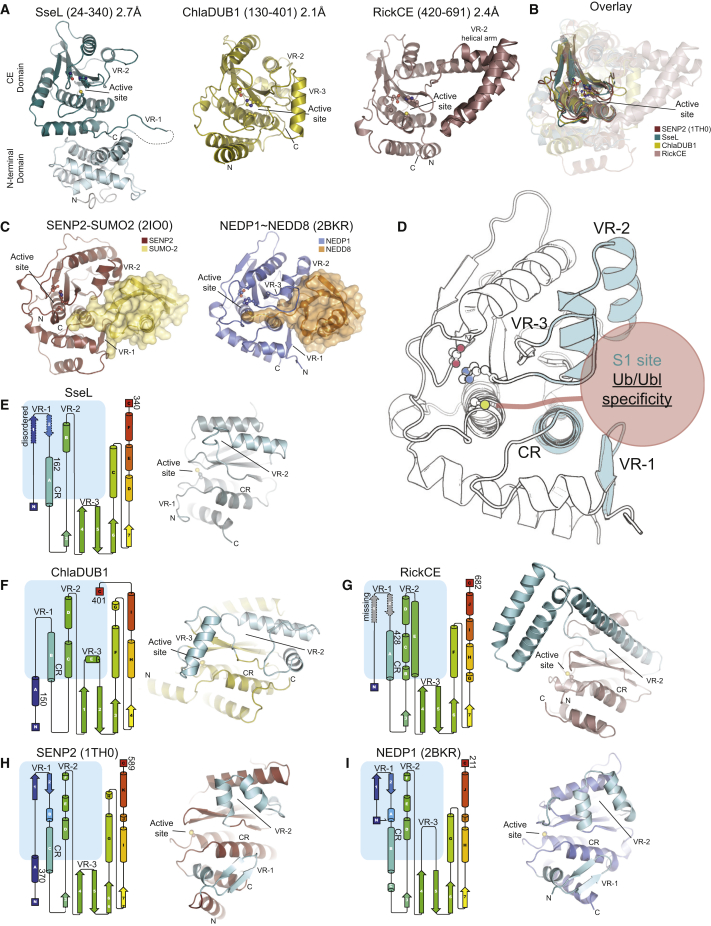
Structural Analysis of Bacterial CE Deubiquitinases (A) Cartoon representations of SseL (2.7 Å, teal), ChlaDUB1 (2.1 Å, yellow), and RickCE (2.4 Å, violet) aligned on the catalytic triad (ball and stick). (B) Superposition of the CE core based on the catalytic triad for structures in (A) and human SENP2 (PDB: 1TH0). (C) Representations of the human SENP2-SUMO2 noncovalent complex (PDB: 2IO0, left) and NEDP1∼NEDD8 covalent complex (PDB: 2BKR, right) illustrating a common Ubl binding site. (D) Schematic of the CE fold based on the NEDP1 structure (PDB: 2BKR), highlighting the constant region (CR) and variable regions (VR) that form the Ub/Ubl-binding S1 site (blue). (E–I) Left: topology diagrams for CE structures, with the S1 binding site highlighted (blue box). Structure boundaries are indicated by numbers. Features in dashed outline are either disordered or outside the crystallized constructs, as marked. Right: view of the S1 substrate binding site in CE-fold structures, with variable regions highlighted in cyan and labeled accordingly. See also Table 1 and Figure S2.

The new functionalities in CE clan effectors prompted structural characterization to reveal how this enzyme fold evolved to accommodate such divergent proteolytic activities. We determined high-resolution crystal structures of the CE clan effectors SseL, RickCE, and ChlaDUB1 (Figures 2A and S2A–S2C; Table 1). Due to low-sequence similarities, all structures were experimentally phased (see the Experimental Procedures). The crystallized full-length SseL construct comprised an additional N-terminal ∼135-residue helical domain (see below). The catalytic CE fold bears similarity to eukaryotic CE proteases (Figure 2B), and the conserved active-site residues are required for DUB activity (Figures S2D and S2E). However, several regions of high variability were immediately apparent.

A comparison to the previously determined SENP2-SUMO2 and NEDP1∼NEDD8 complexes (Figure 2C) (Reverter and Lima, 2004, Reverter et al., 2005, Shen et al., 2005) revealed differences among bacterial CE DUBs in three variable regions (VRs; Figure 2D) near the substrate binding (S1) site that were arranged around a structurally conserved helix (constant region, CR). Variations within these three regions are found in all examples of the CE fold (Figures 2E–2I), as detailed below.

The first variable region, VR-1, is found at the beginning of the CE fold (Figure 2D), which in NEDP1 and the SENP family is a β-hairpin that forms electrostatic interactions with the Ubl (Figures 2C, 2H, and 2I). In bacterial examples, the analogous region is either disordered in the absence of substrate (SseL; Figures 2A and 2E) or not part of the crystallized construct (RickCE; Figures 2A and 2G). A corresponding VR-1 region is absent in ChlaDUB1; interestingly, the ChlaDUB1 C terminus occupies this space instead (Figures 2A and 2F).

The second variable region, VR-2, is located between the first two strands of the central β sheet, and in the eukaryotic examples forms hydrophobic contacts with the Ubl β sheet (Figure 2D). Intriguingly, this region is similar among the Ubl proteases (Figures 2H and 2I), but diverse among the CE-fold DUBs (Figures 2E–2G). In fact, RickCE has an ∼75-residue insertion at this site that forms a helical arm in the crystal structure (Figure 2A).

The third variable region, VR-3, is an insertion between the second and third strands of the β sheet, just preceding the catalytic His (Figure 2D). NEDP1 and ChlaDUB1 contain an insertion of 7 and 24 residues, respectively, in what is a short β-turn in the other structures (Figures 2A, 2C, 2F, and 2I). In NEDP1, VR-3 forms an extended loop that guides the NEDD8 C terminus into the active site through backbone hydrogen bonding (Figures 2C and 2I). In contrast, ChlaDUB1 forms a helix that may serve a similar purpose in recognition of the Ub C terminus (Figures 2A and 2F).

Overall, the dramatic differences in substrate recognition imparted by CE-fold variable regions suggest that bacteria have adopted different strategies of altering the CE-fold to evolve DUB activity.

### Re-evaluation of XopD Ub/Ubl Specificity

**Figure 3:**
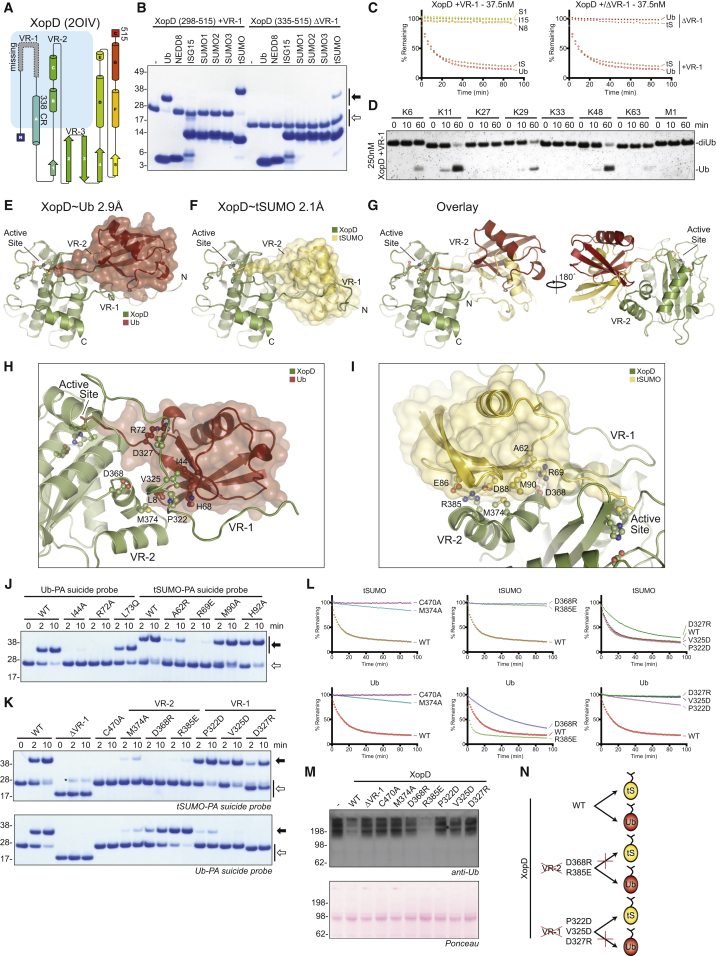
Molecular Analysis of XopD Ub/Ubl Specificity (A) Topology diagram, as in Figure 2E, for the XopD crystal structure (PDB: 2OIV). The crystallized construct lacks a potential VR-1 region. (B) XopD constructs with VR-1 (+VR-1: 298–515) or without VR-1 (ΔVR-1: 335–515) were tested against a panel of Ub/Ubl suicide probes for 1 hr at room temperature (propargylamine-derived probes). Open arrow, unmodified; closed arrow, modified. (C) FP assays as in Figure 1D, including a substrate derived from *S. lycopersicum* SUMO (tS, tomato SUMO). Left: Ub/Ubl specificity of XopD +VR-1. Right: comparison of XopD +VR-1 cleaving the Ub and tSUMO substrates (shown in left) with the ΔVR-1 construct. (D) Linkage specificity analysis as in Figure 1F for XopD +VR-1. (E) Cartoon representation of the 2.9 Å XopD∼Ub covalent complex crystal structure. (F) Cartoon representation of the 2.1 Å XopD∼tSUMO covalent complex crystal structure. (G) Overlay of structures shown in (E) and (F), illustrating differences in Ub/tSUMO conformation and usage of XopD variable regions. For clarity, VR-1 is not shown. (H) Close-up of the XopD-Ub interaction. In the foreground, VR-1 coordinates Ub Arg72 and the Ile44 hydrophobic patch. In the background, VR-2 contacts the Ub Leu8 loop. (I) Close-up of the XopD-tSUMO interaction. VR-2 forms the basis of tSUMO binding and makes a number of polar and salt-bridge interactions surrounding the hydrophobic contact of tSUMO Met90. (J) Series of Ub and tSUMO suicide probe reactions performed on ice, testing interactions observed in the Ub/tSUMO domain, as well as in their C termini (propargylamine-derived probes). Open arrow, unmodified; closed arrow, modified. (K) As in (J), for XopD mutations encompassing VR-1 and VR-2. Open arrow, unmodified; closed arrow, modified. Asterisk, contamination in tSUMO suicide probe. (L) FP-based cleavage assays testing the XopD variants used in (K) against the Ub and tSUMO KG-modified substrates. Wild-type XopD data from (C) are shown for reference. (M) *S. lycopersicum* protein extract blotted for total Ub (Ubi-1; Novus Biologicals) following a 1 hr room temperature treatment with the XopD variants used in (K) at 1 μM final concentration. (N) Schematic summarizing the activities displayed by wild-type and VR-1/VR-2 mutant XopD variants. See also Table 1 and Figure S3.

During our analysis of the CE fold, we noticed that a putative VR-1 region had been omitted in the crystallized construct of the *Xanthomonas campestris* effector XopD (Figure 3A) (Chosed et al., 2007). Given the importance of all three variable regions in the eukaryotic Ubl-bound structures (Figures 2C, 2H, and 2I), we compared the activity of XopD without (ΔVR-1; amino acids [aa] 335–515) and with the analogous VR-1 region (+VR-1, aa 298–515). In a suicide probe assay, XopD ΔVR-1 reacted solely with *Solanum lycopersicum* tomato SUMO (tSUMO), but not with human SUMO1, as reported (Chosed et al., 2007) (Figure 3B). To our surprise however, XopD +VR-1 was not only more reactive with tSUMO but also strikingly now reacted with Ub (Figures 3B and S3A). The same Ub/tSUMO dual specificity was observed in an FP-based assay of KG-modified substrates, where both Ub and tSUMO were cleaved with similar high efficiencies in a VR-1-dependent manner (Figure 3C). To our knowledge, XopD is the first cross-reactive Ub and (t)SUMO isopeptidase. Moreover, unlike other CE effector DUBs, XopD prefers K11, K29, and K48 linkages and only cleaves K63- and K6-linked chains to a lesser extent (Figure 3D). This distinct specificity profile for Ub chain substrates highlights that the versatile CE fold can not only be adapted to distinct Ub/Ubl specificities (which depends on S1 site interactions, see above) but also can be adapted to modulate Ub chain preferences, due to changes in the S1’ site (see below). Biologically, this reiterates the pressure placed on human pathogens to deal with K63 linkages, while the plant pathogen clearly prioritizes distinct linkages and SUMO, which may, hence, mediate anti-bacterial signaling in plants.

### Molecular Analysis of XopD Cross-Specificity

To understand what structural adaptations enable XopD cross-reactivity, we determined the crystal structures of covalent XopD∼Ub (2.9 Å; Figures 3E and S3B; Table 1) and XopD∼tSUMO (2.1 Å; Figures 3F and S3C; Table 1) complexes. The XopD∼Ub structure contained four copies in the asymmetric unit, all of which were similar (0.62 Å root-mean-square deviation [RMSD] over XopD and Ub; Figure S3D). Moreover, the core fold of XopD itself was highly similar in the Ub-bound, tSUMO-bound, and the published apo structure (PDB: 2OIV, 0.61 Å RMSD over aa 338–515; Figure S3E). To our surprise, the VR-1 responsible for much of the observed XopD protease activity was not a β-hairpin as had been observed in eukaryotic Ubl proteases (Figure 2D), but, instead, was extended and devoid of secondary structure. Furthermore, the extended VR-1 conformation was completely different between the Ub- and tSUMO-bound complexes. A comparison of the two structures clearly explains the observed VR-1 dependence in activity. While the Ub S1 site primarily consists of contacts to VR-1 and some to VR-2, tSUMO binding displays the opposite trend, with the majority of interactions taking place at VR-2 (Figures 3E–3G).

In the Ub-bound structure, XopD VR-1 threads underneath the Ub Ile44 hydrophobic patch, with residues Pro322 and Val325 making contacts to Ub Ile44 and His68, and also coordinates Ub Arg72 with Asp327 (Figure 3H, foreground). Additionally, Met374 of VR-2 contacts the Leu8 loop of Ub (Figure 3H, background). Incorporation of either I44A or R72A mutations into Ub suicide probes resulted in a dramatic loss in reactivity to XopD (Figure 3J).

The VR-1 interface to the Ub Ile44 patch was highly unusual and unprecedented among published ubiquitin-binding domains, because such a low-complexity Ub binding region (LC-UBR) had not been previously described. Verifying the interaction in solution, NMR titration experiments following ^15^N-labeled Ub revealed a number of select resonances that displayed small chemical shift perturbations and/or line broadening with increasing concentrations of the XopD +VR-1 construct (Figure S3G), all of which depended on the VR-1 region (Figure S3H). When mapped to the XopD∼Ub crystal structure, the changes observed in the NMR titration experiment nicely agree with contacts made between XopD VR-1 and the Ub Ile44 patch (Figure S3I).

In contrast, the bulk of contacts made in the tSUMO-bound structure are through VR-2, which forms multiple ionic interactions (XopD Arg385 and Asp368 contacting tSUMO Glu86/Asp88 and Arg69, respectively) surrounding a hydrophobic interface (XopD Met374 contacting tSUMO Met90) (Figure 3I). The tSUMO A62R or R69E mutation both resulted in a decrease in tSUMO suicide probe reactivity (Figure 3J). Surprisingly, a M90A mutation had much smaller effects (Figure 3J), suggesting that the small hydrophobic interface may play a lesser role in tSUMO recognition than does the extensive charge complementarity. Although highly divergent in sequence, the Ub and tSUMO C termini threaded into the active site similarly, with no apparent favoritism following Ub Arg72 (Figure S3F). Accordingly, neither a swap of Ub Leu73 to the analogous tSUMO Gln nor mutation of tSUMO His92 (equivalent to Ub Arg72) had a significant impact on suicide probe reactivity (Figure 3J).

### Manipulating XopD Ub/Ubl Preferences

The Ub/tSUMO-bound structures were further confirmed by mutagenesis targeting interactions in VR-1 and VR-2 that were either common to both substrates or unique to one. In addition to the catalytic C470A mutation, a XopD M374A mutation in VR-2 was deleterious to both Ub and tSUMO recognition, as observed in a suicide probe assay (Figure 3K) and FP-based protease assay (Figure 3L). Additional mutations in VR-2 targeted the primary tSUMO interaction surface and resulted in either a slight loss (D368R) or a gain (R385E) in activity toward Ub substrates, while activity toward tSUMO was significantly reduced (Figures 3K–3L). In contrast, mutations targeting the primary Ub interaction in VR-1 (P322D, V325D, and D327R) all showed decreased activity toward Ub substrates with little-to-no effect on tSUMO recognition (Figure 3K–3L). Furthermore, recombinant XopD hydrolyzes endogenous Ub-modified proteins from *S. lycopersicum* protein extract in a manner consistent with the mutational analysis on biochemical substrates (Figure 3M).

Thus, the cross-reactive XopD uses adaptations of common variable regions (see Figure 2D) to specifically recognize both Ub and tSUMO. In particular VR-1 forms contacts unique to each substrate (Figures 3E, 3F, and 3N). The proline-rich, low-complexity VR-1 sequence bears a textbook resemblance to an intrinsically disordered region (mean IUPred disorder propensity score of 0.72 [Dosztányi et al., 2005]) and thus enables flexible binding properties ideally suited to provide dual specificity.

### Understanding Ub/Ubl Specificity in CE Clan DUBs

**Figure 4:**
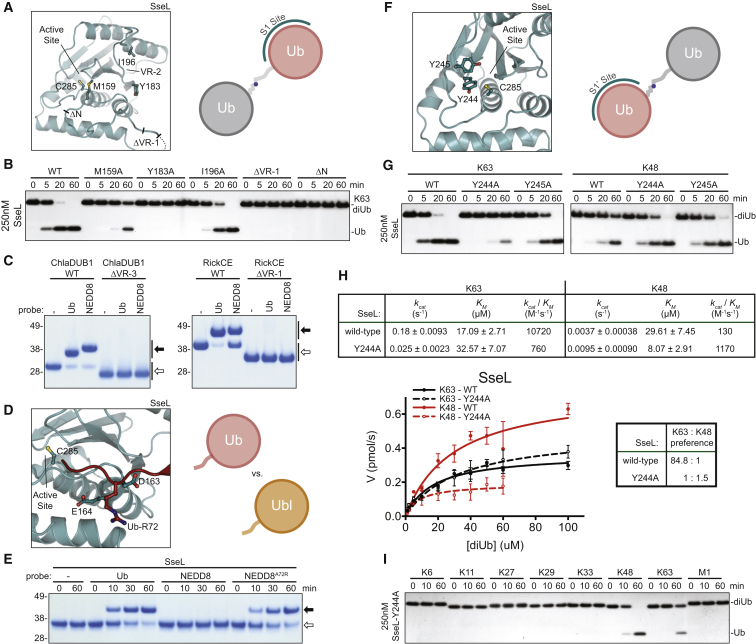
Structure-Based Manipulation of CE DUB Activities and Specificities (A) Mutations and truncations designed in the SseL S1 binding site (SseL ΔVR-1: Δ138–147; SseL ΔN: aa 158–340). (B) K63 diUb cleavage assay using mutants from (A). (C) Suicide probe assay monitoring Ub and NEDD8 reactivity following 1 hr incubation at room temperature, for wild-type ChlaDUB1 and RickCE, and constructs containing truncated variable regions (ChlaDUB1 ΔVR-3: Δ250-272; RickCE ΔVR-1: aa 420-691; propargylamine-derived probes). Open arrow, unmodified; closed arrow, modified. (D) Model of the Ub C terminus entering the SseL active site, based on analogous Ubl-bound structures. Putative acidic residues that can coordinate Ub Arg72 are shown. (E) SseL suicide probe assay with Ub, NEDD8, and NEDD8 A72R probes performed at room temperature (chloroethylamine-derived probes). Open arrow, unmodified; closed arrow, modified. (F) Close-up of the conserved hydrophobic S1’ site of SseL. (G) K63 and K48 diUb cleavage assays using S1’ site mutations. (H) Kinetic analysis of K48 and K63 diUb linkage preference for SseL wild-type and Y244A. (I) Linkage specificity analysis for all diUb substrates, as in Figure 1F, for the SseL Y244A S1’ site mutant. See also Figure S4.

The importance of variable regions in Ub recognition extends to each crystallized DUB (Figures 2E–2G) and also to the *Legionella* effector SdeA, which uses yet another variation of VR1-3 to recognize Ub (Sheedlo et al., 2015) (Figures S4A and S4B). Mutations in the S1 site of SseL confirmed the Ub binding mode predicted based on analogous Ub/Ubl-bound structures (Figure 4A). A conserved hydrophobic (Trp or Phe) at the start of the core CE fold is present in eukaryotic CE ULPs and corresponds to Met159 in SseL. SseL M159A mutation significantly reduced K63 diUb hydrolysis (Figures 4A and 4B). Likewise, solvent-exposed hydrophobic residues within SseL VR-2 (Tyr183 and Ile196) abolished or reduced DUB activity, respectively (Figures 4A and 4B). Interestingly, deletion of the putative substrate-binding VR-1 region alone (SseL ΔVR-1) or removal of the N-terminal domain including VR-1 (SseL ΔN) abolished diUb cleavage, suggesting that the disordered sequence indeed contributes to substrate binding (Figures 2E, 4A, and 4B).

The importance of unique variable regions in ChlaDUB1 and RickCE was also tested using Ub/Ubl suicide probes as a measure of substrate recognition. Matching their specificity profiles (Figure 1E), ChlaDUB1 and RickCE can both form covalent adducts with Ub and NEDD8 suicide probes. Deletion of the inserted VR-3 helix of ChlaDUB1 (Figures 2A and 2F) or the predicted VR-1 outside the crystallized boundaries of RickCE (Figures 2A and 2G) resulted in a total loss in recognition of the Ub/Ubl probes (Figure 4C). In sum, the unique contributions from all three variable regions in XopD, SseL, ChlaDUB1, and RickCE are required for recognition of a Ub/Ubl substrate.

### Changing Specificities for Ub/Ubl and polyUb Chain Linkage

Ub and NEDD8 are highly similar (58% identical, 85% similar), yet most CE-clan proteases, including SseL and XopD, distinguish between them with high specificity (Figures 1E, S1C, and S3C). A key difference between Ub and NEDD8 is an Arg-to-Ala substitution at position 72 in the Ub/Ubl C terminus. Arg72 of Ub is well-coordinated in the XopD∼Ub structure (Figure 3H), as well as in other Ub-bound DUB structures (Ye et al., 2011). Analysis of a modeled SseL∼Ub complex highlighted two acidic residues, Asp163 and Glu164, that may also coordinate Ub Arg72 (Figure 4D). Importantly, a NEDD8 suicide probe with an A72R mutation (Ye et al., 2011) covalently modified SseL as efficiently as the wild-type Ub probe (Figure 4E). Likewise, incorporation of the A72R mutation imparts reactivity of the NEDD8 suicide probe toward XopD (Figure S4C). Thus, in addition to large-scale changes in the variable regions of the S1 site, proper coordination of the Ub/Ubl C terminus contributes an added level of substrate specificity.

Unlike XopD (Figure 3D), CE effectors from human pathogens strongly prefer K63-linked chains (Figure 1F). This suggests there must be a linkage specificity-imposing S1’ Ub binding site that participates in Ub recognition (Mevissen et al., 2013), though such an S1’ site in CE DUBs has not been annotated. Examination of the SseL, ChlaDUB1, and RickCE structures revealed a conserved hydrophobic region near the catalytic Cys (Figures 4F and S4D), and mutating residues in this patch on SseL simultaneously reduced activity toward K63-linked diUb, while improving hydrolysis of K48-linked diUb (Figure 4G). This was quantified by a fluorescence polarization-based diUb cleavage assay (Keusekotten et al., 2013). Based on the Michaelis-Menten kinetic parameters, wild-type SseL displays an approximately 85-fold preference for K63 chains over K48; this is predominantly the result of a difference in *k*_*cat*_ (Figure 4H). The Y244A mutation causes an approximately 15-fold reduction in *k*_*cat/*_*K*_*m*_ toward K63-linked chains, while simultaneously increasing *k*_*cat/*_*K*_*m*_ toward K48-linked chains 10-fold. Hence, SseL Y244A displays a ∼1.5-fold preference for K48 over K63 diUb (Figure 4H); this preference is also confirmed when tested against a panel of all eight Ub chain types (Figure 4I).

Together, mutagenesis work establishes the location of the S1’ Ub binding site in CE clan DUBs, and the ability to modulate Ub/Ubl, as well as linkage specificity with point mutations, highlights the versatility and adaptability of the CE protease fold.

### Bioinformatic Analysis of the CE Clan Highlights Distinct Functional Families

Using our new CE protease structures as a guide, we expanded and refined the sequence alignment of CE clan members in eukaryotes, viruses, and bacteria (Figure S5). In addition to the active site, we identified several conserved positions important for domain structure and substrate recognition, including the semi-conserved Trp at the start of the CE fold and a highly conserved Trp following the catalytic His.

**Figure 5:**
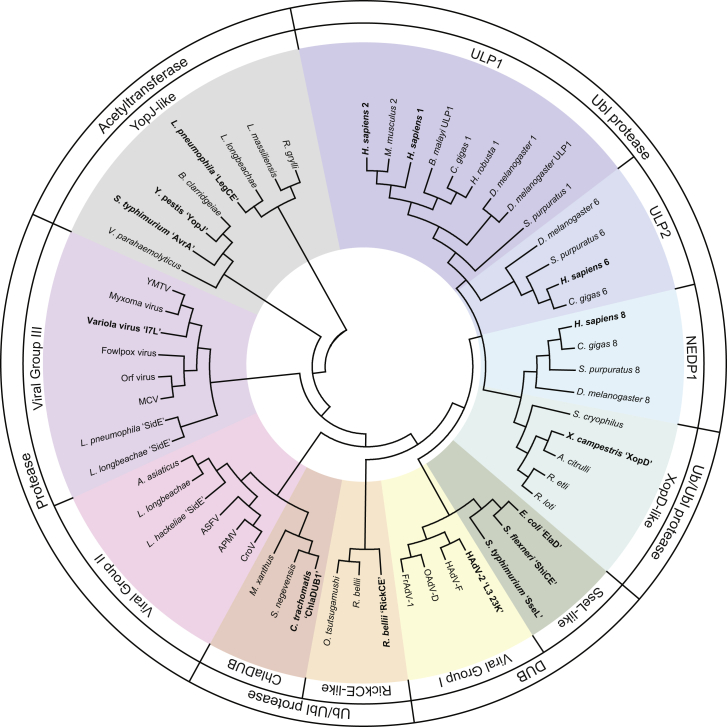
Bioinformatic Analysis of the CE Protease Clan Structure-informed dendrogram for the CE clan, with representative members from eukaryotes, bacteria, and viruses. Clustered families are highlighted and labeled according to the archetypal examples. Eukaryotic family members are labeled with numbers corresponding to their SENP nomenclature. Characterized members are highlighted in bold. See also Figure S5.

Rewardingly, reconstruction of the dendrogram for the entire CE clan shows an interesting segregation of families that agrees with the demonstrated functional properties for these enzymes (Figure 5). In agreement with their dual Ub/SUMO protease activity, the XopD-like family, including XopD and examples from other plant-associated bacteria, are most closely related to the eukaryotic ULP families. As dedicated DUBs, the SseL-like examples (including ElaD and ShiCE) also segregate near the XopD/ULP subfamily. The Ub/NEDD8 cross-reactive enzymes ChlaDUB1 and RickCE fall into their own clade. Interestingly, the YopJ family, that also includes *Legionella* LegCE, lacks, e.g., conserved Trp residues (Figure S5) and is most divergent, consistent with their roles as dedicated acetyltransferases (Figures 1E and S1H).

CE fold proteins from viruses constitute three additional groups. The first group (viral group I) includes examples from Adenoviridae and clusters near the Ub/Ubl proteases. This group includes the adenovirus L3 23K proteinase, which, in addition to general protease activity, also possesses DUB activity (Balakirev et al., 2002).

Viral groups II and III are more divergent and also include bacterial proteins (Figure 5). The *Legionella* effector SidE (Figures S4A and S4B) was independently identified by our sequence analysis as a part of viral group II and has since been shown to display mixed activities toward Ub, NEDD8, and, interestingly, ISG15 (Sheedlo et al., 2015). Viral group III, which contains Vaccinia virus protein I7L, has been shown to target an Ala-Gly-Xaa motif instead of the Gly-Gly-Xaa motif of Ub/Ubl modifiers, and hence, members within the viral group III might be more general proteases dedicated to cleavage events necessary for viral maturation (Byrd et al., 2003). Therefore, our structural and functional analysis of divergent CE clan members has enabled the refinement of relationships across kingdoms, has enabled identification of further examples in other species, and allows a distinction between dedicated DUBs, dedicated ULPs, and specialized acetyltransferases.

### CE Clan Proteases Are Further Functionalized with Accessory Domains

In addition to the catalytic fold, bacterial CE clan effectors contain additional N- or C-terminal domains of high diversity and unknown functions. These range from predicted transmembrane regions, protein-protein interaction or regulatory domains, and, even, additional enzymatic folds (Figures 1A and S6A) and likely represent an additional layer of regulation, similar to eukaryotic ULP enzymes (Ronau et al., 2016).

**Figure 6:**
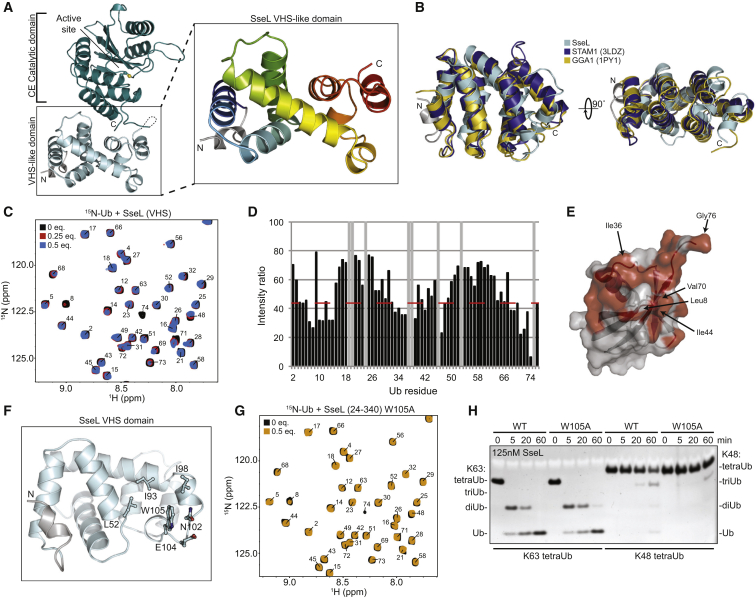
CE Catalytic Domains Are Fitted with Accessory Domains (A) SseL crystal structure (active-site Cys in a ball-and-stick representation), focusing on the superhelical N-terminal VHS domain. (B) Superposition of the SseL N-terminal domain (cyan) with the VHS domains of STAM1 (PDB: 3LDZ, dark blue), and GGA1 (PDB: 1PY1, orange). (C) ^1^H,^15^N-HSQC TROSY spectra of 80 μM ^15^N-labeled Ub alone (black) and in the presence of 0.25 (20 μM, red) or 0.5 (40 μM, blue) molar equivalents of the SseL VHS domain (see the Supplemental Experimental Procedures). Numbers refer to the assigned Ub residues. (D) Calculated ratio in peak intensity observed between ^15^N-labeled Ub alone and in the presence of 0.5 molar equivalent SseL VHS domain. A red dashed line marks the level of significance used in (E). Gray bars indicate prolines or missing resonances. (E) Surface representation of the Ub structure (PDB: 1UBQ) with red painted surface corresponding to regions significantly affected by SseL VHS binding. (F) Exposed hydrophobic patch within the SseL VHS domain; W105 was chosen for mutation. (G) ^1^H,^15^N-HSQC TROSY spectra of 80 μM ^15^N-labeled Ub alone (black) and in the presence of 0.5 molar equivalents of SseL (24–340) W105A (40 μM, orange). (H) Time course assays monitoring cleavage of K63- and K48-linked tetraUb chains with the SseL VHS domain Ub-binding mutant. See also Figure S6.

The crystal structure of full-length SseL revealed an unannotated N-terminal domain, in which eight helices form a superhelical structure (Figure 6A). Comparison with known structures using the Dali server (Holm and Rosenström, 2010) indicated similarity with the VHS (VPS-27, Hrs, and STAM) domains of GGA1 (PDB: 1PY1, [He et al., 2003], Dali *Z* score of 5.0, RMSD 4.0 Å), and STAM1 (PDB: 3LDZ [Ren and Hurley, 2010], Dali *Z* score of 4.2, RMSD 3.8 Å) (Figure 6B). In eukaryotes, VHS domains are involved in vesicular trafficking, either through direct interaction with a phosphorylated receptor (e.g., GGA1 [Shiba et al., 2002]) or by binding Ub attached to cargo (e.g., STAM1 [Ren and Hurley, 2010]).

VHS domains, so far, are unknown to occur in bacteria, and the functional peptide- and Ub-binding interfaces differ in the SseL VHS domain. SseL did not interact with phosphorylated or unphosphorylated mannose-6-phosphate receptor (MPR) peptides (Figure S6B). In contrast, NMR titration experiments of the isolated SseL VHS domain revealed low micromolar interactions with the Ile36 and Ile44 hydrophobic patches of Ub (Figures 6C–6E and S6C). Identical Ub binding was also observed with an SseL construct including the CE fold (aa 24–340) (Figure S6D), and this was unaffected by mutations in the S1 Ub binding site or when a covalent SseL∼Ub complex was used in the titration (Figures S6E and S6F), suggesting that the VHS domain constitutes the highest affinity Ub binding site.

Inspection of the SseL VHS structure identified two solvent-exposed hydrophobic patches centered on Leu46 and Trp105 as potential Ub-interacting surfaces (Figures 6F and S6G). Ub binding was unaffected with a SseL L46R mutant (Figure S6H), but abrogated in a SseL W105A mutant (Figure 6G). This Ub binding site on SseL is distinct from that on the STAM1 VHS domain and remote from the catalytic center (Figure S6I). The SseL W105A mutant had no effect on Ub chain hydrolysis for K63-linked di-, tri-, or tetra-Ub (Figures 6H and S6J). This suggested that the added Ub binding site outside of the SseL catalytic domain does not contribute to the recognition or hydrolysis of longer Ub chains, but may instead serve alternative roles in regulating SseL during *Salmonella* infection.

### The SseL VHS Domain Dictates Subcellular Localization of the Effector

**Figure 7:**
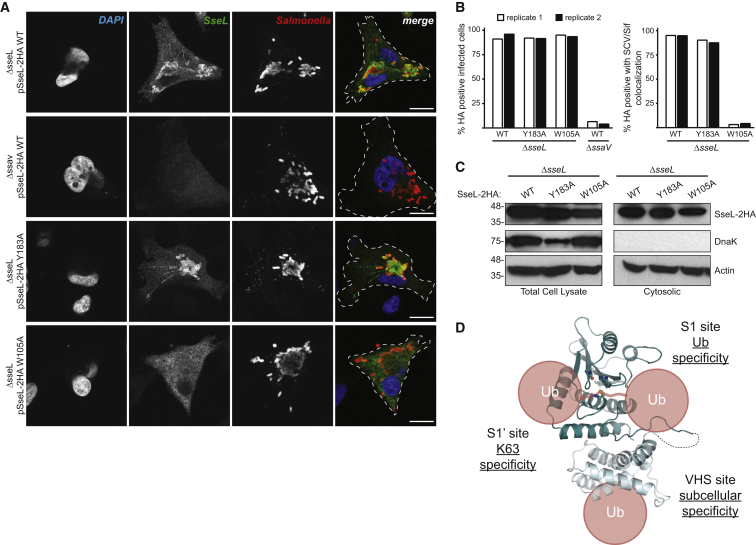
The SseL Accessory VHS Domain Dictates Subcellular Localization (A) Representative images of HeLa cells infected with *S*. Typhimurium strains expressing 2HA-tagged wild-type or mutant SseL, in the *ΔsseL* or *ΔssaV* (SPI-2 T3SS null) background. Cells were immunostained with HA (SseL) and CSA-1 (*S*. Typhimurium), and DNA was stained with DAPI. White bar, 10 μM. (B) Quantitation of (A). Infections were performed in duplicate (white and black bars). 200 cells from each replicate of each infection were assessed for SseL localization to SCV and Sifs. (C) Cytosolic and total cell lysate fractions of infections performed as in (A), separated by SDS-PAGE, and immunoblotted for HA (SseL), DnaK, and Actin. (D) Model demonstrating the multiple Ub binding modes exhibited by SseL and their effects on various levels of specificity. See also Figure S7.

Following translocation from the *Salmonella*-containing vacuole (SCV), SseL robustly localizes to the outer leaflet of the vacuolar membrane, as well as to *Salmonella*-induced filaments (Sifs) that emanate from the SCV (Rytkönen et al., 2007). To determine if this localization is determined by the Ub-binding activity of the VHS domain, we reintroduced HA-tagged versions of wild-type (SseL-2HA WT), S1 site mutant (SseL-2HA Y183A), and VHS mutant (SseL-2HA W105A) into the *Salmonella* Typhimurium Δ*sseL* strain to be expressed from a plasmid under the native promoter. HeLa cells were infected with the complemented strains for 16 hr, then fixed, and immune-labeled for SseL-2HA and CSA-1 (a marker for bacteria). Wild-type SseL localized to SCVs and Sifs in a SPI-2 type 3 secretion system (T3SS)-dependent manner (compared to *ΔssaV* T3SS-deficient control), as expected (Figure 7A). The localization of S1 site mutant Y183A was indistinguishable from wild-type, indicating that Ub binding to the catalytic domain does not dictate subcellular localization (Figures 7A–7C and S7A). The W105A VHS mutant, however, showed a severe localization defect and was distributed diffusely throughout the host cytoplasm (Figures 7A–7C and S7A). Therefore, the N-terminal VHS domain of SseL serves to localize its catalytic DUB function through an additional Ub binding site (Figure 7D). Both ElaD and ShiCE encode similar N-terminal VHS domains and the Ub binding site is conserved (Figures 1A and S1A). Hence, we would predict that this subfamily of effectors has adopted analogous strategies of regulation.

## Discussion

During infection, bacteria secrete effector proteins, such as CE clan proteins, which inactivate inflammatory signaling cascades and promote bacterial growth. CE clan effectors were reported to be deSUMOylases, deubiquitinases, and, even, acetyltransferases, contrasting the specific and important roles of these enzymes in removal of a small subset of Ub-like modifications (SUMO and NEDD8) in eukaryotes. This ambiguity triggered the here presented comprehensive characterization of bacterial CE effectors, revealing dedicated DUBs and dedicated acetyltransferases, but also mixed Ub/Ubl proteases (Ub/NEDD8 and strikingly also Ub/SUMO). All this is performed by a single, highly adaptable catalytic fold. Complementary bioinformatic analysis based on structural data expands the family and reveals some of the functional differences. For example, we have annotated and characterized the first DUB of *Shigella*, an activity that has been inconspicuously lacking considering its large repertoire of effector E3 ligases (Ashida et al., 2014). However, the functional variation observed among CE clan members is staggering, making some enzymatic features unpredictable, especially when mixed activities are suspected.

DUB activity in CE effectors has been suggested to exist in various bacteria, and while we now understand strategies for adaptation of Ub specificity in molecular detail, it is surprising that bacteria have adopted such distinct structural approaches to generate a Ub-specific S1 site. This suggests the convergent evolution of distinct bacterial DUBs. Based on our structural work, we identify three common regions of variability that, together, encode Ub/Ubl substrate specificity. In our minds, the most surprising insight is the mixed activity of XopD being a (tomato−) SUMO and Ub isopeptidase, since these modifiers are quite distinct and the capacity to efficiently recognize both substrates suggests a large evolutionary pressure to become a multifunctional protease. Interestingly, our structural analysis of XopD bound to either tSUMO or Ub demonstrates that XopD’s flexibility in substrate recognition primarily arises from incorporation of an intrinsically disordered VR-1, which can be tailored to suit either binding event.

Moreover, we now understand the preference of most CE DUBs for K63-linked chains because of the presence of a defined, linkage-specificity-generating S1’ site. This site on CE proteases was unanticipated and is unknown for the CE ULPs; whether the six SENP members in humans preferentially target distinct SUMO chains is unclear. Our mutagenesis data further show that the observed K63 specificity hinges on several key contacts, and changes in these can switch specificity. Hence, we would anticipate that CE DUBs could target other chain types, if advantageous for infection (see XopD; Figure 3D).

Finally, the CE folds of most effectors are accessorized with additional domains, and we have shown that a Ub-binding VHS domain in SseL (and by sequence similarity in ElaD and ShiCE) mediates localization of the effector. The VHS domains of ESCRT-0 components STAM and Hrs bind ubiquitinated cargo to direct the lysosomal maturation pathway (Ren and Hurley, 2010). Similarly, the VHS domain of SseL binds Ub (albeit via a distinct surface) and functions in localizing it to Sifs and to the cytosolic face of the SCV, where the catalytic CE domain can remove K63-linked polyUb. This could block recruitment of xenophagy adaptors, such as p62 (Mesquita et al., 2012), through general removal of K63-linked chains, or directly target specific host proteins, such as oxysterol-binding protein (OSBP) (Auweter et al., 2012). Furthermore, given that many of these accessory domains are unrelated across CE clan members (Figures 1A and S6A), the breadth of possibilities through which CE enzymatic activities may be regulated is likely extensive. Our study serves as a framework to further investigate this intriguing class of effectors and, in particular, highlights several interesting mechanisms by which bacterial effectors encode Ub/Ubl specificity.

## Experimental Procedures

### Cloning, Expression, and Purification of the CE Effectors

Protein sequences were either cloned from bacterial or synthesized DNA or were received as gifts from colleagues. Constructs were generated as outlined in Figure 1A in the pOPIN-B or pOPIN-K vectors and purified using affinity, anion exchange, and size exclusion chromatography.

### DUB Activity Assays

Qualitative DUB assays, suicide probe assays, TAMRA-based Ub/Ubl FP assays, and FlAsH-based diUb cleavage assays are described in detail in the Supplemental Experimental Procedures.

### Protein Crystallization and Structure Determination

Crystallization conditions were optimized based on initial hits obtained from commercial screens in sitting-drop format. Structure determination was performed using either single wavelength anomalous dispersion (SAD) experimental phasing of selenomethionine derivatives or molecular replacement (see Table 1).

### Construction of CE Dendrogram

Novel and existing CE structures from bacteria, eukaryotes, and viruses were used to create a “seed” structure-based sequence alignment, which was iteratively refined and expanded on with additional examples. This alignment was used to identify more divergent examples of the CE fold and to construct a dendrogram of the CE clan using the UPGMA method.

### NMR Spectroscopy

NMR titration spectra of uniformly labeled ^15^N-Ub were recorded at 25°C on either a Bruker Avance III 600 MHz or an Avance2+ 700 MHz spectrometer, equipped with cryogenic triple-resonance TCI probes. Data processing and analysis were performed in Topspin (Bruker) and NMRView (One Moon Scientific).

### *S.* Typhimurium Infection Assays

Infection assays were performed as described previously (Rytkönen et al., 2007), with changes as discussed in the Supplemental Experimental Procedures.

## Author Contributions

D.K. conceived the project, and together, J.N.P. and D.K. designed the project. J.N.P. performed all of the biochemical and structural experiments, with input from D.K. B.S. performed the bioinformatics analysis. C.H.D. performed the *Salmonella* infection experiments, with input from D.W.H. P.P.G. and H.O. provided Ub/Ubl reagents. J.N.P. and D.K. wrote the manuscript, with input from all authors.

## Figures and Tables

**Table 1:** Data Collection and Refinement Statistics

	SseL 24-340 SeMet	SseL 24-340	ChlaDUB1 130-401 SeMet	ChlaDUB1 130-401	RickCE 420-691 SeMet	RickCE 420-691	XopD∼Ub 298-515	XopD∼tSUMO 298-515
**Data Collection**								

Beamline	ESRF ID23-1	ESRF ID23-1	DLS I03	DLS I03	DLS I04	DLS I04-1	DLS I04-1	DLS I04-1
Space group	*P* 3_1_ 1 2	*P* 3_1_ 1 2	*I* 2 3	*I* 2 3	*H* 3	*H* 3	*C* 2	*P* 6_4_
Cell dimensions *a*, *b*, *c* (Å)	113.30, 113.30, 166.62	113.01, 113.01, 166.60	133.45, 133.45, 133.45	133.29, 133.29, 133.29	178.33, 178.33, 56.81	173.50, 173.50, 55.52	117.75, 132.28, 117.31	119.10, 119.10, 50.46
α, β, γ (°)	90, 90, 120	90, 90, 120	90, 90, 90	90, 90, 90	90, 90, 120	90, 90, 120	90, 105.84, 90	90, 90, 120
Wavelength (Å)	0.9791	0.9791	0.9798	0.9763	0.9795	0.9174	0.9282	0.9282
Observed reflections	289,672	159,309	216,565	118,321	223,044	89,892	148,793	82,488
Unique reflections	15,721	33,454	12,344	23,093	8,501	24,203	37,920	23,605
Resolution (Å)	98.12–3.50 (3.83–3.50)	84.39–2.70 (2.83–2.70)	94.36–2.60 (2.72–2.60)	35.62–2.10 (2.16–2.10)	53.31–3.50 (3.83–3.50)	52.08–2.40 (2.49–2.40)	43.30–2.90 (3.03–2.90)	59.55–2.10 (2.16–2.10)
*R*_merge_	0.129 (0.319)	0.122 (0.627)	0.232 (1.530)	0.078 (0.521)	0.418 (2.726)	0.085 (0.596)	0.128 (0.568)	0.089 (0.465)
*I*/σ*I*	18.3 (10.9)	7.9 (2.4)	11.0 (2.2)	11.1 (2.8)	11.3 (3.6)	9.5 (2.0)	8.1 (2.4)	8.7 (2.0)
Completeness (%)	100.0 (100.0)	99.3 (99.8)	100.0 (100.0)	99.9 (99.8)	100.0 (100.0)	99.2 (99.7)	99.0 (99.1)	98.1 (99.4)
Redundancy	18.4 (17.4)	4.8 (4.9)	17.5 (18.0)	5.1 (5.1)	26.2 (26.2)	3.7 (3.8)	3.9 (4.0)	3.5 (3.4)

**Phasing**								

Method	SAD		SAD		SAD		MR (PDB: 2OIV and 1UBQ)	MR (PDB: 2OIV and 2IO0)
Resolution	3.5		2.6		4.0			
Anom completeness	100.0 (100.0)		100.0 (100.0)		100.0 (100.0)			
Anom multiplicity	9.5 (8.8)		8.9 (9.1)		12.8 (12.8)			
<FOM>	0.319		0.37		0.318			

**Refinement**								

Reflections in test set		1,693		1,112		1,190	1,835	1,281
*R*_work_/*R*_free_		18.7/22.8		18.3/20.8		16.4/21.7	24.0/28.6	18.1/21.5
Number of atoms								
Protein		4,868		2,135		4,025	8,176	2,173
Ligand/ion		3		6		0	16	26
Water		96		129		163	44	199
*B* factors								
Wilson *B*		45.6		34.5		36.8	43.2	24.8
Protein		53.3		38.4		42.1	46.3	29.7
Ligand/ion		59.1		50.0		–	44.1	50.7
Water		43.6		45.0		41.6	30.9	38.0
Rmsd								
Bond lengths (Å)		0.003		0.004		0.008	0.005	0.004
Bond angles (°)	0.67	0.82	1.10	0.82	0.58
Ramachandran statistics (favored/allowed/outliers)		96.2/3.8/0		97.4/2.6/0		97.7/2.1/0.2	97.5/2.4/0.1	97.4/2.6/0

Values in parentheses are for the highest resolution shell. Anom, anomalous; rmsd, root-mean-square deviations; FOM, figure of merit. See also Figures S2 and S3.

## Accession Numbers

The accession numbers for the coordinates and structure factors for SseL, ChlaDUB1, RickCE, XopD∼Ub, and XopD∼tSUMO reported in this paper have been uploaded to PDB: 5HAF, 5HAG, 5HAM, 5JP3, and 5JP1.

## Supplemental Information

Document S1. Supplemental Experimental Procedures and Figures S1–S7

Document S2. Article plus Supplemental Information
