# Supplementary material for: The Molecular Basis for Ubiquitin and Ubiquitin-like Specificities in Bacterial Effector Proteases
Source: Mol Cell. 2016 Jul 21;63(2):261–76. doi: 10.1016/j.molcel.2016.06.015 (PMC4961225; doi:10.1016/j.molcel.2016.06.015)
Supplement: Document S1. Supplemental Experimental Procedures and Figures S1–S7 [file mmc1.pdf]

Molecular Cell, Volume *63*

Supplemental Information

The Molecular Basis for Ubiquitin and Ubiquitin-like Specificities in Bacterial Effector Proteases

Jonathan N. Pruneda, Charlotte H. Durkin, Paul P. Geurink, Huib Ovaa, Balaji Santhanam, David W. Holden, and David Komander

Figure S1: CE effector proteins demonstrate mixed proteolytic activities

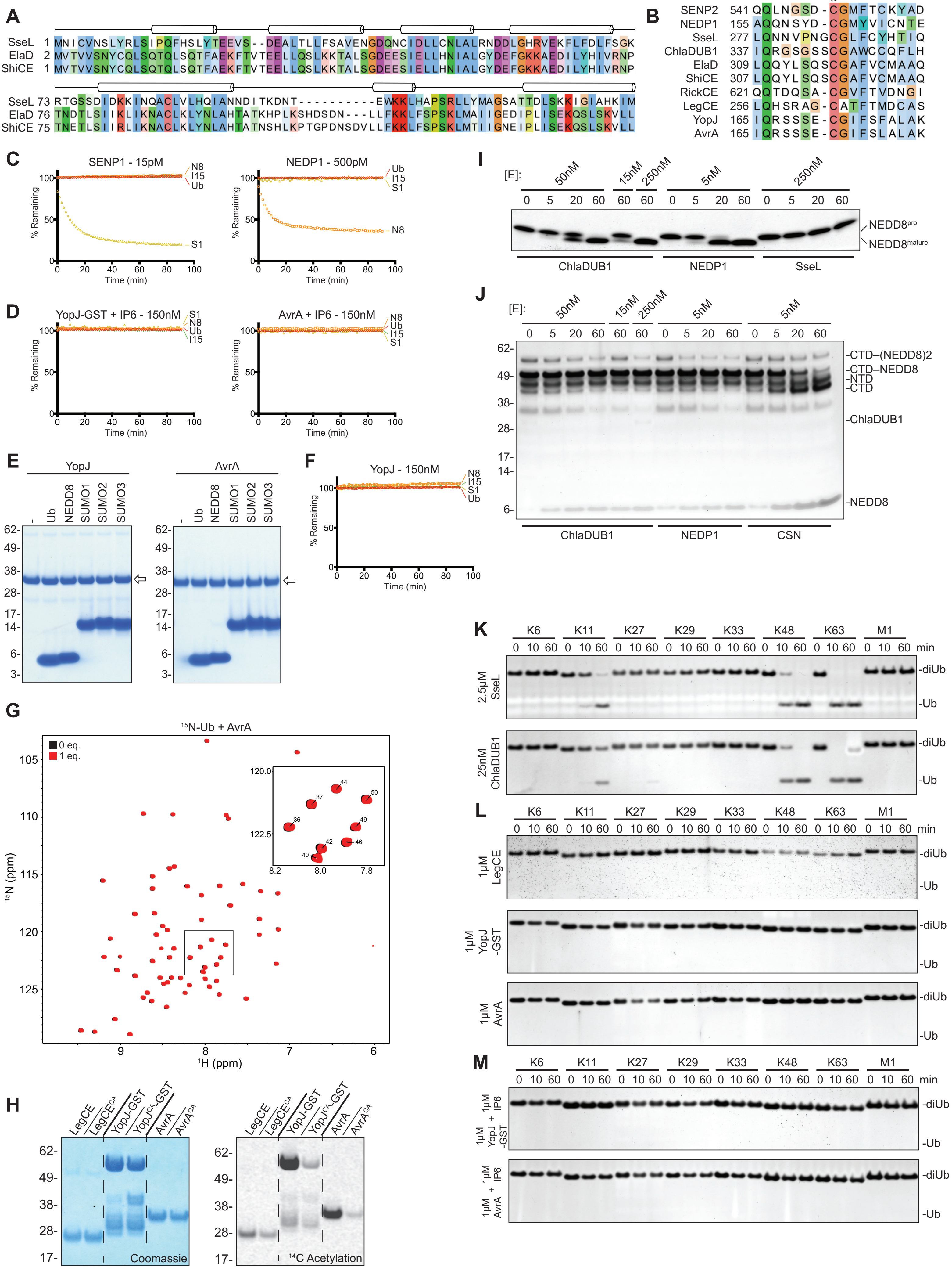

Figure S1: CE effector proteins demonstrate mixed proteolytic activities; related to Figure 1
A) Sequence alignment for the N-terminal accessory domains of SseL, ElaD, and ShiCE. Agreement in secondary structure prediction calculated for each individual sequence is shown above. **B)** Sequence alignment used to identify the putative catalytic Cys in bacterial CE enzymes (marked by an asterisk) by similarity to eukaryotic CE examples. **C)** Normalized FP measured as a function of time, as in **Figure 1E**, following addition of the well-studied SUMO-specific human SENP1 and NEDD8-specific human NEDP1. **D)** As in **C** for YopJ and AvrA in the presence of excess activator IP6. **E)** Suicide probe reaction, as in **Figure 1C**, monitoring reactivity of untagged YopJ and AvrA toward the indicated Ub/Ubl probes following 1 h incubation at room temperature (propargylamine-derived probes). **F)** As in **C** for untagged YopJ. **G)** $^{1}H,^{15}N$-HSQC TROSY spectra of 80μM ^{15}N-labeled Ub alone (black) and in the presence of 1 molar equivalent of AvrA (80μM, red). Inset, enlarged region of the spectral overlay with Ub resonance assignments labeled. **H)** Autoradiography monitoring isotope incorporation following a 2 h incubation of each protein with ^{14}C-labeled Acetyl-CoA in the presence of IP6. Compare signal in wild-type over inactive mutant background (see **Supplemental Experimental Procedures**). **I)** Time course monitoring cleavage of pro-NEDD8 (81 aa) to the mature form (76 aa), for the dual-specific ChlaDUB1, NEDD8-specific NEDP1, and Ub-specific SseL. This demonstrates that, like other CE proteases, ChlaDUB1 possesses both peptidase and isopeptidase activities. **J)** Time course monitoring cleavage of NEDDylated Cul1 C-terminal domain (CTD, modified at Lys720) in the presence of ChlaDUB1, and NEDP1 or COP9 signalosome (CSN) as negative and positive controls, respectively. Although ChlaDUB1 and NEDP1 can remove some NEDD8 modifications (e.g., CTD-(NEDD8)2), only the specialized CSN protease can remove the regulatory NEDD8 modification from Cul1 (CTD-NEDD8). **K)** Linkage specificity analysis for all diUb substrates, as in **Figure 1F**, for SseL and ChlaDUB1 at 10-fold higher enzyme concentration. **L)** Linkage specificity analysis for all diUb substrates, as in **Figure 1F**, for LegCE, YopJ, and AvrA. **M)** As in **L** for YopJ and AvrA in the presence of activator IP6.

Figure S2: Structural analysis of bacterial CE deubiquitinases

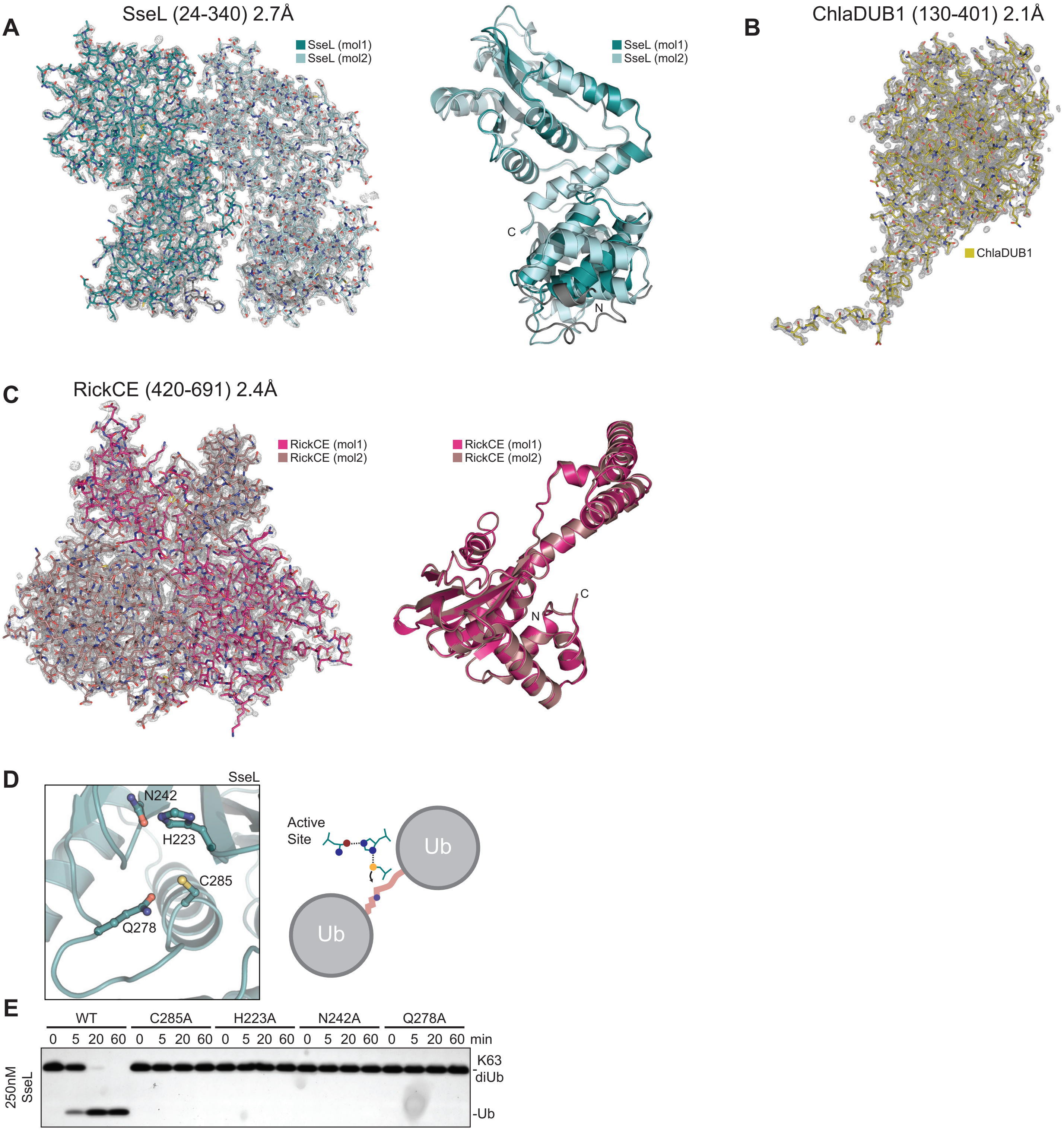

Figure S2: Structural analysis of bacterial CE deubiquitinases; related to Figure 2, Table 1

A) Full asymmetric unit (ASU) of the 2.7Å SseL (24-340) crystal structure showing a 2|Fo|-|Fc| electron density map contoured at 1σ, and superposition of the two copies in the ASU. Crystallization of this construct depended upon an N-terminal His-tag, which makes crystal contacts in one molecule and forms a short helix against the N-terminal domain in another. Most likely resulting from these crystal contacts at the N-terminus, the two molecules in the ASU show deviation within the N-terminal domain (1.99Å Cα RMSD for residues 24-136), but overlay well in the catalytic domain (0.39Å Cα RMSD for residues 154-340). SseL is colored in shades of teal, ordered regions of the His-tag are colored in grey. **B)** Full ASU of the 2.1Å ChlaDUB1 (130-401) structure showing 2|Fo|-|Fc| electron density contoured at 1σ. The first ~20 residues form a highly extended structure via crystal contacts. **C)** Full ASU of the 2.4Å RickCE (420-691) crystal structure, along with a 2|Fo|-|Fc| electron density map contoured at 1σ, and an alignment of the two copies in the ASU. The two identical molecules in the ASU (0.27Å Cα RMSD) are interlaced by a large insertion forming a helical arm. **D)** Active site Cys, His, Asn, and Gln of SseL (teal) are shown. **E)** K63 diUb cleavage assay using wild-type and catalytic mutants of SseL.

Figure S3: Molecular analysis of XopD Ub/Ubl specificity

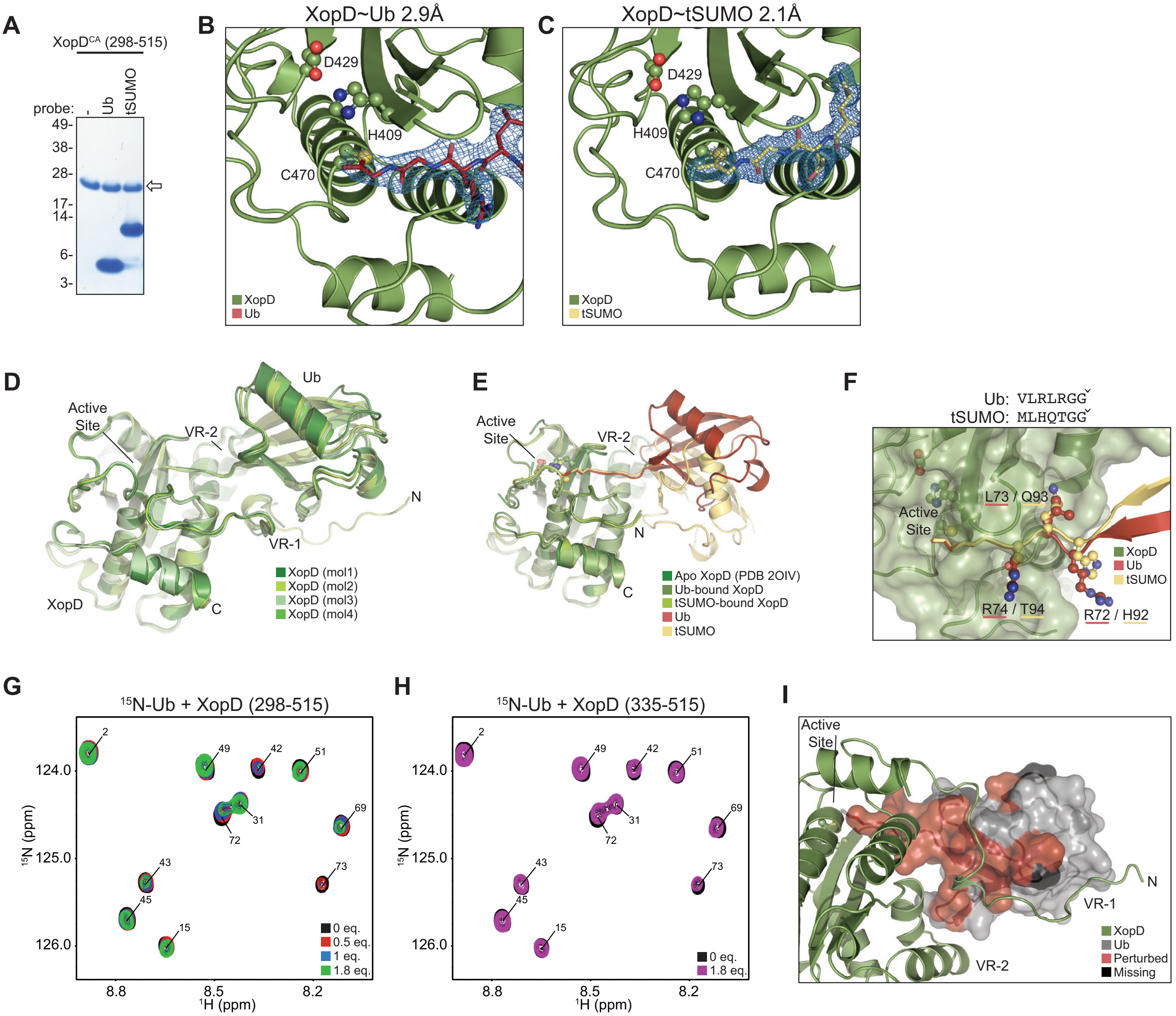

Figure S3: Molecular analysis of XopD Ub/Ubl specificity; related to Figure 3, Table 1
A) Catalytically inactive XopD +VR-1 C470A tested against the Ub and tSUMO suicide probes following a 1 h incubation at room temperature (propargylamine-derived probes). Open arrow, unmodified. **B)** Close-up of the XopD~Ub covalent linkage, showing XopD catalytic triad residues and 2|Fo|-|Fc| electron density of the Ub C-terminus, contoured at 1σ. **C)** As in **B**, for the XopD~tSUMO complex. **D)** Superposition of the four XopD~Ub molecules present in the ASU. **E)** Superposition of XopD from its apo (PDB 2OIV), Ub-bound, and tSUMO-bound structures. VR-1 is not shown for clarity. **F)** Close-up of the tract leading into the XopD active site. C-terminal residues for Ub and tSUMO are shown above, and their positions within the XopD-bound structures is depicted below. **G)** $^{1}H,^{15}N$-HSQC TROSY spectra of 80μM ^{15}N-labeled Ub alone (black) and in the presence of 0.5 (40μM, red), 1 (80μM, blue), or 1.8 (144μM, green) molar equivalents of XopD +VR-1 (298-515). **H)** As in **G**, for 1.8 (144μM, magenta) molar equivalents of XopD ΔVR-1 (335-515). **I)** Resonances significantly perturbed in **G** by either line broadening or chemical shift perturbation mapped onto the XopD~Ub crystal structure (red). Prolines and other resonances missing from the NMR spectrum are shown in black.

Figure S4: Structure-based manipulation of CE DUB activities and specificities

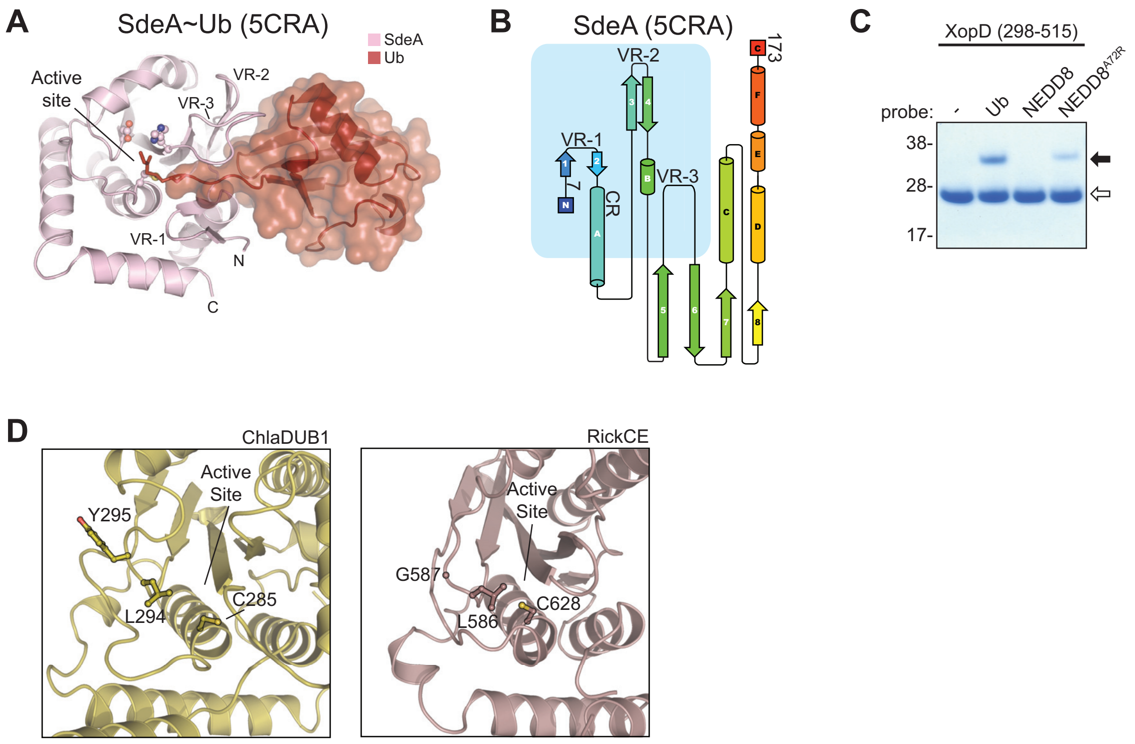

Figure S4: Structure-based manipulation of CE DUB activities and specificities; related to Figure 4
A) Cartoon representation of SidE family member SdeA in a covalent complex with Ub (PDB 5CRA). **B)** Topology diagram, as in **Figure 2E**, for the SdeA crystal structure (PDB 5CRA). **C)** Suicide probe assay showing XopD +VR-1 (298-335) reaction with Ub, NEDD8, and NEDD8 A72R probes at room temperature for 1 h (chloroethylamine-derived probes) Open arrow, unmodified; closed arrow, modified. **D)** Close-up of the conserved hydrophobic S1' sites of ChlaDUB1 and RickCE.

Figure S5: Bioinformatic analysis of the CE protease clan

A

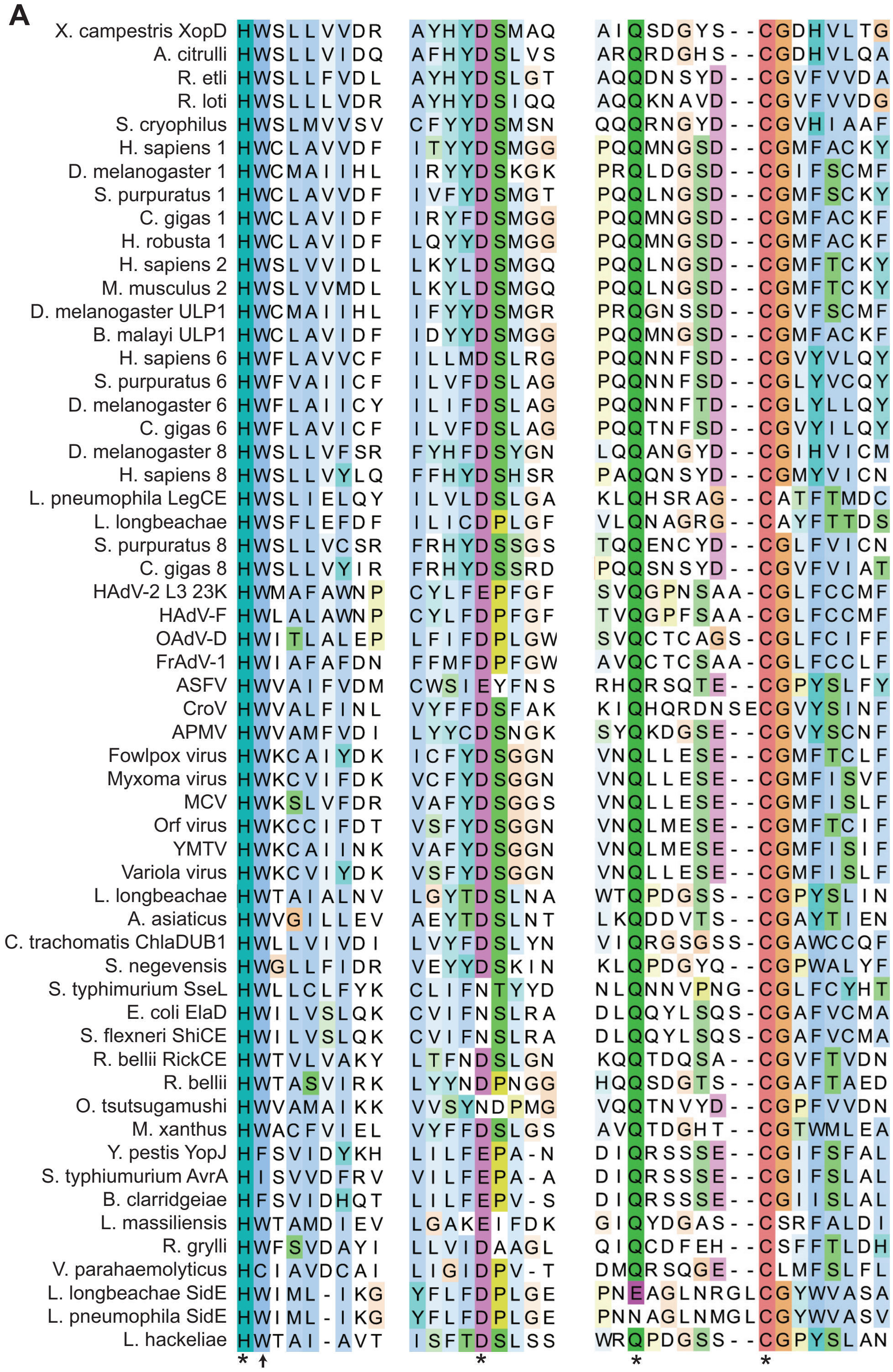

Figure S5: Bioinformatic analysis of the CE protease clan; related to Figure 5

A) Structure-guided sequence alignment of representative members from the CE clan, used to construct the dendrogram shown in **Figure 5**. For clarity, only regions immediately surrounding the catalytic center are shown. Coloring is based on similarity. One conserved Trp that is typically lacking in dedicated acetyltransferases is marked with an arrow. Catalytic residues are marked below with an asterisk.

Figure S6: CE catalytic domains are fitted with accessory domains

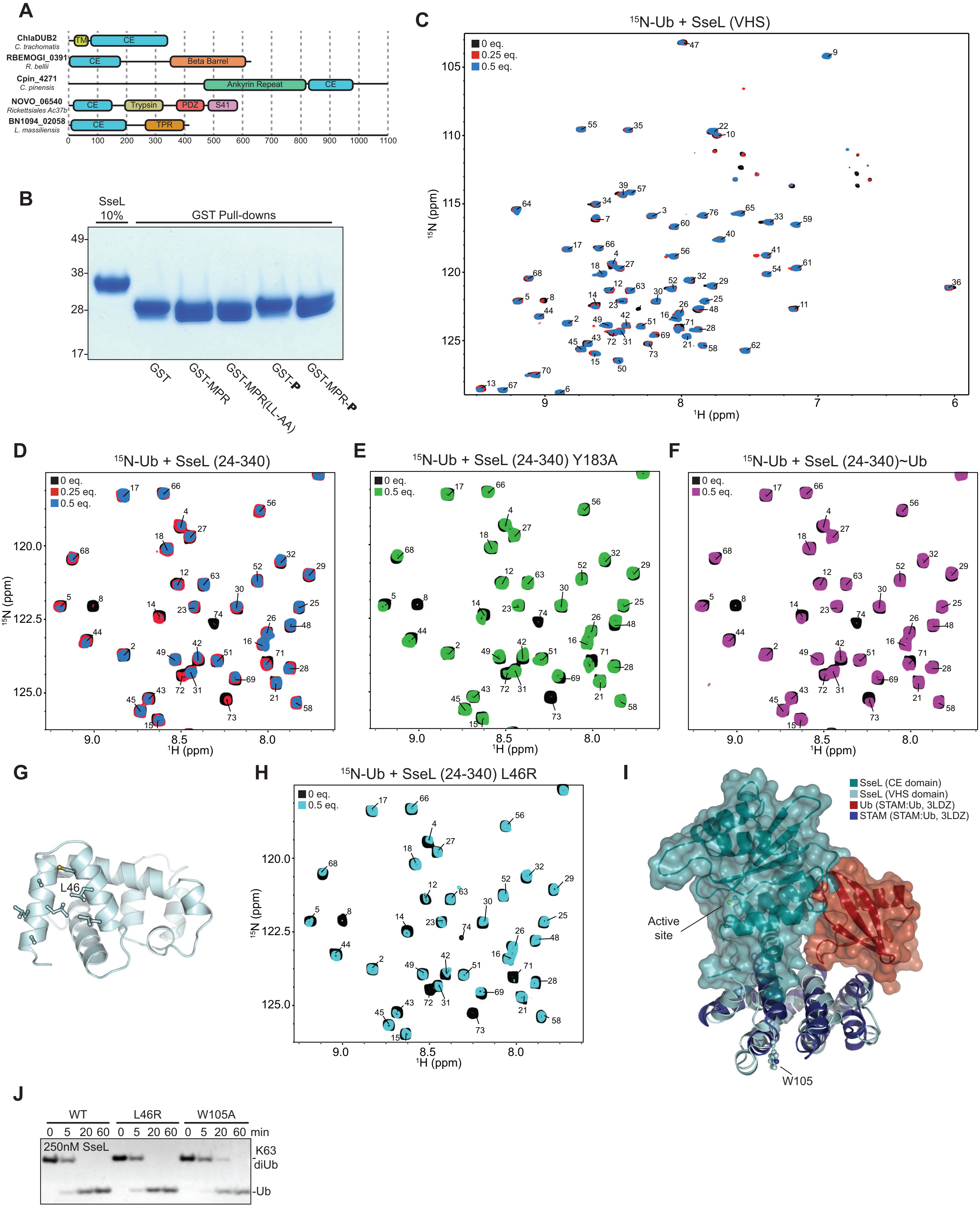

Figure S6: CE catalytic domains are fitted with accessory domains; related to Figure 6
A) Domain annotation of select CE enzymes from bacteria. TM, transmembrane helix; PDZ, PDZ protein interaction domain; S41, S41 peptidase domain; TPR, tetratricopeptide repeat. **B)** GST pull-down assay testing interaction of SseL to the cation independent mannose-6-phosphate receptor (CI-MPR) C-terminal peptide, as well as its LL-AA double mutant form and CK2-phosphorylated form. **C)** Full spectrum for the NMR titration shown in **Figure 6C**. 80 μM ^{15}N-labeled Ub alone (black) and in the presence of 0.25 (20μM, red) or 0.5 (40μM, blue) molar equivalents of SseL VHS domain. Line broadening effects suggest an interaction in the low-to-mid micromolar range. **D)** ^{1}H,^{15}N-HSQC TROSY titration experiment showing 80 μM ^{15}N-labeled Ub alone (black) and in the presence of 0.25 (20 μM, red) or 0.5 (40 μM, blue) molar equivalents of SseL (24-340). **E)** As in **C**, showing 0.5 molar addition of the S1 site Y183A mutant (green). **F)** As in **C**, showing 0.5 molar addition of the covalently-linked SseL~Ub complex (magenta). **G)** Exposed hydrophobic patch within the SseL VHS domain; Leu46 was chosen for mutation. **H)** ^{1}H,^{15}N-HSQC TROSY spectrum of 80 μM ^{15}N-labeled Ub alone (black) and in the presence of 0.5 molar equivalents of SseL (24-340) L46R (40μM, cyan). **I)** Overlay of the SseL catalytic and VHS domains (teal and cyan, respectively) with the STAM:Ub crystal structure (PDB 3LDZ, blue and red, respectively). As a conventional VHS:Ub interaction would clash with the position of the SseL catalytic domain, an unconventional site centered around Trp105 is utilized. **J)** Time course assays monitoring cleavage of K63-linked diUb with the SseL VHS domain mutants.

Figure S7: SseL accessory VHS domain dictates subcellular localization

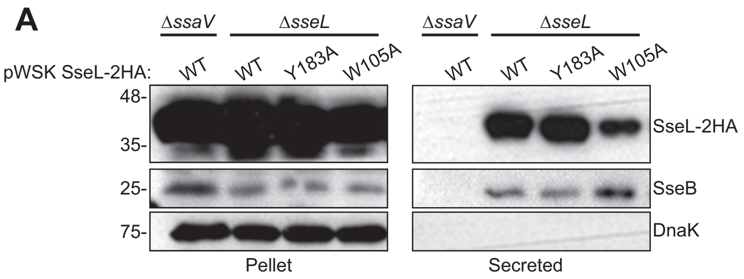

Figure S7: SseL accessory VHS domain dictates subcellular localization; related to Figure 7
A) Induced SPI-2 T3SS secretion of cultured *S.* Typhimurium strains expressing 2HA-tagged wild-type or mutant SseL, in the Δ*sseL* or Δ*ssaV* (SPI-2 T3SS null) background. Secreted and pellet fractions were separated by SDS-PAGE and immunoblotted for HA (SseL), SseB (SPI-2 T3SS translocon protein), and DnaK (intracellular control protein).

Supplemental Experimental Procedures:

Cloning and molecular biology

SseL, ElaD, and LegCE were cloned from the genomic DNA of *Salmonella* Typhimurium (strain LT2), *Escherichia coli* (serotype O157:H7), and *Legionella pneumophila* (strain Philadelphia, kind gift from C. Buchrieser, Institut Pasteur) using Phusion DNA polymerase (NEB). Genes encoding *Chlamydia trachomatis* ChlaDUB1, *Shigella flexneri* ShiCE, *Rickettsia bellii* RickCE, and *Xanthomonas campestris* XopD were obtained by gene synthesis (Life Technologies) and subcloned using KOD polymerase (Novagen). Amplified products were cloned into pOPIN vectors (Berrow et al., 2007) using the In-Fusion HD system (Clontech). Mutagenesis was performed using the QuikChange protocol. Constructs for YopJ in pMW and AvrA in pGEX6P-1 were kind gifts from R. Mittal (MRC LMB Cambridge). Constructs for SENP1 and NEDP1 in pHISTEV were kinds gifts from R. Hay (University of Dundee). tSUMO was amplified from a *Solanum lycopersicum* cDNA preparation (kind gift from A. Canto-Pastor and D. Baulcombe, University of Cambridge) and ligated into pTXB1 with conventional methods.

Protein expression and purification

SseL (24-340), ChlaDUB1 (130-401), ElaD (2-407), ShiCE (2-405), RickCE (378-691 or 420-691), LegCE (141-360), and XopD (298-515 or 335-515) were expressed from the pOPIN-B vector (N-terminal His-3C), AvrA (1-288) from the pGEX6P-1 vector (N-terminal GST-3C), YopJ (1-288) from the pMW vector (C-terminal 3C-GST), and SENP1 (415-643), NEDP1 (2-212) from the pHISTEV vector (N-terminal His-TEV). The SseL 24-340 construct may represent the full-length sequence secreted by *S.* Typhimurium, depending on usage of an alternative GTG start codon at position 24. The SseL VHS domain construct, cloned into pOPIN-B, included residues 24-137 as well as an 'EQVGVENLWRD' C-terminal sequence designed using the crystal structure to reconstitute key stabilizing interactions made by the C-terminal catalytic domain. With the exception of YopJ, SENP1, NEDP1, and SseL VHS domain, all tags were cleaved as part of the purification.
All constructs were transformed into the *E. coli* Rosetta2 pLacI strain. 2-12 L cultures were grown at 37˚C in 2xTY medium to an OD_{600} of 0.8-1.0, at which time protein expression was induced with 0.2 mM IPTG for 16-20 h at 18˚C. Cells were resuspended in 25 mM Tris (pH 7.4), 200 mM NaCl, 2 mM ß-mercaptoethanol (Buffer A). Following freeze-thaw and addition of EDTA-free Complete protease inhibitor tablets (Roche), DNase, and Lysozyme, cells were lysed by sonication or using an Emulsiflex C3. Lysates were clarified by centrifugation at 35000 x g for 25 min, and applied to Talon (Clontech) or Glutathione Sepharose 4B (GE Healthcare) resin for gravity flow purification according to manufacturer recommendations. His-tagged proteins were washed thoroughly with Buffer A, eluted in Buffer A with 250 mM imidazole, and (with the exception of SENP1 and NEDP1) the tag was cleaved overnight with His-3C protease during dialysis back to Buffer A. Cleaved protein was reapplied to Talon resin and flow-through was collected for further purification. GST-tagged proteins were washed thoroughly with Buffer B (25 mM Tris (pH 8.5), 5 mM DTT)) plus 500 mM NaCl, then Buffer B plus 50 mM NaCl, prior to elution with 10 mM GSH (in the case of YopJ) or overnight cleavage with addition of GST-3C protease. When required, cleaved proteins were exchanged into Buffer B plus 50 mM NaCl and applied to anion exchange chromatography (ResourceQ 6 mL, GE Healthcare), followed by elution with a 50-500 mM NaCl gradient. The SseL VHS domain was purified from inclusion bodies. Following solubilization in Buffer A plus 8 M Urea, 10 mM NH_4Cl, SseL VHS domain was bound to gravity flow Ni^{2+} resin (Qiagen), refolded using a gradient to Buffer A without Urea, and eluted in Buffer A plus 250 mM imidazole. All proteins were subjected to final gel filtration (Superdex75, GE Healthcare) equilibrated in 25 mM HEPES (pH 8.0), 150 mM NaCl, 5 mM DTT. Protein-containing fractions were concentrated, aliquoted, and flash-frozen prior to storage at -80˚C.

TAMRA-based cleavage assays

All Ub/Ubl-TAMRA-KG reagents were prepared as previously described (Geurink et al., 2012; Basters et al., 2014), with the exception of the tSUMO-TAMRA-KG reagent, which was prepared as follows:

Native chemical ligation

tSUMO-MesNa (aa 1-96, 4.0 mg, 0.37 μmol), prepared as previously described (Borodovsky et al., 2002), and TMR-Lys(thio)G (4.0 mg, 5.4 μmol) were dissolved in 200 μL of aqueous buffer containing 6.0 M Gn·HCl, 0.15 M Na_2HPO_4 and 0.25 M MPAA at pH 7.2 and shaken overnight at 37°C. The product was purified by RP-HPLC (Shimadzu, C18, 10-70% v/v ACN/H_2O gradient, 0.05% v/v TFA). The appropriate fractions were pooled and lyophilized.

Desulfurization

The resulting product (ca. 4 mg) was dissolved in aqueous buffer containing 6.0 M Gn·HCl, 0.15 M Na_2HPO_4 and 0.25 M TCEP at pH 7.0 to a concentration of 1 mg/mL protein. Reduced glutathione (GSH) was added to the solution to a concentration of 100 mM. The pH of the solution was adjusted to pH 7.0 by addition of 1 M NaOH. VA-044 was added to the solution to a final concentration of 75 mM. The reaction mixture was flushed with argon and shaken for 4 h at 37°C after which LC-MS analysis indicated a complete reaction. The product

was purified by RP-HPLC (Shimadzu, C18, 10-70% v/v ACN/H_2O gradient, 0.05% v/v TFA). The appropriate fractions were combined, lyophilized, dissolved in H_2O/ACN/formic acid (65/35/10; v/v/v; 10 mL) and lyophilized again. Finally, the product was purified on a Superdex75 size exclusion column (GE Healthcare) equilibrated in 50 mM Tris (pH 7.6), 100 mM NaCl. This yielded 0.85 mg (75 nmol, 21%) of the tSUMO-TAMRA-KG substrate.

Fluorescence polarization (FP) assays monitoring cleavage of the Ub/Ubl substrates were performed as described previously (Geurink et al., 2012). Briefly, enzymes and Ub/Ubl substrates were diluted in 25 mM Tris (pH 7.4), 100 mM NaCl, 5 mM ß-mercaptoethanol, 0.1 mg/mL BSA (FP buffer). Enzyme concentrations were varied to capture a representative activity profile, up to a maximum concentration of 150 nM. Substrate concentrations were held at 150 nM. Enzyme and substrate were mixed in black 384-well plates (Corning) immediately before the assay began. FP was measured in a Pherastar plate reader (BMG Labtech) equipped for 550 nm excitation and 590 nm emission. Cleavage measurements were normalized to independently measured negative (Ub/Ubl substrate only) and positive (25 nM TAMRA-KG peptide) control samples. All comparative data (Ub vs. Ubl, wild-type vs. mutant) are presented from one representative replicate.

Qualitative cleavage assays
Deubiquitination specificity assays were performed as described previously (Licchesi et al., 2012). Enzymes were diluted to a 2x stock concentration in 25 mM Tris (pH 7.4), 150 mM NaCl, 10 mM DTT and combined 1:1 with 6 µM diUb prepared in 100 mM Tris (pH 7.4), 100 mM NaCl, 10 mM DTT. Reactions were incubated at 37˚C, time points were resolved by SDS-PAGE and silver stained (BioRad).
Pro-NEDD8 (Boston Biochem) and Cul1-NEDD8 (kind gift from B. Schulman, St. Jude Children's Research Hospital) cleavage assays were performed with the same protocol.

Acetylation assays
Enzymes were diluted to 5 µM in 25 mM HEPES (pH 8.0), 50 mM NaCl, 200 nM inositol hexakisphosphate (IP6), 0.5 mM DTT and incubated with 60 µM [1-^{14}C] AcCoA (60 mCi/mmol, PerkinElmer) at 37˚C for 2 h. Reactions were resolved by SDS-PAGE. The gel was dried and exposed to a Phosphor screen, which was then imaged on a Typhoon scanner (GE Healthcare). In our assays, catalytically inactive mutants, as well as unrelated protein standards (not shown), can be non-enzymatically modified by AcCoA. Therefore, only an increased signal in wild-type assays compared to the catalytically inactive controls should be considered true acetylation activity.

Suicide probe assays
Ub- and Ubl-PA probes were generated as described previously (Ekkebus et al., 2013), with exception of tSUMO-PA and all Ub/tSUMO-PA point mutants, which were prepared according to (Borodovsky et al., 2002) with propargylamine. Assays testing Ub/NEDD8 specificity were performed using suicide probes prepared from 2-chloroethylamine, as described previously (Borodovsky et al., 2002). Enzymes were diluted to 10 µM in 25 mM Tris (pH 7.4), 150 mM NaCl, 10 mM DTT, and combined 1:1 with 100 µM probe. Reactions were incubated at room temperature or on ice (as specified), time points were resolved by SDS-PAGE and Coomassie stained.

Protein crystallization
Initial hits were obtained using commercial screens in the sitting-drop vapor diffusion format.
Native and SeMet His-SseL (24-340) were prepared in 25 mM Tris (pH 7.4), 200 mM NaCl, 2 mM ß-mercaptoethanol and crystallized at 14 mg/mL in 0.1 M Tris, 1.6 M K_2HPO_4 (final pH 8.7), with a 1 µL sitting drop at 2:1 protein:precipitant ratio. SseL crystals were cryoprotected in ParatoneN (native crystals) or paraffin oil (SeMet crystals).
Native ChlaDUB1 (130-401) was prepared in 25 mM Tris (pH 7.4), 125 mM NaCl, 4 mM DTT and crystallized at 12 mg/mL in 0.1 M HEPES (pH 7.5), 20% PEG 8000, with a 400 nL sitting drop at 1:1 protein:precipitant ratio. SeMet ChlaDUB1 was crystallized at 8 mg/mL in a 1 µL sitting drop of the matched condition, using seeds of native crystals. Crystals were cryoprotected using mother liquor containing 22-25% glycerol.
Native RickCE (420-691) was prepared in 25 mM Tris (pH 7.4), 125 mM NaCl, 4 mM DTT and crystallized by hanging drop, mixing 2 µL of 13 mg/mL protein with 1 µL of 0.1 M Tris (pH 7.5), 2 M ammonium citrate. SeMet RickCE was crystallized the same manner, using native RickCE crystals as seeds. Crystals either had no cryoprotectant (SeMet RickCE), or were cryoprotected with mother liquor containing 22-25% glycerol.
The XopD~Ub complex was prepared in 25 mM Tris (pH 7.4), 125 mM NaCl, 4 mM DTT and crystallized at 12 mg/mL in 0.1 M CHES (pH 9.5), 1.0 M sodium citrate, with a 200 nL sitting drop at 1:1 protein:precipitant ratio. Crystals were cryocooled with no protectant. The XopD~tSUMO complex was prepared similarly, and

crystallized at 8 mg/mL in 0.1 M bicine (pH 9.0), 1.6 M ammonium sulfate. XopD~tSUMO crystals were cryoprotected in 3.4 M malonate.

Data collection, structure determination, and refinement
Diffraction data were collected at the European Synchrotron Radiation Facility (ESRF) beam line ID23-1, and the Diamond Light Source (DLS) beam lines I03, I04, and I04-1 (see **Table 1**). Images were integrated using either MOSFLM (Battye et al., 2011) or XDS (Kabsch, 2010) and scaled using Aimless (Evans and Murshudov, 2013). Structures were solved experimentally using SeMet SAD datasets in PHENIX AutoSol and AutoBuild (Adams et al., 2010; Terwilliger et al., 2009; Terwilliger et al., 2008) (**Table 1**). XopD~Ub and XopD~tSUMO were solved using molecular replacement in Phaser (McCoy et al., 2007) using the apo XopD structure (PDB 2OIV) and Ub (PDB 1UBQ) or SUMO1 structures (PDB 1TGZ). Model building and refinement were performed using COOT (Emsley et al., 2010) and PHENIX (Adams et al., 2010). The XopD~Ub structure was refined with the higher resolution XopD structure (XopD~tSUMO) and Ub (PDB 1UBQ) as reference models. See **Table 1** for final statistics.
All figures were generated using PyMOL (www.pymol.org). Secondary structure topology diagrams are based on output from the Pro-origami server (Stivala et al., 2011).

Plant extract protease assays
S. lycopersicum leaves (kind gift from A. Canto-Pastor and D. Baulcombe, University of Cambridge) were flash frozen in liquid nitrogen and ground by mortar and pestle in a lysis buffer containing 50 mM HEPES (pH 8.0), 150 mM NaCl, 25 mM sucrose, 5 mM DTT, 1 mM EDTA, and 1 mM PMSF. Insoluble material was removed following 16000 x g centrifugation at 4˚C for 10 min. The clarified lysate was incubated with recombinant XopD constructs at a 1 μM final concentration for 1 h at room temperature. Thirty micrograms of the resulting samples were separated by SDS-PAGE, transferred to nitrocellulose, and blotted for total ubiquitin (Ubi-1, Novus Biologicals) following Ponceau staining of total protein.

Quantitative cleavage assays
FlAsH-tagged diUb substrates were assembled enzymatically (UBE2N/UBE2V1 for K63 chains, UBE2R1 for K48 chains) by ligating a C-terminally FlAsH tagged Ub to 'blocked' Ub mutants (K63R for K63 chains, K48R for K48 chains). Unlabeled diUb chains were assembled analogously, replacing FlAsH-tagged Ub with a construct lacking its C-terminus (Ub aa 1-72). FlAsH-labeled diUb was held at a constant concentration of 75nM in all assays, and the remaining substrate consisted of unlabeled diUb. Assays were performed in 20 μL using black 384-well plates (Corning). FP was measured in a Pherastar plate reader (BMG Labtech) equipped for 485 nm excitation and 520 nm emission. SseL wild-type was used at 100 nM and 10 μM final concentration for K63 and K48 measurements, respectively. SseL Y244A was used at 1 μM final concentration in all assays. For each substrate concentration, initial rates, measured in triplicate, were subtracted from a substrate-only control and converted into velocities of substrate conversion. Kinetic analysis was performed using Graphpad Prism 6.

Construction of CE dendrogram
Given the significant sequence and structural diversities within the CE clan, we adopted a multipronged approach to build an accurate sequence alignment as described below. 1) Bonafide members of the CE superfamily of proteases were obtained from MEROPS, Pfam, and Interpro databases (Rawlings et al., 2014; Finn et al., 2014; Mitchell et al., 2015). 2) A "seed" structure-based sequence alignment was constructed based on available structures of CE superfamily domains such as SENP1, SENP2, NEDP1, L3 23K of human adenovirus 2 and XopD (PDB ID: 2XPH, 3ZO5, 2BKR, 1AVP, and 2OIX, respectively) (Rimsa et al., 2011; Alegre and Reverter, 2014; Shen et al., 2005; Ding et al., 1996; Chosed et al., 2007) and the presented structures of SseL, ChlaDUB1, and RickCE. The alignment was constructed using MUSTANG (Konagurthu et al., 2006) and DaliLite (Holm and Park, 2000) programs and was further manually refined based on residue conservation patterns and assignment of secondary structures from the above PDB structures. 3) The alignment was then expanded to include sequence data from other families within the CE clan such as YopJ and the Poxviridae proteases. We also included more sequence data from the families already represented in the seed alignment. An initial version of the expanded alignment was constructed using MUSCLE program (Edgar, 2004) with the option of profile-to-sequence alignment, which does not perturb the original seed alignment. This expanded alignment was further manually refined based on residue conservation patterns and secondary structure prediction by JPRED (Drozdetskiy et al., 2015) to obtain a final expanded alignment. 4) Once an accurate version of the expanded alignment was constructed, we performed database searches using HMMER, PSI-BLAST and HHpred (Finn et al., 2011; Altschul et al., 1997; Söding et al., 2005) to find more divergent homologs of known CE superfamily members. This process identified e.g. the SidE family as part of the CE clan. Representative members of SidE family were then included in the expanded alignment. 5) MEGA software (Tamura et al., 2007) was used to construct a dendrogram based on the expanded alignment of the CE clan using

the UPGMA method with a bootstrap procedure for 1000 trials. A maximum likelihood method was also employed, but did not reliably produce dendrograms with cohesive functional relationships, likely due to high levels of dissimilarity among branchpoints.

GST pull-down assays
GST-tagged CI-MPR peptide was prepared by ligating sequence for the 'FHDDSDEDLLHI' peptide (CI-MPR aa 2480-2491) into the pOPIN-K vector, which encodes an N-terminal GST-3C tag. The wild-type sequence, as well as the 'LL-AA' double mutant were expressed and purified alongside the empty pOPIN-K vector, which contains the 'GTVDPTGKRAVSATQLM' sequence at its C-terminus as a control. 50 µg of GST or GST-MPR were phosphorylated by incubation with 250 units of CK2, 5 mM $MgCl_2$, and 1 mM ATP at 30˚C for 30 min. For the pull-down assay, 50 µg of each GST-tagged protein was bound to 25 µL of Glutathione Sepharose 4B resin equilibrated in 20 mM Tris (pH 7.4), 100 mM NaCl, 5 mM ß-mercaptoethanol, and washed 3x with 250 µL additional cold buffer. 50 µg of SseL was added to a final concentration of 25 µM, and allowed to bind on ice for 30 min. Samples were washed 7x with 250 µL of cold buffer, and eluted with 25 µL buffer containing 10 mM GSH. Samples of SseL loading and GST elutions were resolved by SDS-PAGE and visualized by Coomassie staining.

NMR spectroscopy
Uniformly labeled ^{15}N-Ub was expressed in M9 minimal media supplemented with ^{15}N-NH_4Cl, and purified as described previously (Pickart and Raasi, 2005). All proteins were exchanged into 25 mM sodium phosphate (pH 7.0), 150 mM NaCl, 1 mM DTT. NMR spectra were recorded at 298K on either a Bruker Avance III 600 MHz or Avance2+ 700 MHz spectrometer, equipped with cryogenic triple resonance TCI probes. Data processing and analysis were performed in Topspin (Bruker) and NMRView (One Moon Scientific).

S. Typhimurium infection assays
The *S.* Typhimurium Δ*sseL* strain was transformed with pWSK29 plasmids containing SseL-2HA mutants (see table of strains below). SPI-2 dependent secretion of SseL was tested using the pH shift secretion assay (Yu et al., 2010). Briefly, overnight cultures were diluted 1:50 into Mg-MES (pH 5) and grown for 4 hours, then transferred to Mg-MES (pH 7.2) for 90 minutes. Secreted protein and bacterial cells were collected and resolved by SDS-PAGE and immunoblotted with DnaK (*Salmonella* intracellular chaperone, Enzo ADI-SPA-880-D), SseB (SPI-2 T3SS translocon protein, generated in Beuzón et al., 2002) and HA (SseL, HA.11 Cambridge Biosciences) antibodies.

HeLa cells were infected for 16 hours at a multiplicity of infection (MOI) of 200 with late exponential phase *S.* Typhimurium strains. Cells were collected in SDS sample buffer for total cell lysate or collected into Phosphate-Buffered Saline (PBS), pelleted at 300 x g and lysed in PBS + 0.1% triton. The lysate was clarified at 3,000 x g and the supernatant collected as the cytosolic fraction. Samples were resolved by SDS-PAGE and immunoblotted with HA.11 (SseL), DnaK (*S.* Typhimurium intracellular chaperone), or Actin antibodies. HeLa cells seeded on coverslips were infected as above and fixed with 3% Paraformaldehyde at 16 hpi. 0.1% saponin was used to permeabilize HeLa cells membrane but not bacterial membranes. Translocated SseL and *S.* Typhimurium were detected using HA (3F10 Roche) and CSA-1 (Insight Biotechnology) antibodies, respectively.

Strains used in this study:

Strain	Description	Source
ΔsseL	ΔsseL:Km in 12023	(Rytkönen et al., 2007)
ΔsseL pSseL 2HA	pWSK29*sseL*-2HA in ΔsseL	(Rytkönen et al., 2007)
ΔssaV pSseL 2HA	pWSK29*sseL*-2HA in ΔssaV	(Rytkönen et al., 2007)
ΔsseL pSseL 2HA Y183A	pWSK29*sseLY183A* -2HA in ΔsseL	This study
ΔsseL pSseL 2HA W105A	pWSK29*sseLW105A*-2HA in ΔsseL	This study

Supplemental References:

Adams, P.D., Afonine, P.V., Bunkoczi, G., Chen, V.B., Davis, I.W., Echols, N., Headd, J.J., Hung, L.W., Kapral, G.J., Grosse-Kunstleve, R.W., McCoy, A.J., Moriarty, N.W., Oeffner, R., Read, R.J., Richardson, D.C., Richardson, J.S., Terwilliger, T.C., and Zwart, P.H. (2010). PHENIX: a comprehensive Python-based system for macromolecular structure solution. Acta Crytallogr. D Biol. Crystallogr. *66*, 213-221.

Altschul, S.F., Madden, T.L., Schäffer, A.A., Zhang, J., Zhang, Z., Miller, W., and Lipman, D.J. (1997). Gapped BLAST and PSI-BLAST: a new generation of protein database search programs. Nucl. Acids Res. *25*, 3389-3402.

Battye, T.G.G., Kontogiannis, L., Johnson, O., Powel, H.R., and Leslie, A.G.W. (2011). iMOSFLM: a new graphical interface for diffraction-image processing with MOSFLM. Acta Crytallogr. D Biol. Crystallogr. *67*, 271-281.

Berrow, N.S., Alderton, D., Sainsbury, S., Nettleship, J., Assenberg, R., Rahman, N., Stuart, D.I., and Owens, R.J. (2007). A versatile ligation-independent cloning method suitable for high-throughput expression screening applications. Nucl. Acids Res. *35*, e45.

Beuzón, C.R., Banks, G., Deiwick, J., Hensel, M., and Holden, D.W. (2002). pH-dependent secretion of SseB, a product of the SPI-2 type III secretion system of *Salmonella typhimurium*. Mol. Microbiol. *33*, 806-816.

Borodovsky, A., Ovaa, H., Kolli, N., Gan-Erdene, T., Wilkinson, K.D., Ploegh, H.L., and Kessler, B.M. (2002). Chemistry-based functional proteomics reveals novel members of the deubiquitinating enzyme family. Chem. Biol. *9*, 1149-1159.

Drozdetskiy, A., Cole, C., Procter, J., and Barton, G.J. (2015). JPred4: a protein secondary structure prediction server. Nucl. Acids Res. *43*, W389-W394.

Edgar, R.C. (2004). MUSCLE: multiple sequence alignment with high accuracy and high throughput. Nucl. Acids Res. *32*, 1792-1797.

Emsley, P., Lohkamp, B., Scott, W.G., and Cowtan, K. (2010). Features and development of Coot. Acta Crytallogr. D Biol. Crystallogr. *66*, 486-501.

Evans, P.R. and Murshudov, G.N. (2013). How good are my data and what is the resolution? Acta Crytallogr. D Biol. Crystallogr. *69*, 1204-1214.

Finn, R.D., Clements, J., and Eddy, S.R. (2011). HMMER web server: interactive sequence similarity searching. Nucl. Acids Res. *39*, W29-W37.

Finn, R.D., Bateman, A., Clements, J., Coggill, P., Eberhardt, R.Y., Eddy, S.R., Heger, A., Hetherington, K., Holm, L., Mistry, J., Sonnhammer, E.L.L., Tate, J., and Punta, M. (2014). The Pfam protein families database. Nucl. Acids Res. *42*, D222-D230.

Holm, L. and Park, J. (2000). DaliLite workbench for protein structure comparison. Bioinformatics. *6*, 566-567.

Kabsch, W. (2010). XDS. Acta Crytallogr. D Biol. Crystallogr. *66*, 125-132.

Konagurthu, A.S., Whisstock, J.C., Stuckey, P.J., and Lesk, A.M. (2006). MUSTANG: a multiple structural alignment algorithm. Proteins. *64*, 559-574.

Licchesi, J.D.F., Mieszczanek, J., Mevissen, T.E.T., Rutherford, T.J., Akutsu, M., Virdee, S., El Oualid, F., Chin, J.W., Ovaa, H., Bienz, M., and Komander, D. (2012). An ankyrin-repeat ubiquitin-binding domain determines TRABID's specificity for atypical ubiquitin chains. Nat. Struct. Mol. Biol. *19*, 62-71.

McCoy, A.J., Grosse-Kunstleve, R.W., Adams, P.D., Winn, M.D., Storoni, L.C., and Read, R.J. (2007). Phaser crystallographic software. J. Appl. Cryst. *40*, 658-674.

Mitchell, A., et al. (2015). The InterPro protein families database: the classification resource after 15 years. Nucl. Acids Res. *43*, D213-D221.

Pickart, C.M. and Raasi, S. (2005). Controlled synthesis of polyubiquitin chains. Method. Enzymol. *399*, 21-36.

Rawlings, N.D., Waller, M., Barrett, A.J., and Bateman, A. (2014). MEROPS: the database of proteolytic enzymes, their substrates and inhibitors. Nucl. Acids Res. *42*, D503-D509.

Rimsa, V., Eadsforth, T., and Hunter, W.N. (2011). The role of Co^{2+} in the crystallization of human SENP1 and comments on the limitations of automated refinement protocols. Acta Crytallogr. F Struct. Biol. Cryst. Commun. *67*, 442-445.

Söding, J., Biegert, A., and Lupas, A.N. (2005). The HHpred interactive server for protein homology detection and structure prediction. Nucl. Acids Res. *33*, W244-W248.

Stivala, A., Wybrow, M., Wirth, A., Whisstock, J., and Stuckey, P. (2011). Automatic generation of protein structure cartoons with Pro-origami. Bioinformatics. *27*, 3315-3316.

Tamura, K., Dudley, J., Nei, M., and Kumar, S. (2007). MEGA4: Molecular Evolutionary Genetics Analysis (MEGA) software version 4.0. Mol. Biol. Evol. *24*, 1596-1599.

Terwilliger, T.C., Adams, P.D., Read, R.J., McCoy, A.J., Moriarty, N.W., Grosse-Kunstleve, R.W., Afonine, P.V., Zwart, P.H., and Hung, L.W. (2009). Decision-making in structure solution using Bayesian estimates of map quality: the PHENIX AutoSol wizard. Acta Crytallogr. D Biol. Crystallogr. *65*, 582-601.

Terwilliger, T.C., Grosse-Kunstleve, R.W., Afonine, P.V., Moriarty, N.W., Zwart, P.H., Hung, L.W., Read, R.J., and Adams, P.D. (2008). Iterative model building, structure refinement and density modification with the PHENIX AutoBuild wizard. Acta Crytallogr. D Biol. Crystallogr. *64*, 61-69.
Yu, X.J., McGourty, K., Liu, M., Unsworth, K.E., and Holden, D.W. (2010). pH sensing by intracellular *Salmonella* induces effector translocation. Science. *328*, 1040-1043.
