# Supplementary material for: The Molecular Basis for Ubiquitin and Ubiquitin-like Specificities in Bacterial Effector Proteases
Source: Mol Cell. 2016 Jul 21;63(2):261–76. doi: 10.1016/j.molcel.2016.06.015 (PMC4961225; doi:10.1016/j.molcel.2016.06.015)
Supplement: Document S2. Article plus Supplemental Information [file mmc2.pdf]

Molecular Cell

Article

The Molecular Basis for Ubiquitin and Ubiquitin-like Specificities in Bacterial Effector Proteases

Graphical Abstract

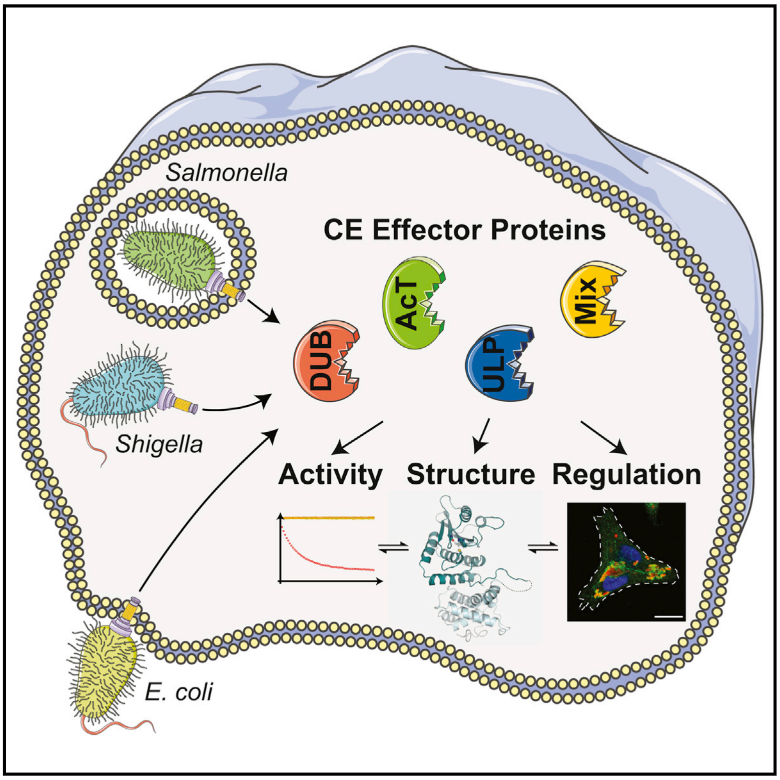

Authors

Jonathan N. Pruneda,
Charlotte H. Durkin, Paul P. Geurink,
Huib Ovaa, Balaji Santhanam,
David W. Holden, David Komander

Correspondence

dk@mrc-lmb.cam.ac.uk

In Brief

Focusing on examples from pathogenic bacteria, Pruneda et al. examine a family of proteases that displays remarkably distinct specificities toward ubiquitin and ubiquitin-like modifications. Leveraging structural and functional data, the authors derive mechanisms through which substrate specificity is achieved and redefine relationships within the enzyme family across kingdoms of life.

Highlights

- Bacterial CE proteases exhibit distinct ubiquitin/ubiquitin-like specificities
- Substrate specificity is acquired through variability in three common regions
- Structural and functional data redefine CE clan relationships across kingdoms
- CE effectors are fitted with accessory domains that modulate function

Accession Numbers

5HAF
5HAG
5HAM
5JP3
5JP1

Pruneda et al., 2016, Molecular Cell *63*, 261–276
July 21, 2016 © 2016 The Authors. Published by Elsevier Inc.
http://dx.doi.org/10.1016/j.molcel.2016.06.015

CellPress

The Molecular Basis for Ubiquitin and Ubiquitin-like Specificities in Bacterial Effector Proteases

Jonathan N. Pruneda,[1] Charlotte H. Durkin,[2] Paul P. Geurink,[3] Huib Ovaa,[3] Balaji Santhanam,[4] David W. Holden,[2] and David Komander[1,*]
[1]Division of Protein and Nucleic Acid Chemistry, MRC Laboratory of Molecular Biology, Francis Crick Avenue, Cambridge CB2 0QH, UK
[2]Section of Microbiology, MRC Centre for Molecular Bacteriology and Infection, Imperial College London, London SW7 2AZ, UK
[3]Division of Cell Biology, Netherlands Cancer Institute, Plesmanlaan 121, 1066 Amsterdam, the Netherlands
[4]Division of Structural Studies, MRC Laboratory of Molecular Biology, Francis Crick Avenue, Cambridge CB2 0QH, UK
*Correspondence: dk@mrc-lmb.cam.ac.uk
http://dx.doi.org/10.1016/j.molcel.2016.06.015

SUMMARY

Pathogenic bacteria rely on secreted effector proteins to manipulate host signaling pathways, often in creative ways. CE clan proteases, specific hydrolases for ubiquitin-like modifications (SUMO and NEDD8) in eukaryotes, reportedly serve as bacterial effector proteins with deSUMOylase, deubiquitinase, or, even, acetyltransferase activities. Here, we characterize bacterial CE protease activities, revealing K63-linkage-specific deubiquitinases in human pathogens, such as *Salmonella*, *Escherichia*, and *Shigella*, as well as ubiquitin/ubiquitin-like cross-reactive enzymes in *Chlamydia*, *Rickettsia*, and *Xanthomonas.* Five crystal structures, including ubiquitin/ubiquitin-like complexes, explain substrate specificities and redefine relationships across the CE clan. Importantly, this work identifies novel family members and provides key discoveries among previously reported effectors, such as the unexpected deubiquitinase activity in *Xanthomonas* XopD, contributed by an unstructured ubiquitin binding region. Furthermore, accessory domains regulate properties such as subcellular localization, as exemplified by a ubiquitin-binding domain in *Salmonella* Typhimurium SseL. Our work both highlights and explains the functional adaptations observed among diverse CE clan proteins.

INTRODUCTION

Bacterial colonization and proliferation within a eukaryotic host depends on processes that antagonize immune responses, ensure bacterial survival, and promote replication. For this, bacteria employ a repertoire of effector proteins, ranging in number from dozens to hundreds, which are directly delivered into the host cell by sophisticated secretion machineries (Figueira and Holden, 2012). Once inside, these effector proteins can hijack host factors and catalyze chemical modifications that are, in some cases, entirely foreign to the eukaryotic system (Salomon and Orth, 2013).

Bacterial effectors often target kinase cascades involved in inflammation (Salomon and Orth, 2013) or the eukaryotic ubiquitin (Ub) system (Corn and Vucic, 2014) to promote bacterial pathogenicity. Ub regulates countless cellular processes by forming structurally and functionally distinct polymeric chains that can be used independently or in concert during complex signaling cascades (Swatek and Komander, 2016). In addition, ubiquitin-like (Ubl) proteins, such as NEDD8, SUMO, and ISG15, play similarly important roles in host cell biology (van der Veen and Ploegh, 2012) and invasion response pathways (Liu et al., 2013; Radoshevich et al., 2015).

Ub and Ubl modifications are tightly regulated by discrete families of proteases. Humans encode ~80 active deubiquitinases (DUBs) that are unable to hydrolyze SUMO or NEDD8, although there are a few exceptions (Clague et al., 2013). The CE protease clan in humans consists of six SUMO-specific SENPs and the NEDD8-specific NEDP1/SENP8 that are collectively termed Ubl proteases (ULPs) (Ronau et al., 2016). Mechanisms dictating the exquisite specificities of these SUMO and NEDD8 proteases have been characterized in detail (Reverter and Lima, 2004; Reverter et al., 2005; Shen et al., 2005).

Bacteria and viruses also encode CE clan enzymes as effectors to interrupt eukaryotic host response processes (Ronau et al., 2016). Importantly, the characterized members from bacteria display diverse enzymatic activities and include not only ULPs (Kim et al., 2008; Orth et al., 2000) but also DUBs (Rytkönen et al., 2007; Misaghi et al., 2006; Zhou et al., 2005; Ye et al., 2007). In addition, some CE effectors, such as *Yersinia pestis* YopJ, display an unusual Ser/Thr acetyltansferase activity (Mittal et al., 2006; Mukherjee et al., 2006; Jones et al., 2008). Hence, it appears that the CE fold is highly versatile and adaptable and has evolved in bacterial pathogens to accommodate a range of enzymatic activities. However, the proteolytic activities and level of cross-reactivity in CE clan effectors are currently unclear since comprehensive comparisons have not been performed, and the molecular basis for achieving distinct target specificities from a single enzyme fold is unknown. Published

This is an open access article under the CC BY license (http://creativecommons.org/licenses/by/4.0/).

structures of individual bacterial (Chosed et al., 2007; Sheedlo et al., 2015) and viral examples (Ding et al., 1996) show a similarity to eukaryotic ULPs, but fall short of providing generalizable themes to explain diverse substrate specificities. The uncertainties regarding activity within this diverse family of enzymes have hindered a detailed understanding of the potential role(s) carried out during bacterial invasion.

To address this shortcoming, we cloned and expressed a panel of bacterial CE enzymes for in-depth biochemical and structural analysis. We identify several Ub-specific CE proteases, including the first DUBs in *Shigella* (ShiCE) and *Rickettsia* (RickCE), and show that most prefer K63-linked chains. Ub and K63 specificity is explained by a set of crystal structures, which show immense diversity in their convergent adaptations of a Ub recognition site. By comparing these bacterial examples with their Ubl-specific counterparts in eukaryotes, we identify and define three regions of variability within the CE fold that can be tailored to suit Ub or Ubl specificity. With this knowledge we revisit the *Xanthomonas* effector protein XopD, a reported deSUMOylase in plants, to identify an extended construct that targets not only SUMO but, surprisingly, also Ub modifications. Structures of Ub- and SUMO-bound XopD reveal striking plasticity in substrate recognition, due, in large part, to an N-terminal low-complexity Ub binding region (LC-UBR) that binds the Ub Ile44 patch but lacks secondary structure. The cumulative structural and functional data enable construction of a robust family dendrogram for CE clan enzymes across kingdoms. Finally, we uncover the functional importance for accessory domains outside the CE fold that, in the case of SseL, serve to target the effector to *Salmonella*-containing vacuoles (SCVs) during infection. Our comprehensive analysis of bacterial proteases redefines the roles they play during infection and establishes a framework for cross-kingdom relationships among the entire CE clan.

RESULTS

Selection and Properties of Bacterial CE Clan Effectors

While the presence of CE clan effectors in bacteria and biochemical functions for some members have been described, a comprehensive comparison of proteolytic specificities has not been performed. To fill this gap, we selected a divergent set of enzymes from human bacterial pathogens (Figure 1A), including putative DUBs SseL (*Salmonella enterica* serovar Typhimurium, hereafter *S*. Typhimurium [Rytkönen et al., 2007]), ChlaDUB1 (*Chlamydia trachomatis* [Misaghi et al., 2006]), and ElaD (*Escherichia coli* [Catic et al., 2007]), as well as Ser/Thr acetyltransferases YopJ (*Yersinia pestis* [Mukherjee et al., 2006; Mittal et al., 2006]) and AvrA (*S*. Typhimurium [Jones et al., 2008]). To this, we added entirely uncharacterized CE clan proteins RickCE (*Rickettsia bellii*, GenBank: ABE04279.1), LegCE (*Legionella pneumophila ssp. pneumophila*, GenBank: AAU28953.1), and ShiCE (*Shigella flexneri*; GenBank: EGK20985.1) (Figure 1A). In all of the selected enzymes, the catalytic CE fold is present in the context of additional domains of unknown function (Figures 1A and S1A), only few of which bear sequences similar to domains found in eukaryotes (see below). Following cloning and the subsequent *E. coli* expression of suitable constructs (Figure 1A), the panel of CE enzymes was purified to homogeneity (Figure 1B). The CE fold contains a conserved catalytic Cys (Figure S1B), which was mutated in each enzyme to Ala (e.g., SseL^{CA}) and purified analogously (Figure 1B).

Activities of CE Clan Bacterial Effectors

Cys-based DUBs can be covalently modified by Ub-based suicide probes, in which the C terminus has been modified with an electrophilic trap, such as propargylamine (Ub-PA; Ekkebus et al., 2013). SseL, ChlaDUB1, and ElaD, as well as the previously uncharacterized ShiCE and RickCE, were all covalently modified by Ub-PA in a catalytic Cys-dependent manner, while the remaining enzymes, including YopJ, AvrA, and LegCE, remained unmodified (Figure 1C).

To assess all Ub and Ubl proteolysis in a simple, parallel assay, we used fluorescence polarization (FP) to measure hydrolysis of Ub, SUMO1, NEDD8, and ISG15 substrates that were isopeptide linked to a fluorescently labeled Lys-Gly peptide (Figure 1D) (Geurink et al., 2012; Basters et al., 2014). Isopeptide linkage imposes a more chemically precise environment for a Ub/Ubl modification. When tested against the full panel, human SENP1 and NEDP1 exclusively cleaved SUMO1- and NEDD8-modified peptides, respectively (Figure S1C). YopJ, AvrA, and LegCE showed no proteolytic activity in our assays, regardless of the presence of the cofactor inositol hexakisphosphate (IP6) (Mittal et al., 2010) (Figures 1E and S1D–S1F). Furthermore, AvrA showed no sign of an interaction with Ub when measured by nuclear magnetic resonance (NMR) titration (Figure S1G). YopJ and AvrA were, however, highly active in an in vitro autoacetylation assay with IP6, and LegCE, likewise, showed modest acetylation activity compared to its catalytically inactive mutant (Figure S1H), indicating that these effectors are dedicated acetyltransferases.

Interestingly, the remaining bacterial effectors SseL, ElaD, and ShiCE are Ub-specific proteases, and ChlaDUB1 and RickCE cleave both Ub- and, to a lesser extent, NEDD8-modified peptides (Figure 1E). Like NEDP1, ChlaDUB1 can remove the C-terminal five amino acids from pro-NEDD8 with peptidase activity (Figure S1I), but does not cleave the regulatory NEDD8 modification from the cullin RING ligase adaptor Cul1 (Figure S1J).

As there are many examples of interesting polyUb chain specificity in eukaryotic DUBs (Mevissen et al., 2013), it was important to test if any bacterial CE DUBs showed a preference for particular polyUb substrates. Early studies on SseL indicated higher activity for K63-linked over K48-linked chains (Rytkönen et al., 2007), and *Legionella pneumophila* effector SdeA prefers K63 linkages over K11 and K48 linkages (Sheedlo et al., 2015), but preference among all eight possible Ub:Ub linkages for these and all other enzymes have remained unstudied. Strikingly, we found that CE DUBs encoded by human pathogens showed strong preference for K63-linked chains, only targeting K48 and K11 chains at later time points or higher enzyme concentrations (Figures 1F and S1K–S1M), indicating significant pressure to remove this important infection-associated post-translational modification during invasion (Corn and Vucic, 2014).

Structural Analysis of the CE Proteases

The new functionalities in CE clan effectors prompted structural characterization to reveal how this enzyme fold evolved to

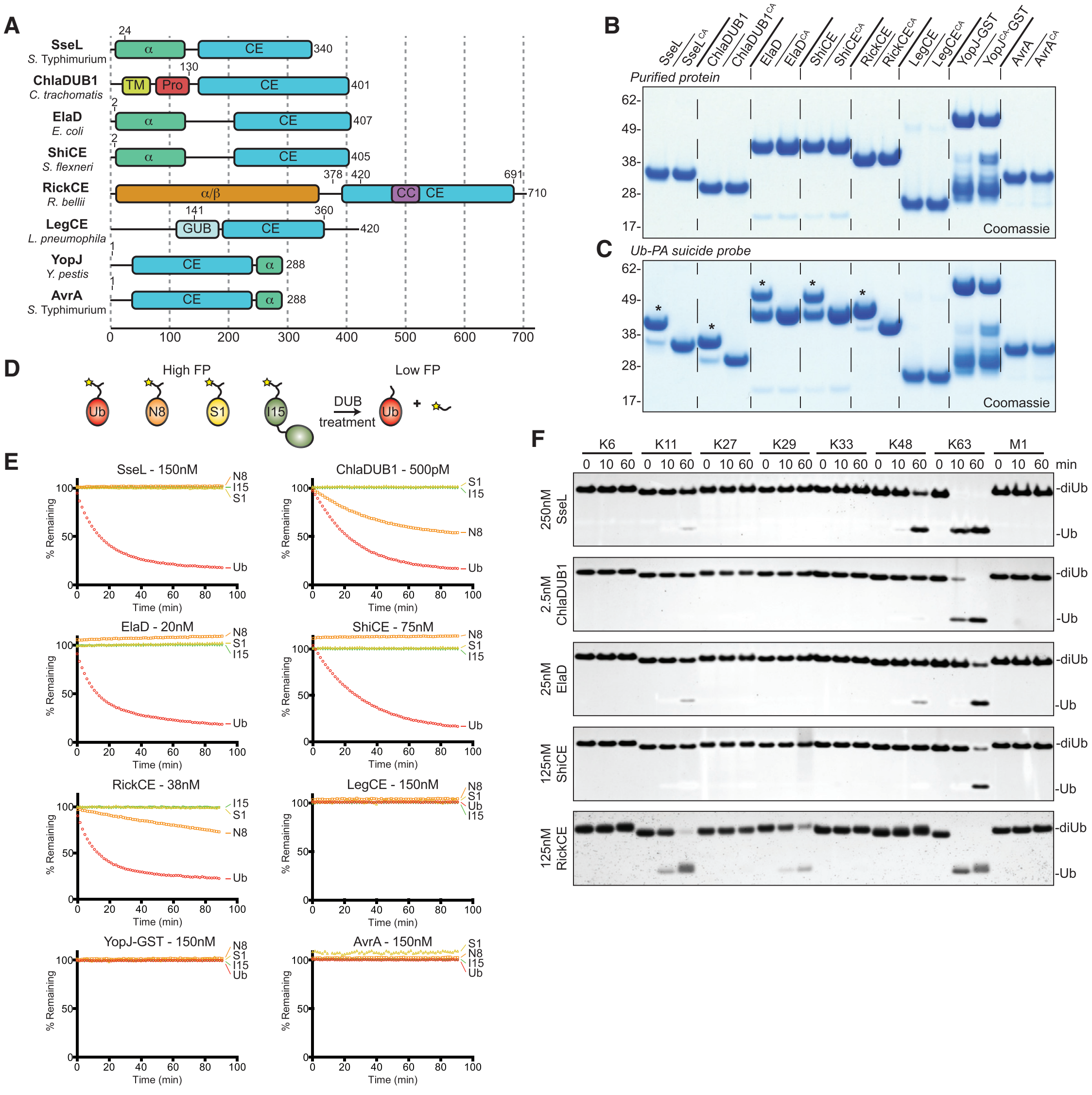

Figure 1. CE Effector Proteins Demonstrate Mixed Activities

(A) Domain annotation of selected bacterial CE effectors. Construct boundaries and accessory domains used in this study are indicated. α, predicted α-helical domain; α/β, predicted α/β fold; TM, transmembrane helix; Pro, proline-rich sequence; GUB, wall-associated receptor kinase galacturonan-binding (GUB_WAK_bind) domain; CC, coiled coil.

(B) Purified CE enzymes from bacteria. CA, catalytic Cys-to-Ala mutant. GST-YopJ contains co-purifying degradation products at lower molecular weight.

(C) Suicide probe reaction following incubation with the Ub-PA probe for 1 hr at room temperature. Asterisks (*) mark catalytic cysteine-dependent reactivity.

(D) Schematic of the Ub/Ubl substrate cleavage assay. Ub/Ubl modifiers are isopeptide linked to a TAMRA-labeled Lys-Gly peptide, and fluorescence polarization (FP) is used to monitor cleavage over time. N8, NEDD8; S1, SUMO1; I15, ISG15.

(E) Normalized FP measured as a function of time following the addition of the listed active CE enzymes to the TAMRA-linked Ub/Ubl reagents.

(F) Linkage specificity analysis for bacterial CE DUBs active in the TAMRA assay. A silver-stained SDS-PAGE gel shows diUb hydrolysis over time.

See also Figure S1.

accommodate such divergent proteolytic activities. We determined high-resolution crystal structures of the CE clan effectors SseL, RickCE, and ChlaDUB1 (Figures 2A and S2A–S2C; Table 1). Due to low-sequence similarities, all structures were experimentally phased (see the Experimental Procedures). The crystallized full-length SseL construct comprised an additional N-terminal ~135-residue helical domain (see below). The catalytic CE fold bears similarity to eukaryotic CE proteases (Figure 2B), and the conserved active-site residues are required for DUB activity (Figures S2D and S2E). However, several regions of high variability were immediately apparent.

A comparison to the previously determined SENP2-SUMO2 and NEDP1~NEDD8 complexes (Figure 2C) (Reverter and Lima, 2004; Reverter et al., 2005; Shen et al., 2005) revealed differences among bacterial CE DUBs in three variable regions (VRs; Figure 2D) near the substrate binding (S1) site that were

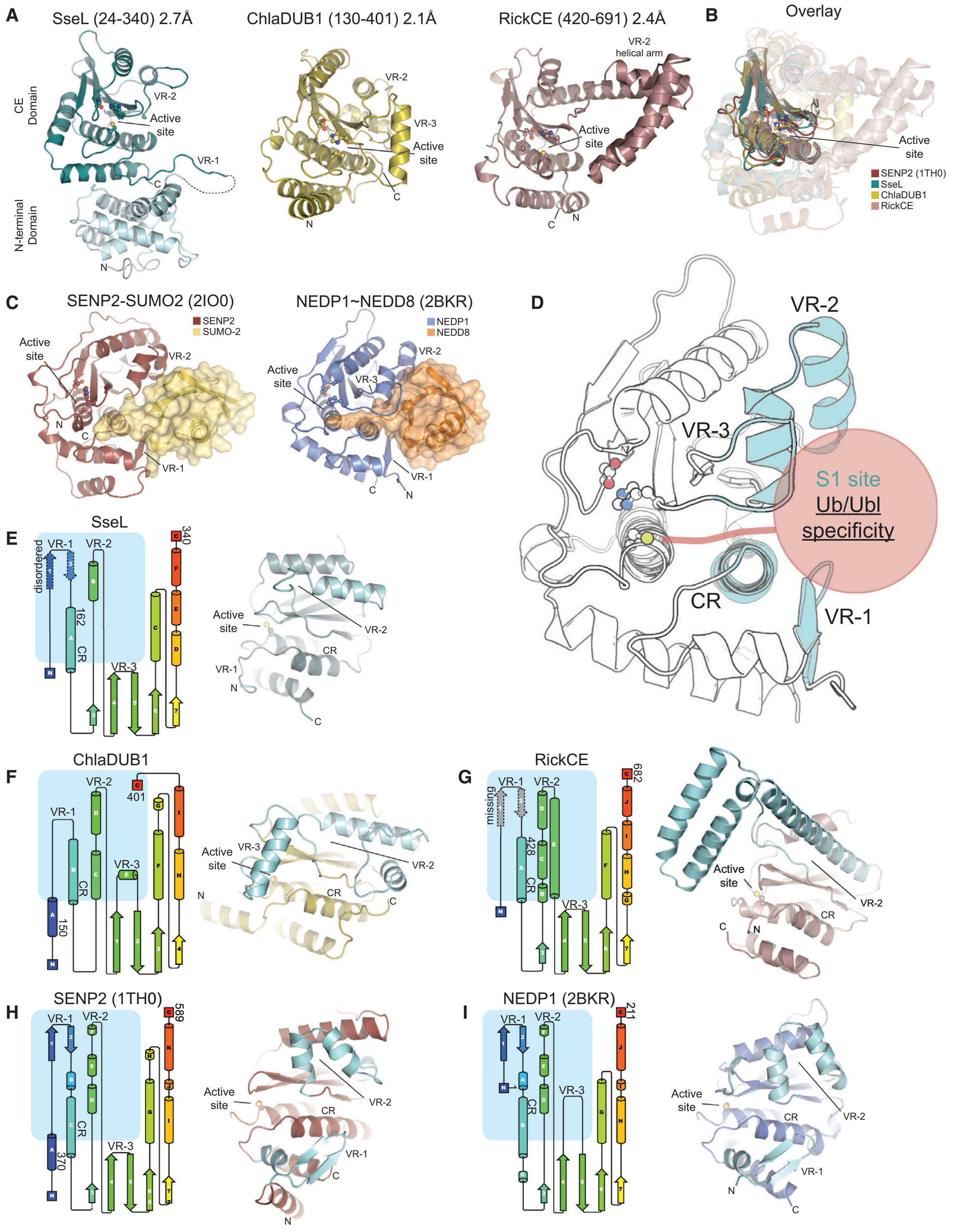

(legend on next page)

arranged around a structurally conserved helix (constant region, CR). Variations within these three regions are found in all examples of the CE fold (Figures 2E–2I), as detailed below.

The first variable region, VR-1, is found at the beginning of the CE fold (Figure 2D), which in NEDP1 and the SENP family is a β-hairpin that forms electrostatic interactions with the Ubl (Figures 2C, 2H, and 2I). In bacterial examples, the analogous region is either disordered in the absence of substrate (SseL; Figures 2A and 2E) or not part of the crystallized construct (RickCE; Figures 2A and 2G). A corresponding VR-1 region is absent in ChlaDUB1; interestingly, the ChlaDUB1 C terminus occupies this space instead (Figures 2A and 2F).

The second variable region, VR-2, is located between the first two strands of the central β sheet, and in the eukaryotic examples forms hydrophobic contacts with the Ubl β sheet (Figure 2D). Intriguingly, this region is similar among the Ubl proteases (Figures 2H and 2I), but diverse among the CE-fold DUBs (Figures 2E–2G). In fact, RickCE has an ~75-residue insertion at this site that forms a helical arm in the crystal structure (Figure 2A).

The third variable region, VR-3, is an insertion between the second and third strands of the β sheet, just preceding the catalytic His (Figure 2D). NEDP1 and ChlaDUB1 contain an insertion of 7 and 24 residues, respectively, in what is a short β-turn in the other structures (Figures 2A, 2C, 2F, and 2I). In NEDP1, VR-3 forms an extended loop that guides the NEDD8 C terminus into the active site through backbone hydrogen bonding (Figures 2C and 2I). In contrast, ChlaDUB1 forms a helix that may serve a similar purpose in recognition of the Ub C terminus (Figures 2A and 2F).

Overall, the dramatic differences in substrate recognition imparted by CE-fold variable regions suggest that bacteria have adopted different strategies of altering the CE-fold to evolve DUB activity.

Re-evaluation of XopD Ub/Ubl Specificity

During our analysis of the CE fold, we noticed that a putative VR-1 region had been omitted in the crystallized construct of the *Xanthomonas campestris* effector XopD (Figure 3A) (Chosed et al., 2007). Given the importance of all three variable regions in the eukaryotic Ubl-bound structures (Figures 2C, 2H, and 2I), we compared the activity of XopD without (ΔVR-1; amino acids [aa] 335–515) and with the analogous VR-1 region (+VR-1, aa 298–515). In a suicide probe assay, XopD ΔVR-1 reacted solely with *Solanum lycopersicum* tomato SUMO (tSUMO), but not with human SUMO1, as reported (Chosed et al., 2007) (Figure 3B). To our surprise however, XopD +VR-1 was not only more reactive with tSUMO but also strikingly now reacted with Ub (Figures 3B and S3A). The same Ub/tSUMO dual specificity was observed in an FP-based assay of KG-modified substrates, where both Ub and tSUMO were cleaved with similar high efficiencies in a VR-1-dependent manner (Figure 3C). To our knowledge, XopD is the first cross-reactive Ub and (t)SUMO isopeptidase. Moreover, unlike other CE effector DUBs, XopD prefers K11, K29, and K48 linkages and only cleaves K63- and K6-linked chains to a lesser extent (Figure 3D). This distinct specificity profile for Ub chain substrates highlights that the versatile CE fold can not only be adapted to distinct Ub/Ubl specificities (which depends on S1 site interactions, see above) but also can be adapted to modulate Ub chain preferences, due to changes in the S1' site (see below). Biologically, this reiterates the pressure placed on human pathogens to deal with K63 linkages, while the plant pathogen clearly prioritizes distinct linkages and SUMO, which may, hence, mediate anti-bacterial signaling in plants.

Molecular Analysis of XopD Cross-Specificity

To understand what structural adaptations enable XopD cross-reactivity, we determined the crystal structures of covalent XopD~Ub (2.9 Å; Figures 3E and S3B; Table 1) and XopD~tSUMO (2.1 Å; Figures 3F and S3C; Table 1) complexes. The XopD~Ub structure contained four copies in the asymmetric unit, all of which were similar (0.62 Å root-mean-square deviation [RMSD] over XopD and Ub; Figure S3D). Moreover, the core fold of XopD itself was highly similar in the Ub-bound, tSUMO-bound, and the published apo structure (PDB: 2OIV, 0.61 Å RMSD over aa 338–515; Figure S3E). To our surprise, the VR-1 responsible for much of the observed XopD protease activity was not a β-hairpin as had been observed in eukaryotic Ubl proteases (Figure 2D), but, instead, was extended and devoid of secondary structure. Furthermore, the extended VR-1 conformation was completely different between the Ub- and tSUMO-bound complexes. A comparison of the two structures clearly explains the observed VR-1 dependence in activity. While the Ub S1 site primarily consists of contacts to VR-1 and some to VR-2, tSUMO binding displays the opposite trend, with the majority of interactions taking place at VR-2 (Figures 3E–3G).

In the Ub-bound structure, XopD VR-1 threads underneath the Ub Ile44 hydrophobic patch, with residues Pro322 and Val325 making contacts to Ub Ile44 and His68, and also coordinates Ub Arg72 with Asp327 (Figure 3H, foreground). Additionally, Met374 of VR-2 contacts the Leu8 loop of Ub (Figure 3H, background). Incorporation of either I44A or R72A mutations into Ub suicide probes resulted in a dramatic loss in reactivity to XopD (Figure 3J).

Figure 2. Structural Analysis of Bacterial CE Deubiquitinases

(A) Cartoon representations of SseL (2.7 Å, teal), ChlaDUB1 (2.1 Å, yellow), and RickCE (2.4 Å, violet) aligned on the catalytic triad (ball and stick).

(B) Superposition of the CE core based on the catalytic triad for structures in (A) and human SENP2 (PDB: 1TH0).

(C) Representations of the human SENP2-SUMO2 noncovalent complex (PDB: 2IO0, left) and NEDP1~NEDD8 covalent complex (PDB: 2BKR, right) illustrating a common Ubl binding site.

(D) Schematic of the CE fold based on the NEDP1 structure (PDB: 2BKR), highlighting the constant region (CR) and variable regions (VR) that form the Ub/Ubl-binding S1 site (blue).

(E–I) Left: topology diagrams for CE structures, with the S1 binding site highlighted (blue box). Structure boundaries are indicated by numbers. Features in dashed outline are either disordered or outside the crystallized constructs, as marked. Right: view of the S1 substrate binding site in CE-fold structures, with variable regions highlighted in cyan and labeled accordingly.

See also Table 1 and Figure S2.

Table 1. Data Collection and Refinement Statistics

	SseL 24-340 SeMet	SseL 24-340	ChlaDUB1 130-401 SeMet	ChlaDUB1 130-401	RickCE 420-691 SeMet	RickCE 420-691	XopD∼Ub 298-515	XopD∼tSUMO 298-515
**Data Collection**								
Beamline	ESRF ID23-1	ESRF ID23-1	DLS I03	DLS I03	DLS I04	DLS I04-1	DLS I04-1	DLS I04-1
Space group	$P\ 3_1\ 1\ 2$	$P\ 3_1\ 1\ 2$	$I\ 2\ 3$	$I\ 2\ 3$	$H\ 3$	$H\ 3$	$C\ 2$	$P\ 6_4$
Cell dimensions *a*, *b*, *c* (Å)	113.30, 113.30, 166.62	113.01, 113.01, 166.60	133.45, 133.45, 133.45	133.29, 133.29, 133.29	178.33, 178.33, 56.81	173.50, 173.50, 55.52	117.75, 132.28, 117.31	119.10, 119.10, 50.46
α, β, γ (°)	90, 90, 120	90, 90, 120	90, 90, 90	90, 90, 90	90, 90, 120	90, 90, 120	90, 105.84, 90	90, 90, 120
Wavelength (Å)	0.9791	0.9791	0.9798	0.9763	0.9795	0.9174	0.9282	0.9282
Observed reflections	289,672	159,309	216,565	118,321	223,044	89,892	148,793	82,488
Unique reflections	15,721	33,454	12,344	23,093	8,501	24,203	37,920	23,605
Resolution (Å)	98.12–3.50 (3.83–3.50)	84.39–2.70 (2.83–2.70)	94.36–2.60 (2.72–2.60)	35.62–2.10 (2.16–2.10)	53.31–3.50 (3.83–3.50)	52.08–2.40 (2.49–2.40)	43.30–2.90 (3.03–2.90)	59.55–2.10 (2.16–2.10)
R_{merge}	0.129 (0.319)	0.122 (0.627)	0.232 (1.530)	0.078 (0.521)	0.418 (2.726)	0.085 (0.596)	0.128 (0.568)	0.089 (0.465)
$I/\sigma I$	18.3 (10.9)	7.9 (2.4)	11.0 (2.2)	11.1 (2.8)	11.3 (3.6)	9.5 (2.0)	8.1 (2.4)	8.7 (2.0)
Completeness (%)	100.0 (100.0)	99.3 (99.8)	100.0 (100.0)	99.9 (99.8)	100.0 (100.0)	99.2 (99.7)	99.0 (99.1)	98.1 (99.4)
Redundancy	18.4 (17.4)	4.8 (4.9)	17.5 (18.0)	5.1 (5.1)	26.2 (26.2)	3.7 (3.8)	3.9 (4.0)	3.5 (3.4)
Phasing								
Method	SAD		SAD		SAD		MR (PDB: 2OIV and 1UBQ)	MR (PDB: 2OIV and 2IO0)
Resolution	3.5		2.6		4.0			
Anom completeness	100.0 (100.0)		100.0 (100.0)		100.0 (100.0)			
Anom multiplicity	9.5 (8.8)		8.9 (9.1)		12.8 (12.8)			
<FOM>	0.319		0.37		0.318			
Refinement								
Reflections in test set		1,693		1,112		1,190	1,835	1,281
R_{work}/R_{free}		18.7/22.8		18.3/20.8		16.4/21.7	24.0/28.6	18.1/21.5
Number of atoms								
Protein		4,868		2,135		4,025	8,176	2,173
Ligand/ion		3		6		0	16	26
Water		96		129		163	44	199
B factors								
Wilson *B*		45.6		34.5		36.8	43.2	24.8
Protein		53.3		38.4		42.1	46.3	29.7
Ligand/ion		59.1		50.0		–	44.1	50.7
Water		43.6		45.0		41.6	30.9	38.0
Rmsd								
Bond lengths (Å)		0.003		0.004		0.008	0.005	0.004
Bond angles (°)		0.67		0.82		1.10	0.82	0.58
Ramachandran statistics (favored/allowed/outliers)		96.2/3.8/0		97.4/2.6/0		97.7/2.1/0.2	97.5/2.4/0.1	97.4/2.6/0

Values in parentheses are for the highest resolution shell. Anom, anomalous; rmsd, root-mean-square deviations; FOM, figure of merit. See also Figures S2 and S3.

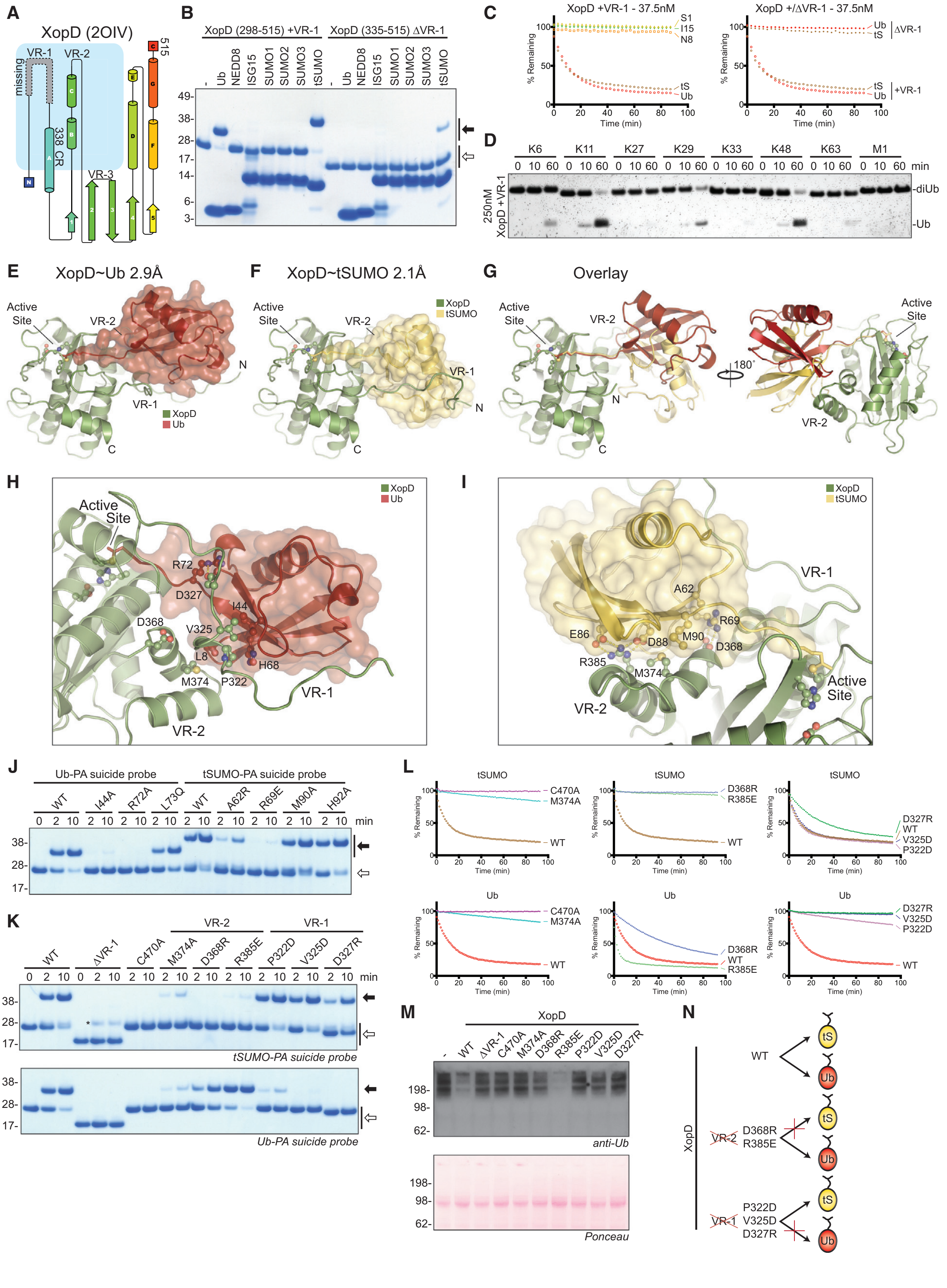

(legend on next page)

The VR-1 interface to the Ub Ile44 patch was highly unusual and unprecedented among published ubiquitin-binding domains, because such a low-complexity Ub binding region (LC-UBR) had not been previously described. Verifying the interaction in solution, NMR titration experiments following ^{15}N-labeled Ub revealed a number of select resonances that displayed small chemical shift perturbations and/or line broadening with increasing concentrations of the XopD +VR-1 construct (Figure S3G), all of which depended on the VR-1 region (Figure S3H). When mapped to the XopD∼Ub crystal structure, the changes observed in the NMR titration experiment nicely agree with contacts made between XopD VR-1 and the Ub Ile44 patch (Figure S3I).

In contrast, the bulk of contacts made in the tSUMO-bound structure are through VR-2, which forms multiple ionic interactions (XopD Arg385 and Asp368 contacting tSUMO Glu86/Asp88 and Arg69, respectively) surrounding a hydrophobic interface (XopD Met374 contacting tSUMO Met90) (Figure 3I). The tSUMO A62R or R69E mutation both resulted in a decrease in tSUMO suicide probe reactivity (Figure 3J). Surprisingly, a M90A mutation had much smaller effects (Figure 3J), suggesting that the small hydrophobic interface may play a lesser role in tSUMO recognition than does the extensive charge complementarity. Although highly divergent in sequence, the Ub and tSUMO C termini threaded into the active site similarly, with no apparent favoritism following Ub Arg72 (Figure S3F). Accordingly, neither a swap of Ub Leu73 to the analogous tSUMO Gln nor mutation of tSUMO His92 (equivalent to Ub Arg72) had a significant impact on suicide probe reactivity (Figure 3J).

Manipulating XopD Ub/Ubl Preferences

The Ub/tSUMO-bound structures were further confirmed by mutagenesis targeting interactions in VR-1 and VR-2 that were either common to both substrates or unique to one. In addition to the catalytic C470A mutation, a XopD M374A mutation in VR-2 was deleterious to both Ub and tSUMO recognition, as observed in a suicide probe assay (Figure 3K) and FP-based protease assay (Figure 3L). Additional mutations in VR-2 targeted the primary tSUMO interaction surface and resulted in either a slight loss (D368R) or a gain (R385E) in activity toward Ub substrates, while activity toward tSUMO was significantly reduced (Figures 3K–3L). In contrast, mutations targeting the primary Ub interaction in VR-1 (P322D, V325D, and D327R) all showed decreased activity toward Ub substrates with little-to-no effect on tSUMO recognition (Figure 3K–3L). Furthermore, recombinant XopD hydrolyzes endogenous Ub-modified proteins from *S. lycopersicum* protein extract in a manner consistent with the mutational analysis on biochemical substrates (Figure 3M).

Thus, the cross-reactive XopD uses adaptations of common variable regions (see Figure 2D) to specifically recognize both Ub and tSUMO. In particular VR-1 forms contacts unique to each substrate (Figures 3E, 3F, and 3N). The proline-rich, low-complexity VR-1 sequence bears a textbook resemblance to an intrinsically disordered region (mean IUPred disorder propensity score of 0.72 [Dosztányi et al., 2005]) and thus enables flexible binding properties ideally suited to provide dual specificity.

Understanding Ub/Ubl Specificity in CE Clan DUBs

The importance of variable regions in Ub recognition extends to each crystallized DUB (Figures 2E–2G) and also to the *Legionella* effector SdeA, which uses yet another variation of VR1-3 to recognize Ub (Sheedlo et al., 2015) (Figures S4A and S4B). Mutations in the S1 site of SseL confirmed the Ub binding mode predicted based on analogous Ub/Ubl-bound structures (Figure 4A). A conserved hydrophobic (Trp or Phe) at the start of the core CE fold is present in eukaryotic CE ULPs and corresponds to Met159 in SseL. SseL M159A mutation significantly reduced K63 diUb hydrolysis (Figures 4A and 4B). Likewise, solvent-exposed hydrophobic residues within SseL VR-2 (Tyr183 and Ile196) abolished or reduced DUB activity, respectively

Figure 3. Molecular Analysis of XopD Ub/Ubl Specificity

(A) Topology diagram, as in Figure 2E, for the XopD crystal structure (PDB: 2OIV). The crystallized construct lacks a potential VR-1 region.

(B) XopD constructs with VR-1 (+VR-1: 298–515) or without VR-1 (ΔVR-1: 335–515) were tested against a panel of Ub/Ubl suicide probes for 1 hr at room temperature (propargylamine-derived probes). Open arrow, unmodified; closed arrow, modified.

(C) FP assays as in Figure 1D, including a substrate derived from *S. lycopersicum* SUMO (tS, tomato SUMO). Left: Ub/Ubl specificity of XopD +VR-1. Right: comparison of XopD +VR-1 cleaving the Ub and tSUMO substrates (shown in left) with the ΔVR-1 construct.

(D) Linkage specificity analysis as in Figure 1F for XopD +VR-1.

(E) Cartoon representation of the 2.9 Å XopD∼Ub covalent complex crystal structure.

(F) Cartoon representation of the 2.1 Å XopD∼tSUMO covalent complex crystal structure.

(G) Overlay of structures shown in (E) and (F), illustrating differences in Ub/tSUMO conformation and usage of XopD variable regions. For clarity, VR-1 is not shown.

(H) Close-up of the XopD-Ub interaction. In the foreground, VR-1 coordinates Ub Arg72 and the Ile44 hydrophobic patch. In the background, VR-2 contacts the Ub Leu8 loop.

(I) Close-up of the XopD-tSUMO interaction. VR-2 forms the basis of tSUMO binding and makes a number of polar and salt-bridge interactions surrounding the hydrophobic contact of tSUMO Met90.

(J) Series of Ub and tSUMO suicide probe reactions performed on ice, testing interactions observed in the Ub/tSUMO domain, as well as in their C termini (propargylamine-derived probes). Open arrow, unmodified; closed arrow, modified.

(K) As in (J), for XopD mutations encompassing VR-1 and VR-2. Open arrow, unmodified; closed arrow, modified. Asterisk, contamination in tSUMO suicide probe.

(L) FP-based cleavage assays testing the XopD variants used in (K) against the Ub and tSUMO KG-modified substrates. Wild-type XopD data from (C) are shown for reference.

(M) *S. lycopersicum* protein extract blotted for total Ub (Ubi-1; Novus Biologicals) following a 1 hr room temperature treatment with the XopD variants used in (K) at 1 μM final concentration.

(N) Schematic summarizing the activities displayed by wild-type and VR-1/VR-2 mutant XopD variants.

See also Table 1 and Figure S3.

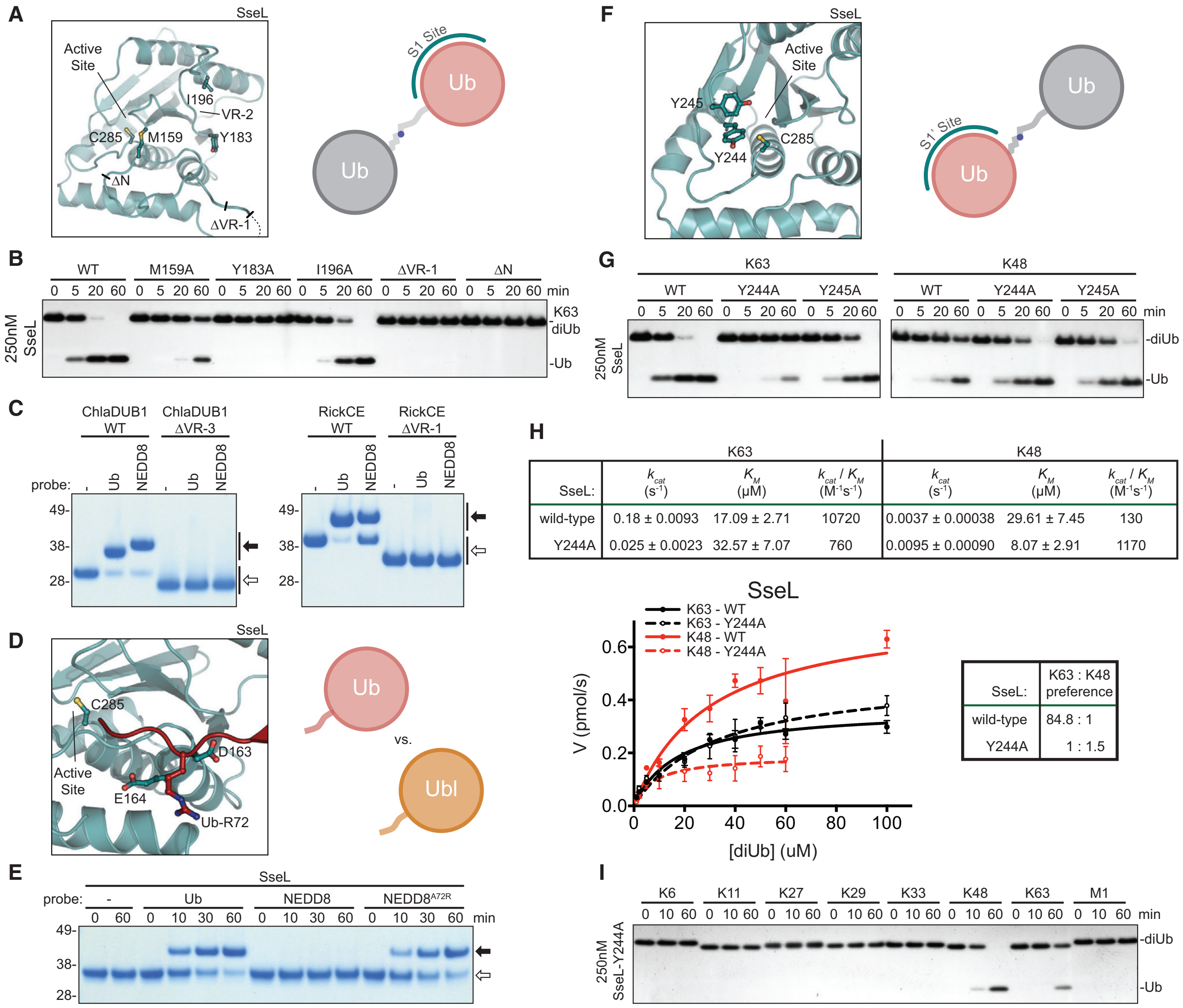

Figure 4. Structure-Based Manipulation of CE DUB Activities and Specificities

(A) Mutations and truncations designed in the SseL S1 binding site (SseL ΔVR-1: Δ138–147; SseL ΔN: aa 158–340).

(B) K63 diUb cleavage assay using mutants from (A).

(C) Suicide probe assay monitoring Ub and NEDD8 reactivity following 1 hr incubation at room temperature, for wild-type ChlaDUB1 and RickCE, and constructs containing truncated variable regions (ChlaDUB1 ΔVR-3: Δ250-272; RickCE ΔVR-1: aa 420-691; propargylamine-derived probes). Open arrow, unmodified; closed arrow, modified.

(D) Model of the Ub C terminus entering the SseL active site, based on analogous Ubl-bound structures. Putative acidic residues that can coordinate Ub Arg72 are shown.

(E) SseL suicide probe assay with Ub, NEDD8, and NEDD8 A72R probes performed at room temperature (chloroethylamine-derived probes). Open arrow, unmodified; closed arrow, modified.

(F) Close-up of the conserved hydrophobic S1' site of SseL.

(G) K63 and K48 diUb cleavage assays using S1' site mutations.

(H) Kinetic analysis of K48 and K63 diUb linkage preference for SseL wild-type and Y244A.

(I) Linkage specificity analysis for all diUb substrates, as in Figure 1F, for the SseL Y244A S1' site mutant.

See also Figure S4.

(Figures 4A and 4B). Interestingly, deletion of the putative substrate-binding VR-1 region alone (SseL ΔVR-1) or removal of the N-terminal domain including VR-1 (SseL ΔN) abolished diUb cleavage, suggesting that the disordered sequence indeed contributes to substrate binding (Figures 2E, 4A, and 4B).

The importance of unique variable regions in ChlaDUB1 and RickCE was also tested using Ub/Ubl suicide probes as a

measure of substrate recognition. Matching their specificity profiles (Figure 1E), ChlaDUB1 and RickCE can both form covalent adducts with Ub and NEDD8 suicide probes. Deletion of the inserted VR-3 helix of ChlaDUB1 (Figures 2A and 2F) or the predicted VR-1 outside the crystallized boundaries of RickCE (Figures 2A and 2G) resulted in a total loss in recognition of the Ub/Ubl probes (Figure 4C). In sum, the unique contributions from all three variable regions in XopD, SseL, ChlaDUB1, and RickCE are required for recognition of a Ub/Ubl substrate.

Changing Specificities for Ub/Ubl and polyUb Chain Linkage

Ub and NEDD8 are highly similar (58% identical, 85% similar), yet most CE-clan proteases, including SseL and XopD, distinguish between them with high specificity (Figures 1E, S1C, and S3C). A key difference between Ub and NEDD8 is an Arg-to-Ala substitution at position 72 in the Ub/Ubl C terminus. Arg72 of Ub is well-coordinated in the XopD~Ub structure (Figure 3H), as well as in other Ub-bound DUB structures (Ye et al., 2011). Analysis of a modeled SseL~Ub complex highlighted two acidic residues, Asp163 and Glu164, that may also coordinate Ub Arg72 (Figure 4D). Importantly, a NEDD8 suicide probe with an A72R mutation (Ye et al., 2011) covalently modified SseL as efficiently as the wild-type Ub probe (Figure 4E). Likewise, incorporation of the A72R mutation imparts reactivity of the NEDD8 suicide probe toward XopD (Figure S4C). Thus, in addition to large-scale changes in the variable regions of the S1 site, proper coordination of the Ub/Ubl C terminus contributes an added level of substrate specificity.

Unlike XopD (Figure 3D), CE effectors from human pathogens strongly prefer K63-linked chains (Figure 1F). This suggests there must be a linkage specificity-imposing S1' Ub binding site that participates in Ub recognition (Mevissen et al., 2013), though such an S1' site in CE DUBs has not been annotated. Examination of the SseL, ChlaDUB1, and RickCE structures revealed a conserved hydrophobic region near the catalytic Cys (Figures 4F and S4D), and mutating residues in this patch on SseL simultaneously reduced activity toward K63-linked diUb, while improving hydrolysis of K48-linked diUb (Figure 4G). This was quantified by a fluorescence polarization-based diUb cleavage assay (Keusekotten et al., 2013). Based on the Michaelis-Menten kinetic parameters, wild-type SseL displays an approximately 85-fold preference for K63 chains over K48; this is predominantly the result of a difference in k_{cat} (Figure 4H). The Y244A mutation causes an approximately 15-fold reduction in k_{cat}/K_m toward K63-linked chains, while simultaneously increasing k_{cat}/K_m toward K48-linked chains 10-fold. Hence, SseL Y244A displays a ~1.5-fold preference for K48 over K63 diUb (Figure 4H); this preference is also confirmed when tested against a panel of all eight Ub chain types (Figure 4I).

Together, mutagenesis work establishes the location of the S1' Ub binding site in CE clan DUBs, and the ability to modulate Ub/Ubl, as well as linkage specificity with point mutations, highlights the versatility and adaptability of the CE protease fold.

Bioinformatic Analysis of the CE Clan Highlights Distinct Functional Families

Using our new CE protease structures as a guide, we expanded and refined the sequence alignment of CE clan members in eukaryotes, viruses, and bacteria (Figure S5). In addition to the active site, we identified several conserved positions important for domain structure and substrate recognition, including the semi-conserved Trp at the start of the CE fold and a highly conserved Trp following the catalytic His.

Rewardingly, reconstruction of the dendrogram for the entire CE clan shows an interesting segregation of families that agrees with the demonstrated functional properties for these enzymes (Figure 5). In agreement with their dual Ub/SUMO protease activity, the XopD-like family, including XopD and examples from other plant-associated bacteria, are most closely related to the eukaryotic ULP families. As dedicated DUBs, the SseL-like examples (including ElaD and ShiCE) also segregate near the XopD/ULP subfamily. The Ub/NEDD8 cross-reactive enzymes ChlaDUB1 and RickCE fall into their own clade. Interestingly, the YopJ family, that also includes *Legionella* LegCE, lacks, e.g., conserved Trp residues (Figure S5) and is most divergent, consistent with their roles as dedicated acetyltransferases (Figures 1E and S1H).

CE fold proteins from viruses constitute three additional groups. The first group (viral group I) includes examples from Adenoviridae and clusters near the Ub/Ubl proteases. This group includes the adenovirus L3 23K proteinase, which, in addition to general protease activity, also possesses DUB activity (Balakirev et al., 2002).

Viral groups II and III are more divergent and also include bacterial proteins (Figure 5). The *Legionella* effector SidE (Figures S4A and S4B) was independently identified by our sequence analysis as a part of viral group II and has since been shown to display mixed activities toward Ub, NEDD8, and, interestingly, ISG15 (Sheedlo et al., 2015). Viral group III, which contains Vaccinia virus protein I7L, has been shown to target an Ala-Gly-Xaa motif instead of the Gly-Gly-Xaa motif of Ub/Ubl modifiers, and hence, members within the viral group III might be more general proteases dedicated to cleavage events necessary for viral maturation (Byrd et al., 2003). Therefore, our structural and functional analysis of divergent CE clan members has enabled the refinement of relationships across kingdoms, has enabled identification of further examples in other species, and allows a distinction between dedicated DUBs, dedicated ULPs, and specialized acetyltransferases.

CE Clan Proteases Are Further Functionalized with Accessory Domains

In addition to the catalytic fold, bacterial CE clan effectors contain additional N- or C-terminal domains of high diversity and unknown functions. These range from predicted transmembrane regions, protein-protein interaction or regulatory domains, and, even, additional enzymatic folds (Figures 1A and S6A) and likely represent an additional layer of regulation, similar to eukaryotic ULP enzymes (Ronau et al., 2016).

The crystal structure of full-length SseL revealed an unannotated N-terminal domain, in which eight helices form a superhelical structure (Figure 6A). Comparison with known structures using the Dali server (Holm and Rosenström, 2010) indicated similarity with the VHS (VPS-27, Hrs, and STAM) domains of GGA1 (PDB: 1PY1, [He et al., 2003], Dali Z score of 5.0, RMSD 4.0 Å), and STAM1 (PDB: 3LDZ [Ren and Hurley, 2010], Dali

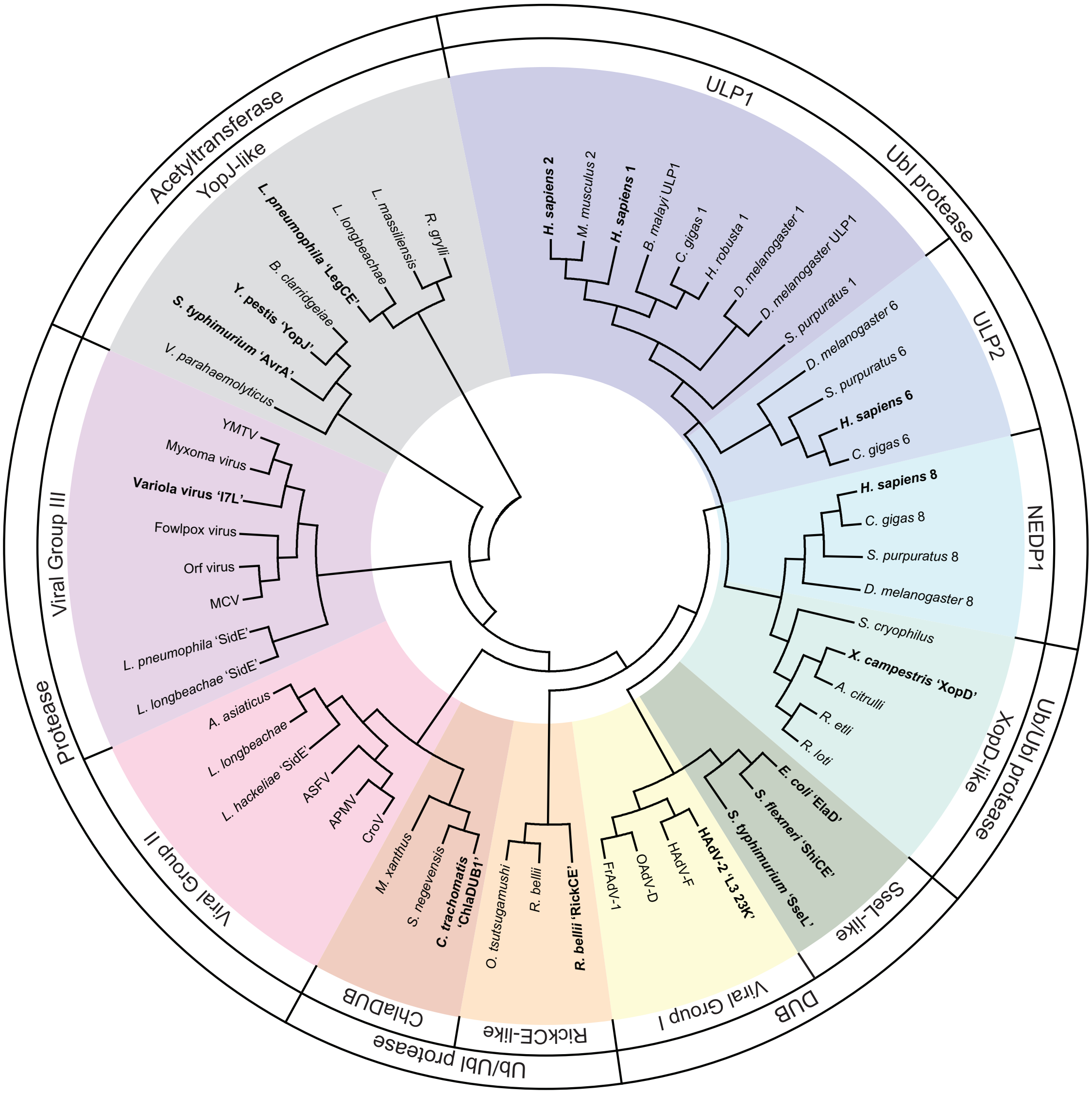

Figure 5. Bioinformatic Analysis of the CE Protease Clan

Structure-informed dendrogram for the CE clan, with representative members from eukaryotes, bacteria, and viruses. Clustered families are highlighted and labeled according to the archetypal examples. Eukaryotic family members are labeled with numbers corresponding to their SENP nomenclature. Characterized members are highlighted in bold. See also Figure S5.

Z score of 4.2, RMSD 3.8 Å) (Figure 6B). In eukaryotes, VHS domains are involved in vesicular trafficking, either through direct interaction with a phosphorylated receptor (e.g., GGA1 [Shiba et al., 2002]) or by binding Ub attached to cargo (e.g., STAM1 [Ren and Hurley, 2010]).

VHS domains, so far, are unknown to occur in bacteria, and the functional peptide- and Ub-binding interfaces differ in the SseL VHS domain. SseL did not interact with phosphorylated or unphosphorylated mannose-6-phosphate receptor (MPR) peptides (Figure S6B). In contrast, NMR titration experiments of the isolated SseL VHS domain revealed low micromolar interactions with the Ile36 and Ile44 hydrophobic patches of Ub (Figures 6C–6E and S6C). Identical Ub binding was also observed with an SseL construct including the CE fold (aa 24–340) (Figure S6D), and this was unaffected by mutations in the S1 Ub binding site or when a covalent SseL~Ub complex was used in the titration (Figures S6E and S6F), suggesting that the VHS domain constitutes the highest affinity Ub binding site.

Inspection of the SseL VHS structure identified two solvent-exposed hydrophobic patches centered on Leu46 and Trp105 as potential Ub-interacting surfaces (Figures 6F and S6G). Ub binding was unaffected with a SseL L46R mutant (Figure S6H), but abrogated in a SseL W105A mutant (Figure 6G). This Ub binding site on SseL is distinct from that on the STAM1 VHS domain and remote from the catalytic center (Figure S6I). The SseL W105A mutant had no effect on Ub chain hydrolysis for K63-linked di-, tri-, or tetra-Ub (Figures 6H and S6J). This

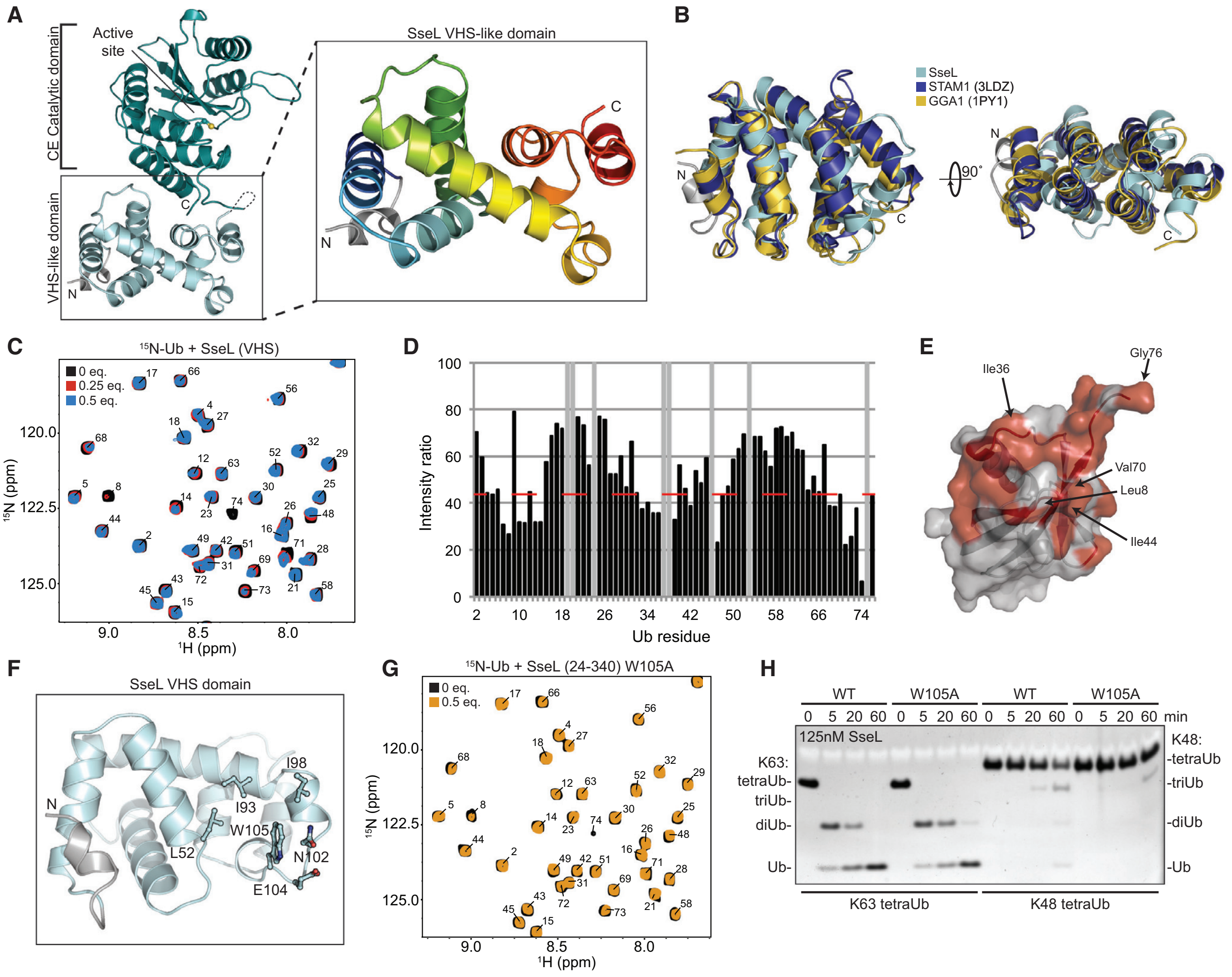

Figure 6. CE Catalytic Domains Are Fitted with Accessory Domains

(A) SseL crystal structure (active-site Cys in a ball-and-stick representation), focusing on the superhelical N-terminal VHS domain.

(B) Superposition of the SseL N-terminal domain (cyan) with the VHS domains of STAM1 (PDB: 3LDZ, dark blue), and GGA1 (PDB: 1PY1, orange).

(C) ^{1}H,^{15}N-HSQC TROSY spectra of 80 μM ^{15}N-labeled Ub alone (black) and in the presence of 0.25 (20 μM, red) or 0.5 (40 μM, blue) molar equivalents of the SseL VHS domain (see the Supplemental Experimental Procedures). Numbers refer to the assigned Ub residues.

(D) Calculated ratio in peak intensity observed between ^{15}N-labeled Ub alone and in the presence of 0.5 molar equivalent SseL VHS domain. A red dashed line marks the level of significance used in (E). Gray bars indicate prolines or missing resonances.

(E) Surface representation of the Ub structure (PDB: 1UBQ) with red painted surface corresponding to regions significantly affected by SseL VHS binding.

(F) Exposed hydrophobic patch within the SseL VHS domain; W105 was chosen for mutation.

(G) ^{1}H,^{15}N-HSQC TROSY spectra of 80 μM ^{15}N-labeled Ub alone (black) and in the presence of 0.5 molar equivalents of SseL (24–340) W105A (40 μM, orange).

(H) Time course assays monitoring cleavage of K63- and K48-linked tetraUb chains with the SseL VHS domain Ub-binding mutant.

See also Figure S6.

suggested that the added Ub binding site outside of the SseL catalytic domain does not contribute to the recognition or hydrolysis of longer Ub chains, but may instead serve alternative roles in regulating SseL during *Salmonella* infection.

The SseL VHS Domain Dictates Subcellular Localization of the Effector

Following translocation from the *Salmonella*-containing vacuole (SCV), SseL robustly localizes to the outer leaflet of the vacuolar membrane, as well as to *Salmonella*-induced filaments (Sifs) that emanate from the SCV (Rytkönen et al., 2007). To determine if this localization is determined by the Ub-binding activity of the VHS domain, we reintroduced HA-tagged versions of wild-type (SseL-2HA WT), S1 site mutant (SseL-2HA Y183A), and VHS mutant (SseL-2HA W105A) into the *Salmonella* Typhimurium Δ*sseL* strain to be expressed from a plasmid under the native promoter. HeLa cells were infected with the complemented strains for 16 hr, then fixed, and immune-labeled for

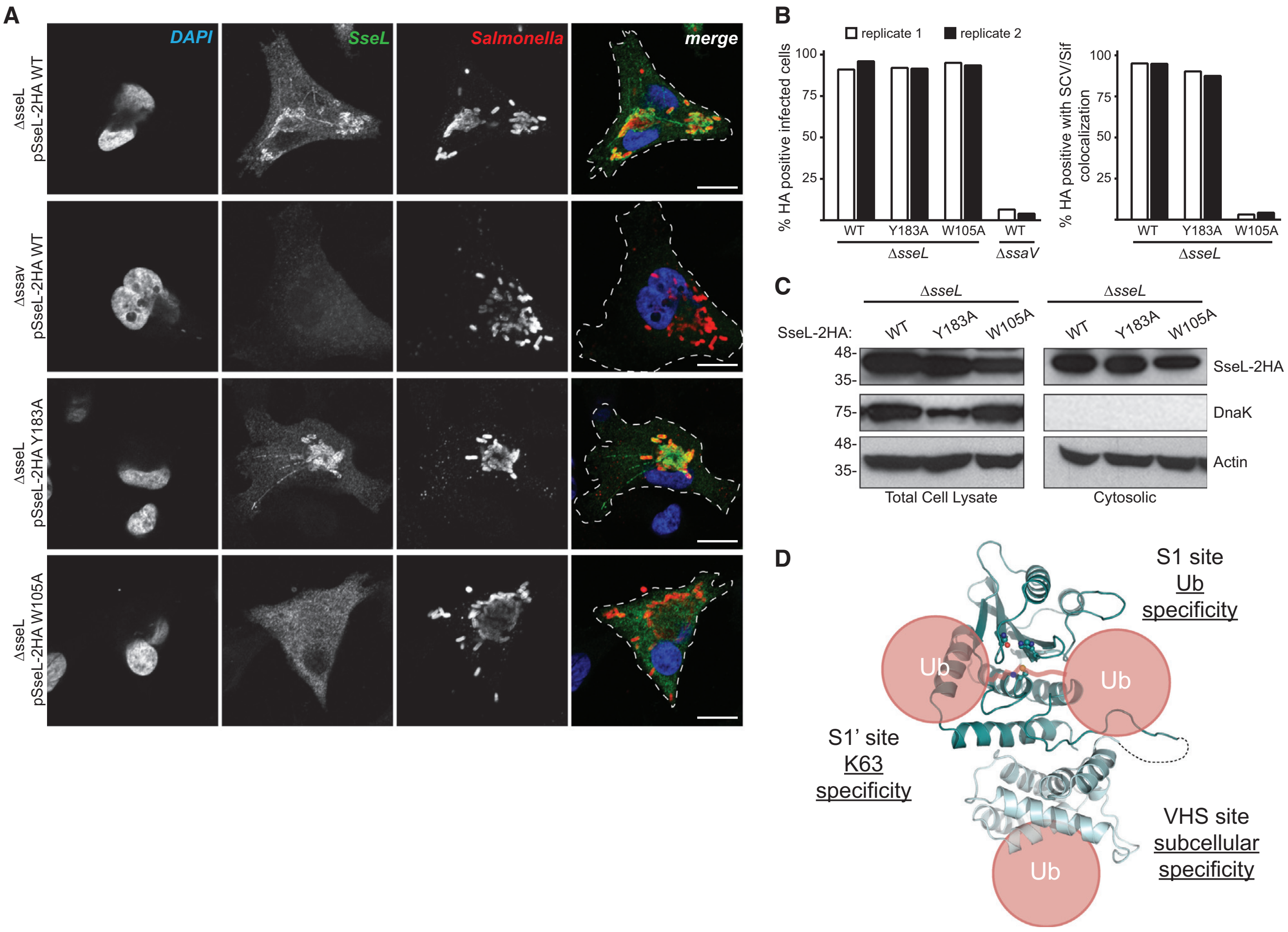

Figure 7. The SseL Accessory VHS Domain Dictates Subcellular Localization

(A) Representative images of HeLa cells infected with *S*. Typhimurium strains expressing 2HA-tagged wild-type or mutant SseL, in the *ΔsseL* or *ΔssaV* (SPI-2 T3SS null) background. Cells were immunostained with HA (SseL) and CSA-1 (*S*. Typhimurium), and DNA was stained with DAPI. White bar, 10 μM.

(B) Quantitation of (A). Infections were performed in duplicate (white and black bars). 200 cells from each replicate of each infection were assessed for SseL localization to SCV and Sifs.

(C) Cytosolic and total cell lysate fractions of infections performed as in (A), separated by SDS-PAGE, and immunoblotted for HA (SseL), DnaK, and Actin.

(D) Model demonstrating the multiple Ub binding modes exhibited by SseL and their effects on various levels of specificity.

See also Figure S7.

SseL-2HA and CSA-1 (a marker for bacteria). Wild-type SseL localized to SCVs and Sifs in a SPI-2 type 3 secretion system (T3SS)-dependent manner (compared to *ΔssaV* T3SS-deficient control), as expected (Figure 7A). The localization of S1 site mutant Y183A was indistinguishable from wild-type, indicating that Ub binding to the catalytic domain does not dictate subcellular localization (Figures 7A–7C and S7A). The W105A VHS mutant, however, showed a severe localization defect and was distributed diffusely throughout the host cytoplasm (Figures 7A–7C and S7A). Therefore, the N-terminal VHS domain of SseL serves to localize its catalytic DUB function through an additional Ub binding site (Figure 7D). Both ElaD and ShiCE encode similar N-terminal VHS domains and the Ub binding site is conserved (Figures 1A and S1A). Hence, we would predict that this subfamily of effectors has adopted analogous strategies of regulation.

DISCUSSION

During infection, bacteria secrete effector proteins, such as CE clan proteins, which inactivate inflammatory signaling cascades and promote bacterial growth. CE clan effectors were reported to be deSUMOylases, deubiquitinases, and, even, acetyltransferases, contrasting the specific and important roles of these enzymes in removal of a small subset of Ub-like modifications (SUMO and NEDD8) in eukaryotes. This ambiguity triggered the here presented comprehensive characterization of bacterial CE effectors, revealing dedicated DUBs and dedicated acetyltransferases, but also mixed Ub/Ubl proteases (Ub/NEDD8 and strikingly also Ub/SUMO). All this is performed by a single, highly adaptable catalytic fold. Complementary bioinformatic analysis based on structural data expands the family and reveals some of the functional

differences. For example, we have annotated and characterized the first DUB of *Shigella*, an activity that has been inconspicuously lacking considering its large repertoire of effector E3 ligases (Ashida et al., 2014). However, the functional variation observed among CE clan members is staggering, making some enzymatic features unpredictable, especially when mixed activities are suspected.

DUB activity in CE effectors has been suggested to exist in various bacteria, and while we now understand strategies for adaptation of Ub specificity in molecular detail, it is surprising that bacteria have adopted such distinct structural approaches to generate a Ub-specific S1 site. This suggests the convergent evolution of distinct bacterial DUBs. Based on our structural work, we identify three common regions of variability that, together, encode Ub/Ubl substrate specificity. In our minds, the most surprising insight is the mixed activity of XopD being a (tomato−) SUMO and Ub isopeptidase, since these modifiers are quite distinct and the capacity to efficiently recognize both substrates suggests a large evolutionary pressure to become a multifunctional protease. Interestingly, our structural analysis of XopD bound to either tSUMO or Ub demonstrates that XopD's flexibility in substrate recognition primarily arises from incorporation of an intrinsically disordered VR-1, which can be tailored to suit either binding event.

Moreover, we now understand the preference of most CE DUBs for K63-linked chains because of the presence of a defined, linkage-specificity-generating S1' site. This site on CE proteases was unanticipated and is unknown for the CE ULPs; whether the six SENP members in humans preferentially target distinct SUMO chains is unclear. Our mutagenesis data further show that the observed K63 specificity hinges on several key contacts, and changes in these can switch specificity. Hence, we would anticipate that CE DUBs could target other chain types, if advantageous for infection (see XopD; Figure 3D).

Finally, the CE folds of most effectors are accessorized with additional domains, and we have shown that a Ub-binding VHS domain in SseL (and by sequence similarity in ElaD and ShiCE) mediates localization of the effector. The VHS domains of ESCRT-0 components STAM and Hrs bind ubiquitinated cargo to direct the lysosomal maturation pathway (Ren and Hurley, 2010). Similarly, the VHS domain of SseL binds Ub (albeit via a distinct surface) and functions in localizing it to Sifs and to the cytosolic face of the SCV, where the catalytic CE domain can remove K63-linked polyUb. This could block recruitment of xenophagy adaptors, such as p62 (Mesquita et al., 2012), through general removal of K63-linked chains, or directly target specific host proteins, such as oxysterol-binding protein (OSBP) (Auweter et al., 2012). Furthermore, given that many of these accessory domains are unrelated across CE clan members (Figures 1A and S6A), the breadth of possibilities through which CE enzymatic activities may be regulated is likely extensive. Our study serves as a framework to further investigate this intriguing class of effectors and, in particular, highlights several interesting mechanisms by which bacterial effectors encode Ub/Ubl specificity.

EXPERIMENTAL PROCEDURES

Cloning, Expression, and Purification of the CE Effectors

Protein sequences were either cloned from bacterial or synthesized DNA or were received as gifts from colleagues. Constructs were generated as outlined in Figure 1A in the pOPIN-B or pOPIN-K vectors and purified using affinity, anion exchange, and size exclusion chromatography.

DUB Activity Assays

Qualitative DUB assays, suicide probe assays, TAMRA-based Ub/Ubl FP assays, and FlAsH-based diUb cleavage assays are described in detail in the Supplemental Experimental Procedures.

Protein Crystallization and Structure Determination

Crystallization conditions were optimized based on initial hits obtained from commercial screens in sitting-drop format. Structure determination was performed using either single wavelength anomalous dispersion (SAD) experimental phasing of selenomethionine derivatives or molecular replacement (see Table 1).

Construction of CE Dendrogram

Novel and existing CE structures from bacteria, eukaryotes, and viruses were used to create a "seed" structure-based sequence alignment, which was iteratively refined and expanded on with additional examples. This alignment was used to identify more divergent examples of the CE fold and to construct a dendrogram of the CE clan using the UPGMA method.

NMR Spectroscopy

NMR titration spectra of uniformly labeled ^{15}N-Ub were recorded at 25°C on either a Bruker Avance III 600 MHz or an Avance2+ 700 MHz spectrometer, equipped with cryogenic triple-resonance TCI probes. Data processing and analysis were performed in Topspin (Bruker) and NMRView (One Moon Scientific).

S. Typhimurium Infection Assays

Infection assays were performed as described previously (Rytkönen et al., 2007), with changes as discussed in the Supplemental Experimental Procedures.

ACCESSION NUMBERS

The accession numbers for the coordinates and structure factors for SseL, ChlaDUB1, RickCE, XopD~Ub, and XopD~tSUMO reported in this paper have been uploaded to PDB: 5HAF, 5HAG, 5HAM, 5JP3, and 5JP1.

SUPPLEMENTAL INFORMATION

Supplemental Information includes Supplemental Experimental Procedures and seven figures and can be found with this article online at http://dx.doi.org/10.1016/j.molcel.2016.06.015.

AUTHOR CONTRIBUTIONS

D.K. conceived the project, and together, J.N.P. and D.K. designed the project. J.N.P. performed all of the biochemical and structural experiments, with input from D.K. B.S. performed the bioinformatics analysis. C.H.D. performed the *Salmonella* infection experiments, with input from D.W.H. P.P.G. and H.O. provided Ub/Ubl reagents. J.N.P. and D.K. wrote the manuscript, with input from all authors.

ACKNOWLEDGMENTS

We would like to thank R. Mittal (MRC Laboratory of Molecular Biology), R. Hay (University of Dundee), B. Schulman (St. Jude Children's Research Hospital), and A. Canto-Pastor and D. Baulcombe (University of Cambridge) for providing reagents, and the beamline staff at ESRF ID23-1 and DLS I03, I04,

and I04-1 for their assistance. Access to DLS was supported, in part, by the EU FP7 infrastructure grant BIOSTRUCT-X (contract no. 283570). The graphical abstract uses images adapted from Servier Medical Art. We also thank members of the D.K. lab for reagents and advice and K. Swatek for critical comments on the manuscript. Work in the D.K. lab was funded by the Medical Research Council (U105192732), the European Research Council (309756), and the Lister Institute for Preventive Medicine. J.N.P was supported by an EMBO Long-term Fellowship. Work in the H.O. lab was supported by a Netherlands Organization for Scientific Research VICI grant (724.013.002) and the European Research Council (281699). C.H.D. and D.W.H. were funded by a Medical Research Council grant (WMNM_P45464) (to D.W.H.). B.S. was funded by the Medical Research Council (U105185859). D.K. and H.O. are part of the DUB Alliance that includes Cancer Research Technology and FORMA Therapeutics. H.O. is founder and shareholder of the company UbiQ that markets reagents in the ubiquitin field.

Received: April 7, 2016
Revised: May 2, 2016
Accepted: June 8, 2016
Published: July 14, 2016

REFERENCES

Ashida, H., Kim, M., and Sasakawa, C. (2014). Exploitation of the host ubiquitin system by human bacterial pathogens. Nat. Rev. Microbiol. *12*, 399–413.

Auweter, S.D., Yu, H.B., Arena, E.T., Guttman, J.A., and Finlay, B.B. (2012). Oxysterol-binding protein (OSBP) enhances replication of intracellular *Salmonella* and binds the *Salmonella* SPI-2 effector SseL via its N-terminus. Microbes Infect. *14*, 148–154.

Balakirev, M.Y., Jaquinod, M., Haas, A.L., and Chroboczek, J. (2002). Deubiquitinating function of adenovirus proteinase. J. Virol. *76*, 6323–6331.

Basters, A., Geurink, P.P., El Oualid, F., Ketscher, L., Casutt, M.S., Krause, E., Ovaa, H., Knobeloch, K.P., and Fritz, G. (2014). Molecular characterization of ubiquitin-specific protease 18 reveals substrate specificity for interferon-stimulated gene 15. FEBS J. *281*, 1918–1928.

Byrd, C.M., Bolken, T.C., and Hruby, D.E. (2003). Molecular dissection of the vaccinia virus I7L core protein proteinase. J. Virol. *77*, 11279–11283.

Catic, A., Misaghi, S., Korbel, G.A., and Ploegh, H.L. (2007). ElaD, a deubiquitinating protease expressed by *E. coli*. PLoS ONE *2*, e381.

Chosed, R., Tomchick, D.R., Brautigam, C.A., Mukherjee, S., Negi, V.S., Machius, M., and Orth, K. (2007). Structural analysis of *Xanthomonas* XopD provides insights into substrate specificity of ubiquitin-like protein proteases. J. Biol. Chem. *282*, 6773–6782.

Clague, M.J., Barsukov, I., Coulson, J.M., Liu, H., Rigden, D.J., and Urbé, S. (2013). Deubiquitylases from genes to organism. Physiol. Rev. *93*, 1289–1315.

Corn, J.E., and Vucic, D. (2014). Ubiquitin in inflammation: the right linkage makes all the difference. Nat. Struct. Mol. Biol. *21*, 297–300.

Ding, J., McGrath, W.J., Sweet, R.M., and Mangel, W.F. (1996). Crystal structure of the human adenovirus proteinase with its 11 amino acid cofactor. EMBO J. *15*, 1778–1783.

Dosztányi, Z., Csizmók, V., Tompa, P., and Simon, I. (2005). The pairwise energy content estimated from amino acid composition discriminates between folded and intrinsically unstructured proteins. J. Mol. Biol. *347*, 827–839.

Ekkebus, R., van Kasteren, S.I., Kulathu, Y., Scholten, A., Berlin, I., Geurink, P.P., de Jong, A., Goerdayal, S., Neefjes, J., Heck, A.J.R., et al. (2013). On terminal alkynes that can react with active-site cysteine nucleophiles in proteases. J. Am. Chem. Soc. *135*, 2867–2870.

Figueira, R., and Holden, D.W. (2012). Functions of the *Salmonella* pathogenicity island 2 (SPI-2) type III secretion system effectors. Microbiology *158*, 1147–1161.

Geurink, P.P., El Oualid, F., Jonker, A., Hameed, D.S., and Ovaa, H. (2012). A general chemical ligation approach towards isopeptide-linked ubiquitin and ubiquitin-like assay reagents. ChemBioChem *13*, 293–297.

He, X., Zhu, G., Koelsch, G., Rodgers, K.K., Zhang, X.C., and Tang, J. (2003). Biochemical and structural characterization of the interaction of memapsin 2 (beta-secretase) cytosolic domain with the VHS domain of GGA proteins. Biochemistry *42*, 12174–12180.

Holm, L., and Rosenström, P. (2010). Dali server: conservation mapping in 3D. Nucleic Acids Res. *38*, W545–W549.

Jones, R.M., Wu, H., Wentworth, C., Luo, L., Collier-Hyams, L., and Neish, A.S. (2008). *Salmonella* AvrA coordinates suppression of host immune and apoptotic defenses via JNK pathway blockade. Cell Host Microbe *3*, 233–244.

Keusekotten, K., Elliott, P.R., Glockner, L., Fiil, B.K., Damgaard, R.B., Kulathu, Y., Wauer, T., Hospenthal, M.K., Gyrd-Hansen, M., Krappmann, D., et al. (2013). OTULIN antagonizes LUBAC signaling by specifically hydrolyzing Met1-linked polyubiquitin. Cell *153*, 1312–1326.

Kim, J.G., Taylor, K.W., Hotson, A., Keegan, M., Schmelz, E.A., and Mudgett, M.B. (2008). XopD SUMO protease affects host transcription, promotes pathogen growth, and delays symptom development in *xanthomonas*-infected tomato leaves. Plant Cell *20*, 1915–1929.

Liu, X., Wang, Q., Chen, W., and Wang, C. (2013). Dynamic regulation of innate immunity by ubiquitin and ubiquitin-like proteins. Cytokine Growth Factor Rev. *24*, 559–570.

Mesquita, F.S., Thomas, M., Sachse, M., Santos, A.J.M., Figueira, R., and Holden, D.W. (2012). The *Salmonella* deubiquitinase SseL inhibits selective autophagy of cytosolic aggregates. PLoS Pathog. *8*, e1002743.

Mevissen, T.E.T., Hospenthal, M.K., Geurink, P.P., Elliott, P.R., Akutsu, M., Arnaudo, N., Ekkebus, R., Kulathu, Y., Wauer, T., El Oualid, F., et al. (2013). OTU deubiquitinases reveal mechanisms of linkage specificity and enable ubiquitin chain restriction analysis. Cell *154*, 169–184.

Misaghi, S., Balsara, Z.R., Catic, A., Spooner, E., Ploegh, H.L., and Starnbach, M.N. (2006). *Chlamydia trachomatis*-derived deubiquitinating enzymes in mammalian cells during infection. Mol. Microbiol. *61*, 142–150.

Mittal, R., Peak-Chew, S.Y., and McMahon, H.T. (2006). Acetylation of MEK2 and I κ B kinase (IKK) activation loop residues by YopJ inhibits signaling. Proc. Natl. Acad. Sci. USA *103*, 18574–18579.

Mittal, R., Peak-Chew, S.Y., Sade, R.S., Vallis, Y., and McMahon, H.T. (2010). The acetyltransferase activity of the bacterial toxin YopJ of *Yersinia* is activated by eukaryotic host cell inositol hexakisphosphate. J. Biol. Chem. *285*, 19927–19934.

Mukherjee, S., Keitany, G., Li, Y., Wang, Y., Ball, H.L., Goldsmith, E.J., and Orth, K. (2006). *Yersinia* YopJ acetylates and inhibits kinase activation by blocking phosphorylation. Science *312*, 1211–1214.

Orth, K., Xu, Z., Mudgett, M.B., Bao, Z.Q., Palmer, L.E., Bliska, J.B., Mangel, W.F., Staskawicz, B., and Dixon, J.E. (2000). Disruption of signaling by *Yersinia* effector YopJ, a ubiquitin-like protein protease. Science *290*, 1594–1597.

Radoshevich, L., Impens, F., Ribet, D., Quereda, J.J., Nam Tham, T., Nahori, M.A., Bierne, H., Dussurget, O., Pizarro-Cerdá, J., Knobeloch, K.P., and Cossart, P. (2015). ISG15 counteracts Listeria monocytogenes infection. eLife *4*, 06848.

Ren, X., and Hurley, J.H. (2010). VHS domains of ESCRT-0 cooperate in high-avidity binding to polyubiquitinated cargo. EMBO J. *29*, 1045–1054.

Reverter, D., and Lima, C.D. (2004). A basis for SUMO protease specificity provided by analysis of human Senp2 and a Senp2-SUMO complex. Structure *12*, 1519–1531.

Reverter, D., Wu, K., Erdene, T.G., Pan, Z.Q., Wilkinson, K.D., and Lima, C.D. (2005). Structure of a complex between Nedd8 and the Ulp/Senp protease family member Den1. J. Mol. Biol. *345*, 141–151.

Ronau, J.A., Beckmann, J.F., and Hochstrasser, M. (2016). Substrate specificity of the ubiquitin and Ubl proteases. Cell Res. *26*, 441–456.

Rytkönen, A., Poh, J., Garmendia, J., Boyle, C., Thompson, A., Liu, M., Freemont, P., Hinton, J.C.D., and Holden, D.W. (2007). SseL, a *Salmonella* deubiquitinase required for macrophage killing and virulence. Proc. Natl. Acad. Sci. USA *104*, 3502–3507.

Salomon, D., and Orth, K. (2013). What pathogens have taught us about post-translational modifications. Cell Host Microbe *14*, 269–279.

Sheedlo, M.J., Qiu, J., Tan, Y., Paul, L.N., Luo, Z.-Q., and Das, C. (2015). Structural basis of substrate recognition by a bacterial deubiquitinase important for dynamics of phagosome ubiquitination. Proc. Natl. Acad. Sci. USA *112*, 15090–15095.

Shen, L.N., Liu, H., Dong, C., Xirodimas, D., Naismith, J.H., and Hay, R.T. (2005). Structural basis of NEDD8 ubiquitin discrimination by the deNEDDylating enzyme NEDP1. EMBO J. *24*, 1341–1351.

Shiba, T., Takatsu, H., Nogi, T., Matsugaki, N., Kawasaki, M., Igarashi, N., Suzuki, M., Kato, R., Earnest, T., Nakayama, K., and Wakatsuki, S. (2002). Structural basis for recognition of acidic-cluster dileucine sequence by GGA1. Nature *415*, 937–941.

Swatek, K.N., and Komander, D. (2016). Ubiquitin modifications. Cell Res. *26*, 399–422.

van der Veen, A.G., and Ploegh, H.L. (2012). Ubiquitin-like proteins. Annu. Rev. Biochem. *81*, 323–357.

Ye, Z., Petrof, E.O., Boone, D., Claud, E.C., and Sun, J. (2007). *Salmonella* effector AvrA regulation of colonic epithelial cell inflammation by deubiquitination. Am. J. Pathol. *171*, 882–892.

Ye, Y., Akutsu, M., Reyes-Turcu, F., Enchev, R.I., Wilkinson, K.D., and Komander, D. (2011). Polyubiquitin binding and cross-reactivity in the USP domain deubiquitinase USP21. EMBO Rep. *12*, 350–357.

Zhou, H., Monack, D.M., Kayagaki, N., Wertz, I., Yin, J., Wolf, B., and Dixit, V.M. (2005). *Yersinia* virulence factor YopJ acts as a deubiquitinase to inhibit NF-κ B activation. J. Exp. Med. *202*, 1327–1332.

Molecular Cell, Volume *63*

Supplemental Information

The Molecular Basis for Ubiquitin and Ubiquitin-like Specificities in Bacterial Effector Proteases

Jonathan N. Pruneda, Charlotte H. Durkin, Paul P. Geurink, Huib Ovaa, Balaji Santhanam, David W. Holden, and David Komander

Figure S1: CE effector proteins demonstrate mixed proteolytic activities

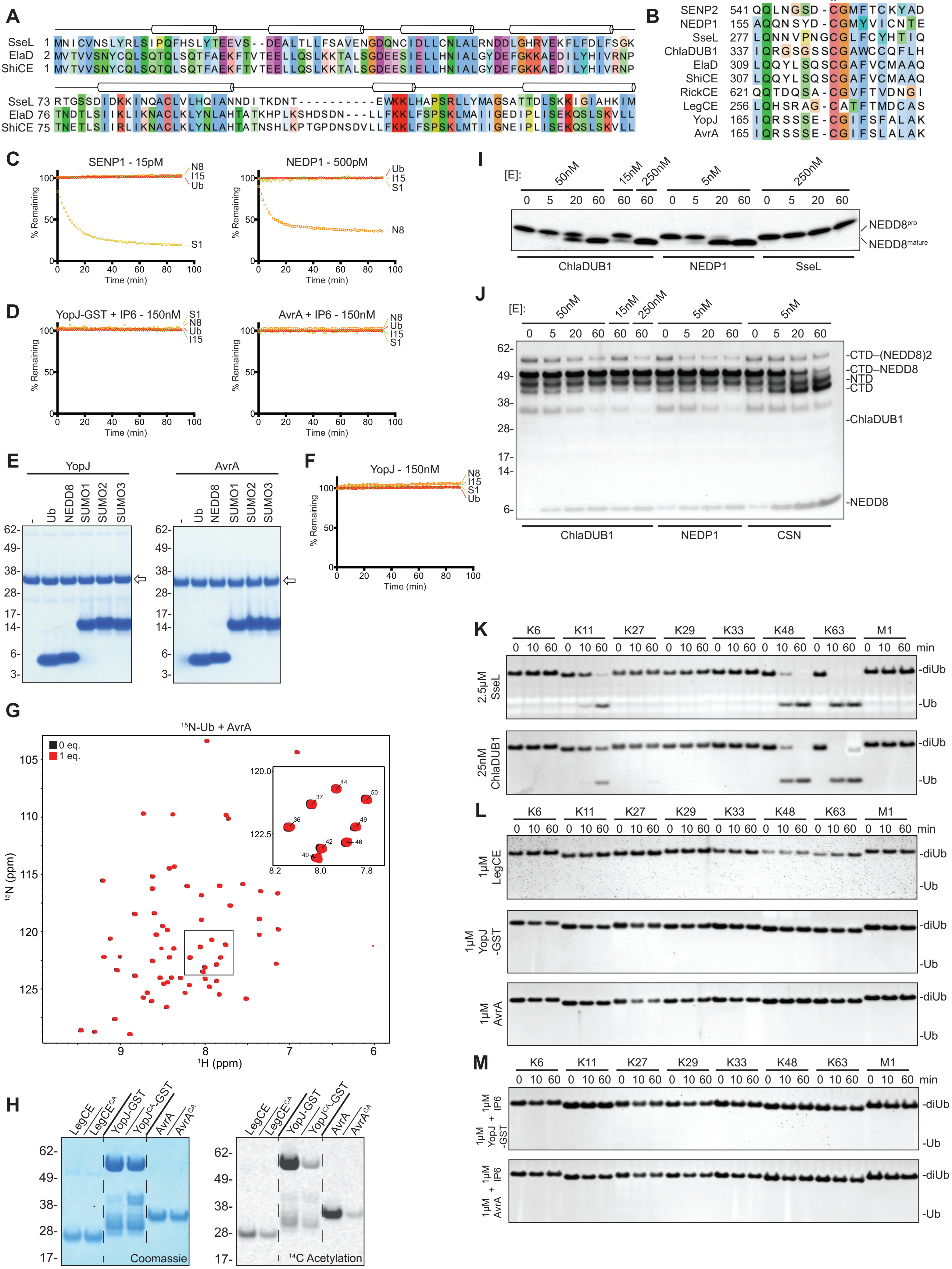

Figure S1: CE effector proteins demonstrate mixed proteolytic activities; related to Figure 1
A) Sequence alignment for the N-terminal accessory domains of SseL, ElaD, and ShiCE. Agreement in secondary structure prediction calculated for each individual sequence is shown above. **B)** Sequence alignment used to identify the putative catalytic Cys in bacterial CE enzymes (marked by an asterisk) by similarity to eukaryotic CE examples. **C)** Normalized FP measured as a function of time, as in **Figure 1E**, following addition of the well-studied SUMO-specific human SENP1 and NEDD8-specific human NEDP1. **D)** As in **C** for YopJ and AvrA in the presence of excess activator IP6. **E)** Suicide probe reaction, as in **Figure 1C**, monitoring reactivity of untagged YopJ and AvrA toward the indicated Ub/Ubl probes following 1 h incubation at room temperature (propargylamine-derived probes). **F)** As in **C** for untagged YopJ. **G)** ^{1}H,^{15}N-HSQC TROSY spectra of 80μM ^{15}N-labeled Ub alone (black) and in the presence of 1 molar equivalent of AvrA (80μM, red). Inset, enlarged region of the spectral overlay with Ub resonance assignments labeled. **H)** Autoradiography monitoring isotope incorporation following a 2 h incubation of each protein with ^{14}C-labeled Acetyl-CoA in the presence of IP6. Compare signal in wild-type over inactive mutant background (see **Supplemental Experimental Procedures**). **I)** Time course monitoring cleavage of pro-NEDD8 (81 aa) to the mature form (76 aa), for the dual-specific ChlaDUB1, NEDD8-specific NEDP1, and Ub-specific SseL. This demonstrates that, like other CE proteases, ChlaDUB1 possesses both peptidase and isopeptidase activities. **J)** Time course monitoring cleavage of NEDDylated Cul1 C-terminal domain (CTD, modified at Lys720) in the presence of ChlaDUB1, and NEDP1 or COP9 signalosome (CSN) as negative and positive controls, respectively. Although ChlaDUB1 and NEDP1 can remove some NEDD8 modifications (e.g., CTD-(NEDD8)2), only the specialized CSN protease can remove the regulatory NEDD8 modification from Cul1 (CTD-NEDD8). **K)** Linkage specificity analysis for all diUb substrates, as in **Figure 1F**, for SseL and ChlaDUB1 at 10-fold higher enzyme concentration. **L)** Linkage specificity analysis for all diUb substrates, as in **Figure 1F**, for LegCE, YopJ, and AvrA. **M)** As in **L** for YopJ and AvrA in the presence of activator IP6.

Figure S2: Structural analysis of bacterial CE deubiquitinases

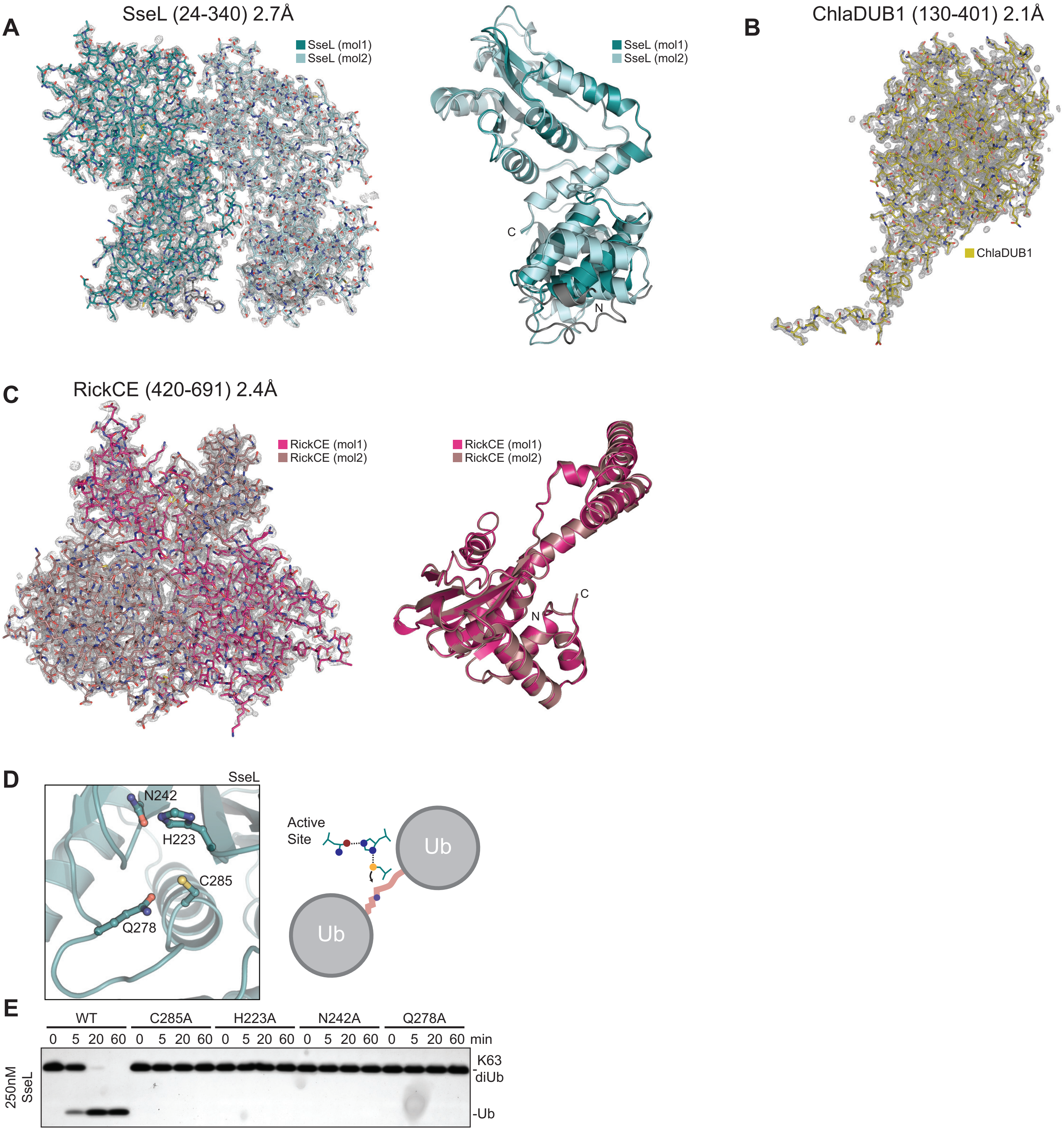

Figure S2: Structural analysis of bacterial CE deubiquitinases; related to Figure 2, Table 1
A) Full asymmetric unit (ASU) of the 2.7Å SseL (24-340) crystal structure showing a 2|Fo|-|Fc| electron density map contoured at 1σ, and superposition of the two copies in the ASU. Crystallization of this construct depended upon an N-terminal His-tag, which makes crystal contacts in one molecule and forms a short helix against the N-terminal domain in another. Most likely resulting from these crystal contacts at the N-terminus, the two molecules in the ASU show deviation within the N-terminal domain (1.99Å Cα RMSD for residues 24-136), but overlay well in the catalytic domain (0.39Å Cα RMSD for residues 154-340). SseL is colored in shades of teal, ordered regions of the His-tag are colored in grey. **B)** Full ASU of the 2.1Å ChlaDUB1 (130-401) structure showing 2|Fo|-|Fc| electron density contoured at 1σ. The first ~20 residues form a highly extended structure via crystal contacts. **C)** Full ASU of the 2.4Å RickCE (420-691) crystal structure, along with a 2|Fo|-|Fc| electron density map contoured at 1σ, and an alignment of the two copies in the ASU. The two identical molecules in the ASU (0.27Å Cα RMSD) are interlaced by a large insertion forming a helical arm. **D)** Active site Cys, His, Asn, and Gln of SseL (teal) are shown. **E)** K63 diUb cleavage assay using wild-type and catalytic mutants of SseL.

Figure S3: Molecular analysis of XopD Ub/Ubl specificity

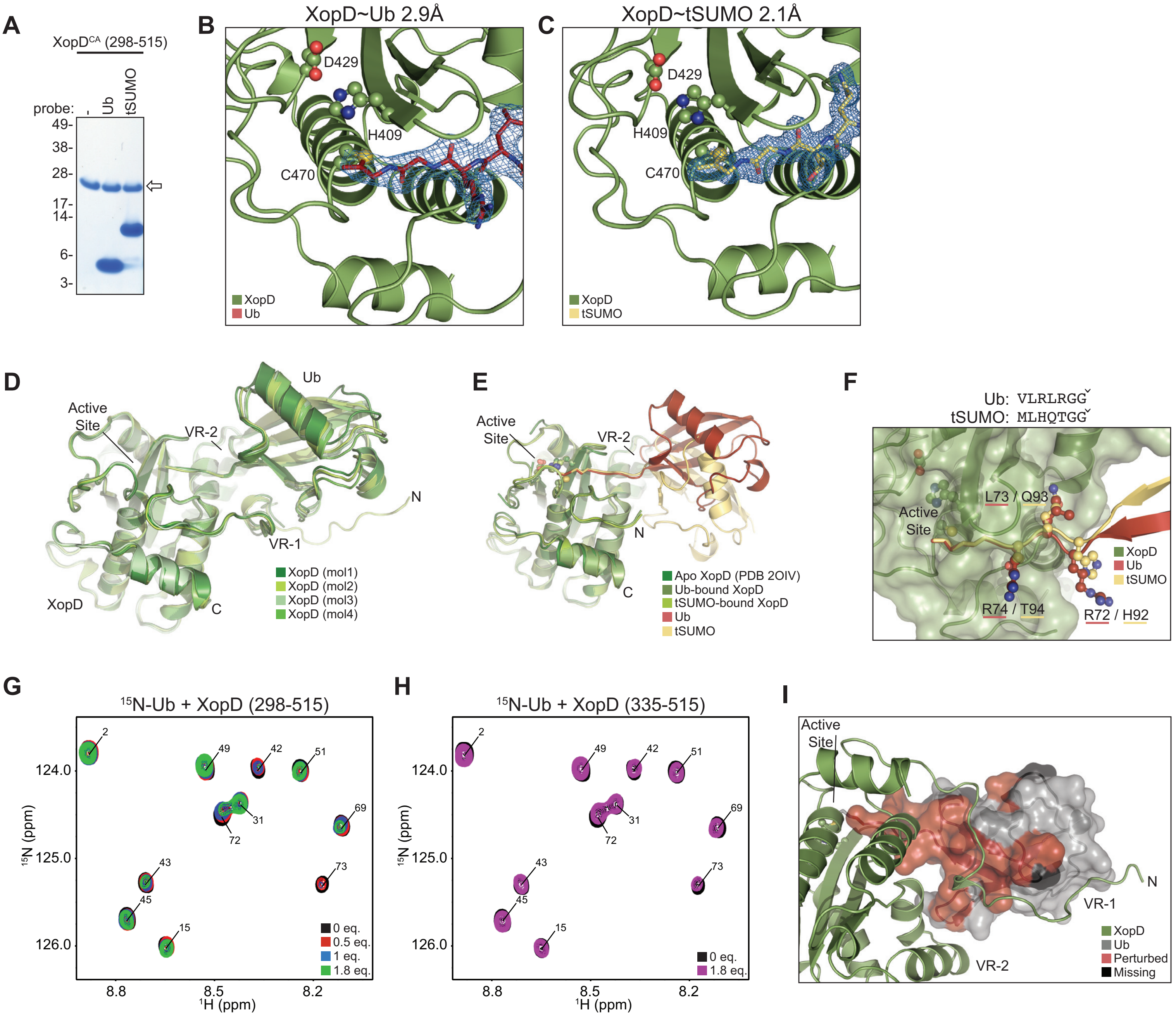

Figure S3: Molecular analysis of XopD Ub/Ubl specificity; related to Figure 3, Table 1

A) Catalytically inactive XopD +VR-1 C470A tested against the Ub and tSUMO suicide probes following a 1 h incubation at room temperature (propargylamine-derived probes). Open arrow, unmodified. **B)** Close-up of the XopD~Ub covalent linkage, showing XopD catalytic triad residues and 2|Fo|-|Fc| electron density of the Ub C-terminus, contoured at 1σ. **C)** As in **B**, for the XopD~tSUMO complex. **D)** Superposition of the four XopD~Ub molecules present in the ASU. **E)** Superposition of XopD from its apo (PDB 2OIV), Ub-bound, and tSUMO-bound structures. VR-1 is not shown for clarity. **F)** Close-up of the tract leading into the XopD active site. C-terminal residues for Ub and tSUMO are shown above, and their positions within the XopD-bound structures is depicted below. **G)** ^{1}H,^{15}N-HSQC TROSY spectra of 80μM ^{15}N-labeled Ub alone (black) and in the presence of 0.5 (40μM, red), 1 (80μM, blue), or 1.8 (144μM, green) molar equivalents of XopD +VR-1 (298-515). **H)** As in **G**, for 1.8 (144μM, magenta) molar equivalents of XopD ΔVR-1 (335-515). **I)** Resonances significantly perturbed in **G** by either line broadening or chemical shift perturbation mapped onto the XopD~Ub crystal structure (red). Prolines and other resonances missing from the NMR spectrum are shown in black.

Figure S4: Structure-based manipulation of CE DUB activities and specificities

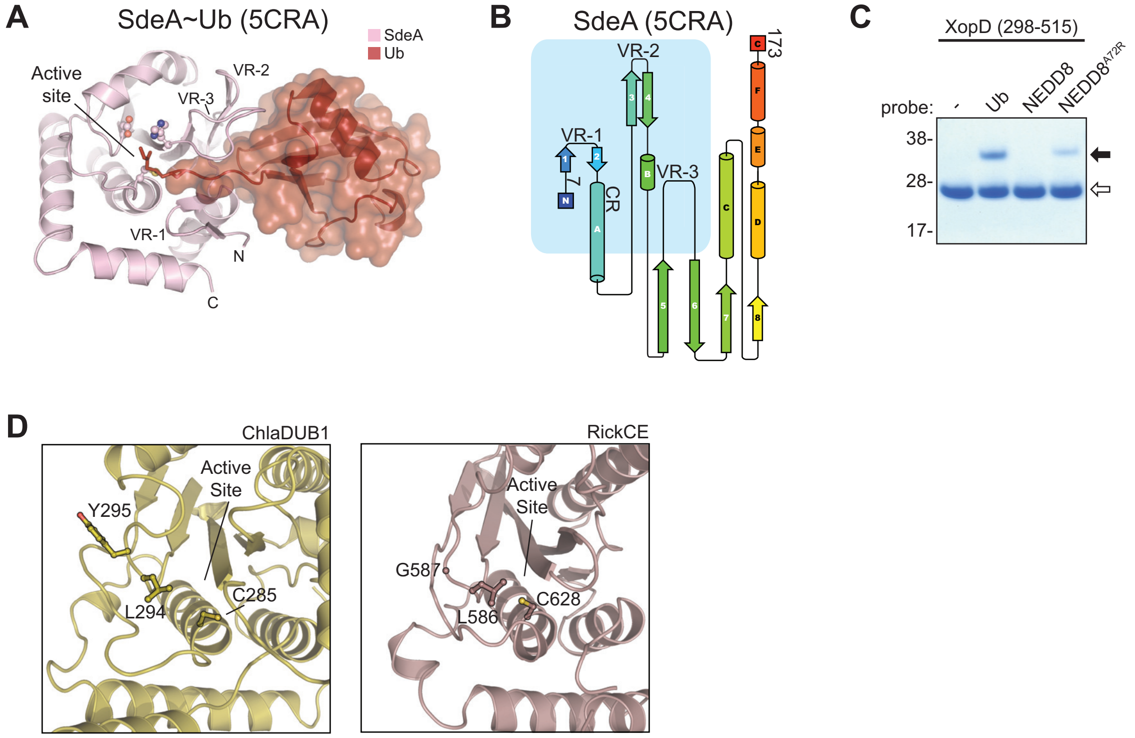

Figure S4: Structure-based manipulation of CE DUB activities and specificities; related to Figure 4
A) Cartoon representation of SidE family member SdeA in a covalent complex with Ub (PDB 5CRA). **B)** Topology diagram, as in **Figure 2E**, for the SdeA crystal structure (PDB 5CRA). **C)** Suicide probe assay showing XopD +VR-1 (298-335) reaction with Ub, NEDD8, and NEDD8 A72R probes at room temperature for 1 h (chloroethylamine-derived probes) Open arrow, unmodified; closed arrow, modified. **D)** Close-up of the conserved hydrophobic S1' sites of ChlaDUB1 and RickCE.

Figure S5: Bioinformatic analysis of the CE protease clan

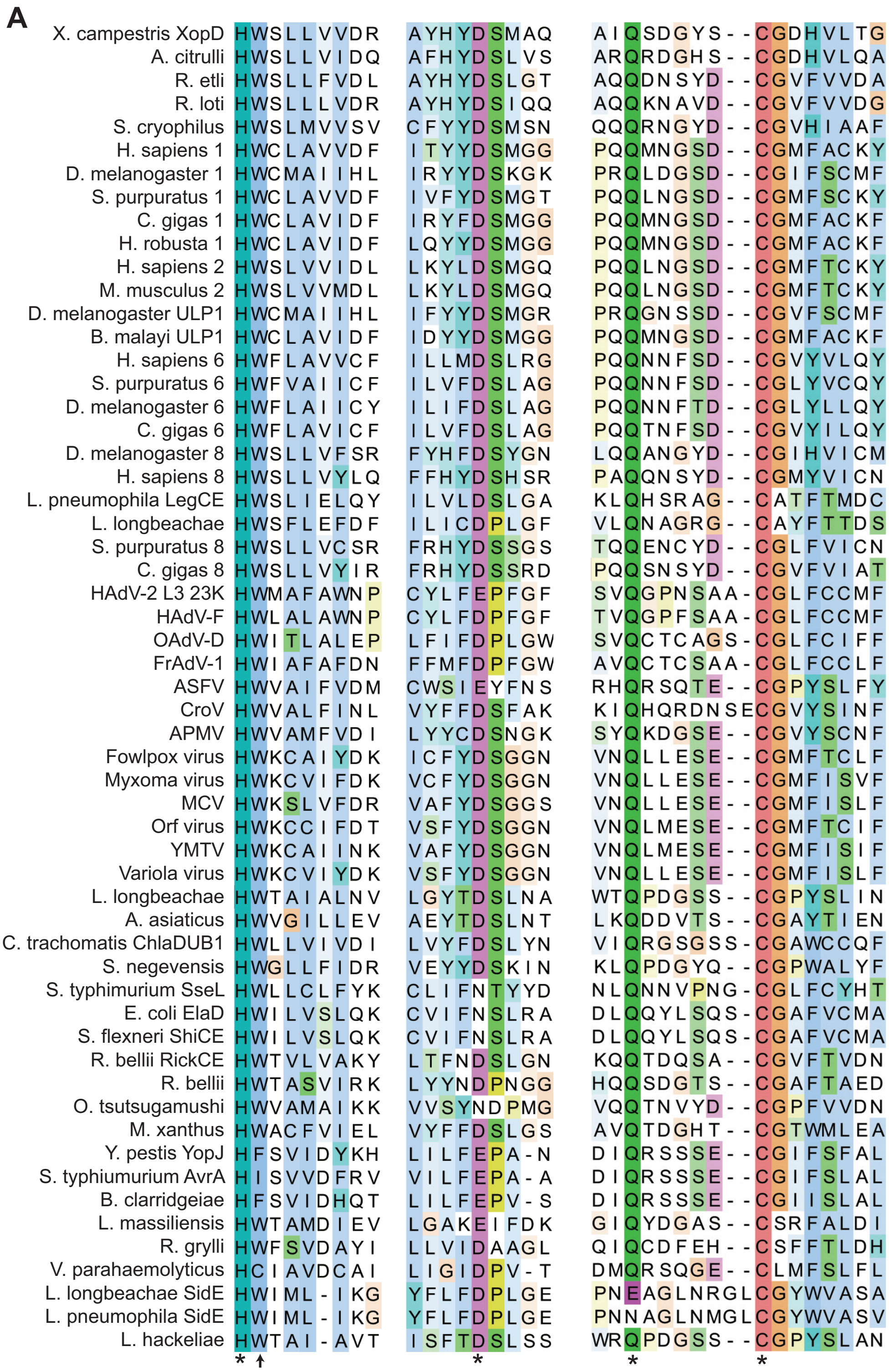

Figure S5: Bioinformatic analysis of the CE protease clan; related to Figure 5

A) Structure-guided sequence alignment of representative members from the CE clan, used to construct the dendrogram shown in **Figure 5**. For clarity, only regions immediately surrounding the catalytic center are shown. Coloring is based on similarity. One conserved Trp that is typically lacking in dedicated acetyltransferases is marked with an arrow. Catalytic residues are marked below with an asterisk.

Figure S6: CE catalytic domains are fitted with accessory domains

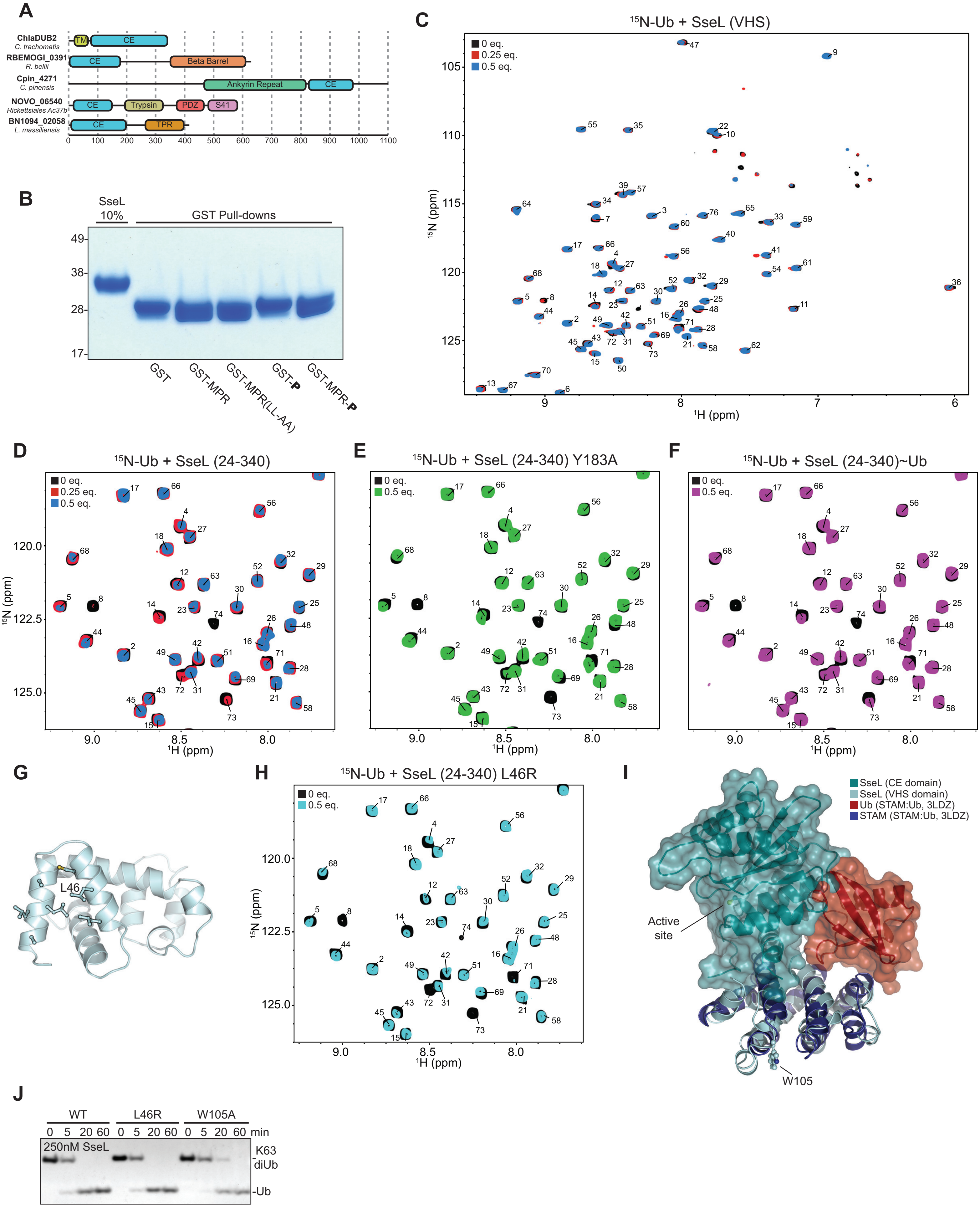

Figure S6: CE catalytic domains are fitted with accessory domains; related to Figure 6
A) Domain annotation of select CE enzymes from bacteria. TM, transmembrane helix; PDZ, PDZ protein interaction domain; S41, S41 peptidase domain; TPR, tetratricopeptide repeat. **B)** GST pull-down assay testing interaction of SseL to the cation independent mannose-6-phosphate receptor (CI-MPR) C-terminal peptide, as well as its LL-AA double mutant form and CK2-phosphorylated form. **C)** Full spectrum for the NMR titration shown in **Figure 6C**. 80 μM ^{15}N-labeled Ub alone (black) and in the presence of 0.25 (20μM, red) or 0.5 (40μM, blue) molar equivalents of SseL VHS domain. Line broadening effects suggest an interaction in the low-to-mid micromolar range. **D)** ^{1}H,^{15}N-HSQC TROSY titration experiment showing 80 μM ^{15}N-labeled Ub alone (black) and in the presence of 0.25 (20 μM, red) or 0.5 (40 μM, blue) molar equivalents of SseL (24-340). **E)** As in **C**, showing 0.5 molar addition of the S1 site Y183A mutant (green). **F)** As in **C**, showing 0.5 molar addition of the covalently-linked SseL~Ub complex (magenta). **G)** Exposed hydrophobic patch within the SseL VHS domain; Leu46 was chosen for mutation. **H)** ^{1}H,^{15}N-HSQC TROSY spectrum of 80 μM ^{15}N-labeled Ub alone (black) and in the presence of 0.5 molar equivalents of SseL (24-340) L46R (40μM, cyan). **I)** Overlay of the SseL catalytic and VHS domains (teal and cyan, respectively) with the STAM:Ub crystal structure (PDB 3LDZ, blue and red, respectively). As a conventional VHS:Ub interaction would clash with the position of the SseL catalytic domain, an unconventional site centered around Trp105 is utilized. **J)** Time course assays monitoring cleavage of K63-linked diUb with the SseL VHS domain mutants.

Figure S7: SseL accessory VHS domain dictates subcellular localization

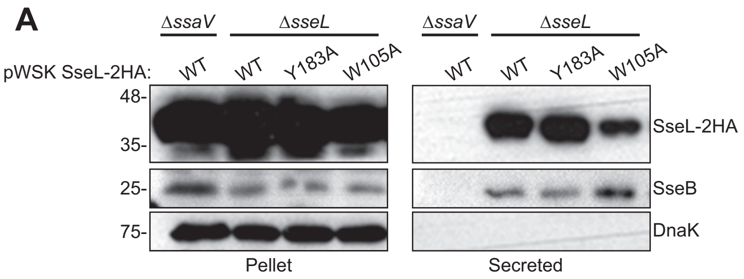

Figure S7: SseL accessory VHS domain dictates subcellular localization; related to Figure 7
A) Induced SPI-2 T3SS secretion of cultured *S.* Typhimurium strains expressing 2HA-tagged wild-type or mutant SseL, in the Δ*sseL* or Δ*ssaV* (SPI-2 T3SS null) background. Secreted and pellet fractions were separated by SDS-PAGE and immunoblotted for HA (SseL), SseB (SPI-2 T3SS translocon protein), and DnaK (intracellular control protein).

Supplemental Experimental Procedures:

Cloning and molecular biology

SseL, ElaD, and LegCE were cloned from the genomic DNA of *Salmonella* Typhimurium (strain LT2), *Escherichia coli* (serotype O157:H7), and *Legionella pneumophila* (strain Philadelphia, kind gift from C. Buchrieser, Institut Pasteur) using Phusion DNA polymerase (NEB). Genes encoding *Chlamydia trachomatis* ChlaDUB1, *Shigella flexneri* ShiCE, *Rickettsia bellii* RickCE, and *Xanthomonas campestris* XopD were obtained by gene synthesis (Life Technologies) and subcloned using KOD polymerase (Novagen). Amplified products were cloned into pOPIN vectors (Berrow et al., 2007) using the In-Fusion HD system (Clontech). Mutagenesis was performed using the QuikChange protocol. Constructs for YopJ in pMW and AvrA in pGEX6P-1 were kind gifts from R. Mittal (MRC LMB Cambridge). Constructs for SENP1 and NEDP1 in pHISTEV were kinds gifts from R. Hay (University of Dundee). tSUMO was amplified from a *Solanum lycopersicum* cDNA preparation (kind gift from A. Canto-Pastor and D. Baulcombe, University of Cambridge) and ligated into pTXB1 with conventional methods.

Protein expression and purification

SseL (24-340), ChlaDUB1 (130-401), ElaD (2-407), ShiCE (2-405), RickCE (378-691 or 420-691), LegCE (141-360), and XopD (298-515 or 335-515) were expressed from the pOPIN-B vector (N-terminal His-3C), AvrA (1-288) from the pGEX6P-1 vector (N-terminal GST-3C), YopJ (1-288) from the pMW vector (C-terminal 3C-GST), and SENP1 (415-643), NEDP1 (2-212) from the pHISTEV vector (N-terminal His-TEV). The SseL 24-340 construct may represent the full-length sequence secreted by *S.* Typhimurium, depending on usage of an alternative GTG start codon at position 24. The SseL VHS domain construct, cloned into pOPIN-B, included residues 24-137 as well as an 'EQVGVENLWRD' C-terminal sequence designed using the crystal structure to reconstitute key stabilizing interactions made by the C-terminal catalytic domain. With the exception of YopJ, SENP1, NEDP1, and SseL VHS domain, all tags were cleaved as part of the purification.
All constructs were transformed into the *E. coli* Rosetta2 pLacI strain. 2-12 L cultures were grown at 37˚C in 2xTY medium to an OD_{600} of 0.8-1.0, at which time protein expression was induced with 0.2 mM IPTG for 16-20 h at 18˚C. Cells were resuspended in 25 mM Tris (pH 7.4), 200 mM NaCl, 2 mM ß-mercaptoethanol (Buffer A). Following freeze-thaw and addition of EDTA-free Complete protease inhibitor tablets (Roche), DNase, and Lysozyme, cells were lysed by sonication or using an Emulsiflex C3. Lysates were clarified by centrifugation at 35000 x g for 25 min, and applied to Talon (Clontech) or Glutathione Sepharose 4B (GE Healthcare) resin for gravity flow purification according to manufacturer recommendations. His-tagged proteins were washed thoroughly with Buffer A, eluted in Buffer A with 250 mM imidazole, and (with the exception of SENP1 and NEDP1) the tag was cleaved overnight with His-3C protease during dialysis back to Buffer A. Cleaved protein was reapplied to Talon resin and flow-through was collected for further purification. GST-tagged proteins were washed thoroughly with Buffer B (25 mM Tris (pH 8.5), 5 mM DTT)) plus 500 mM NaCl, then Buffer B plus 50 mM NaCl, prior to elution with 10 mM GSH (in the case of YopJ) or overnight cleavage with addition of GST-3C protease. When required, cleaved proteins were exchanged into Buffer B plus 50 mM NaCl and applied to anion exchange chromatography (ResourceQ 6 mL, GE Healthcare), followed by elution with a 50-500 mM NaCl gradient. The SseL VHS domain was purified from inclusion bodies. Following solubilization in Buffer A plus 8 M Urea, 10 mM NH_4Cl, SseL VHS domain was bound to gravity flow Ni^{2+} resin (Qiagen), refolded using a gradient to Buffer A without Urea, and eluted in Buffer A plus 250 mM imidazole. All proteins were subjected to final gel filtration (Superdex75, GE Healthcare) equilibrated in 25 mM HEPES (pH 8.0), 150 mM NaCl, 5 mM DTT. Protein-containing fractions were concentrated, aliquoted, and flash-frozen prior to storage at -80˚C.

TAMRA-based cleavage assays

All Ub/Ubl-TAMRA-KG reagents were prepared as previously described (Geurink et al., 2012; Basters et al., 2014), with the exception of the tSUMO-TAMRA-KG reagent, which was prepared as follows:

Native chemical ligation

tSUMO-MesNa (aa 1-96, 4.0 mg, 0.37 μmol), prepared as previously described (Borodovsky et al., 2002), and TMR-Lys(thio)G (4.0 mg, 5.4 μmol) were dissolved in 200 μL of aqueous buffer containing 6.0 M Gn·HCl, 0.15 M Na_2HPO_4 and 0.25 M MPAA at pH 7.2 and shaken overnight at 37°C. The product was purified by RP-HPLC (Shimadzu, C18, 10-70% v/v ACN/H_2O gradient, 0.05% v/v TFA). The appropriate fractions were pooled and lyophilized.

Desulfurization

The resulting product (ca. 4 mg) was dissolved in aqueous buffer containing 6.0 M Gn·HCl, 0.15 M Na_2HPO_4 and 0.25 M TCEP at pH 7.0 to a concentration of 1 mg/mL protein. Reduced glutathione (GSH) was added to the solution to a concentration of 100 mM. The pH of the solution was adjusted to pH 7.0 by addition of 1 M NaOH. VA-044 was added to the solution to a final concentration of 75 mM. The reaction mixture was flushed with argon and shaken for 4 h at 37°C after which LC-MS analysis indicated a complete reaction. The product

was purified by RP-HPLC (Shimadzu, C18, 10-70% v/v ACN/H_2O gradient, 0.05% v/v TFA). The appropriate fractions were combined, lyophilized, dissolved in H_2O/ACN/formic acid (65/35/10; v/v/v; 10 mL) and lyophilized again. Finally, the product was purified on a Superdex75 size exclusion column (GE Healthcare) equilibrated in 50 mM Tris (pH 7.6), 100 mM NaCl. This yielded 0.85 mg (75 nmol, 21%) of the tSUMO-TAMRA-KG substrate.

Fluorescence polarization (FP) assays monitoring cleavage of the Ub/Ubl substrates were performed as described previously (Geurink et al., 2012). Briefly, enzymes and Ub/Ubl substrates were diluted in 25 mM Tris (pH 7.4), 100 mM NaCl, 5 mM ß-mercaptoethanol, 0.1 mg/mL BSA (FP buffer). Enzyme concentrations were varied to capture a representative activity profile, up to a maximum concentration of 150 nM. Substrate concentrations were held at 150 nM. Enzyme and substrate were mixed in black 384-well plates (Corning) immediately before the assay began. FP was measured in a Pherastar plate reader (BMG Labtech) equipped for 550 nm excitation and 590 nm emission. Cleavage measurements were normalized to independently measured negative (Ub/Ubl substrate only) and positive (25 nM TAMRA-KG peptide) control samples. All comparative data (Ub vs. Ubl, wild-type vs. mutant) are presented from one representative replicate.

Qualitative cleavage assays
Deubiquitination specificity assays were performed as described previously (Licchesi et al., 2012). Enzymes were diluted to a 2x stock concentration in 25 mM Tris (pH 7.4), 150 mM NaCl, 10 mM DTT and combined 1:1 with 6 µM diUb prepared in 100 mM Tris (pH 7.4), 100 mM NaCl, 10 mM DTT. Reactions were incubated at 37˚C, time points were resolved by SDS-PAGE and silver stained (BioRad).
Pro-NEDD8 (Boston Biochem) and Cul1-NEDD8 (kind gift from B. Schulman, St. Jude Children's Research Hospital) cleavage assays were performed with the same protocol.

Acetylation assays
Enzymes were diluted to 5 µM in 25 mM HEPES (pH 8.0), 50 mM NaCl, 200 nM inositol hexakisphosphate (IP6), 0.5 mM DTT and incubated with 60 µM [1-^{14}C] AcCoA (60 mCi/mmol, PerkinElmer) at 37˚C for 2 h. Reactions were resolved by SDS-PAGE. The gel was dried and exposed to a Phosphor screen, which was then imaged on a Typhoon scanner (GE Healthcare). In our assays, catalytically inactive mutants, as well as unrelated protein standards (not shown), can be non-enzymatically modified by AcCoA. Therefore, only an increased signal in wild-type assays compared to the catalytically inactive controls should be considered true acetylation activity.

Suicide probe assays
Ub- and Ubl-PA probes were generated as described previously (Ekkebus et al., 2013), with exception of tSUMO-PA and all Ub/tSUMO-PA point mutants, which were prepared according to (Borodovsky et al., 2002) with propargylamine. Assays testing Ub/NEDD8 specificity were performed using suicide probes prepared from 2-chloroethylamine, as described previously (Borodovsky et al., 2002). Enzymes were diluted to 10 µM in 25 mM Tris (pH 7.4), 150 mM NaCl, 10 mM DTT, and combined 1:1 with 100 µM probe. Reactions were incubated at room temperature or on ice (as specified), time points were resolved by SDS-PAGE and Coomassie stained.

Protein crystallization
Initial hits were obtained using commercial screens in the sitting-drop vapor diffusion format.
Native and SeMet His-SseL (24-340) were prepared in 25 mM Tris (pH 7.4), 200 mM NaCl, 2 mM ß-mercaptoethanol and crystallized at 14 mg/mL in 0.1 M Tris, 1.6 M K_2HPO_4 (final pH 8.7), with a 1 µL sitting drop at 2:1 protein:precipitant ratio. SseL crystals were cryoprotected in ParatoneN (native crystals) or paraffin oil (SeMet crystals).
Native ChlaDUB1 (130-401) was prepared in 25 mM Tris (pH 7.4), 125 mM NaCl, 4 mM DTT and crystallized at 12 mg/mL in 0.1 M HEPES (pH 7.5), 20% PEG 8000, with a 400 nL sitting drop at 1:1 protein:precipitant ratio. SeMet ChlaDUB1 was crystallized at 8 mg/mL in a 1 µL sitting drop of the matched condition, using seeds of native crystals. Crystals were cryoprotected using mother liquor containing 22-25% glycerol.
Native RickCE (420-691) was prepared in 25 mM Tris (pH 7.4), 125 mM NaCl, 4 mM DTT and crystallized by hanging drop, mixing 2 µL of 13 mg/mL protein with 1 µL of 0.1 M Tris (pH 7.5), 2 M ammonium citrate. SeMet RickCE was crystallized the same manner, using native RickCE crystals as seeds. Crystals either had no cryoprotectant (SeMet RickCE), or were cryoprotected with mother liquor containing 22-25% glycerol.
The XopD~Ub complex was prepared in 25 mM Tris (pH 7.4), 125 mM NaCl, 4 mM DTT and crystallized at 12 mg/mL in 0.1 M CHES (pH 9.5), 1.0 M sodium citrate, with a 200 nL sitting drop at 1:1 protein:precipitant ratio. Crystals were cryocooled with no protectant. The XopD~tSUMO complex was prepared similarly, and

crystallized at 8 mg/mL in 0.1 M bicine (pH 9.0), 1.6 M ammonium sulfate. XopD~tSUMO crystals were cryoprotected in 3.4 M malonate.

Data collection, structure determination, and refinement

Diffraction data were collected at the European Synchrotron Radiation Facility (ESRF) beam line ID23-1, and the Diamond Light Source (DLS) beam lines I03, I04, and I04-1 (see **Table 1**). Images were integrated using either MOSFLM (Battye et al., 2011) or XDS (Kabsch, 2010) and scaled using Aimless (Evans and Murshudov, 2013). Structures were solved experimentally using SeMet SAD datasets in PHENIX AutoSol and AutoBuild (Adams et al., 2010; Terwilliger et al., 2009; Terwilliger et al., 2008) (**Table 1**). XopD~Ub and XopD~tSUMO were solved using molecular replacement in Phaser (McCoy et al., 2007) using the apo XopD structure (PDB 2OIV) and Ub (PDB 1UBQ) or SUMO1 structures (PDB 1TGZ). Model building and refinement were performed using COOT (Emsley et al., 2010) and PHENIX (Adams et al., 2010). The XopD~Ub structure was refined with the higher resolution XopD structure (XopD~tSUMO) and Ub (PDB 1UBQ) as reference models. See **Table 1** for final statistics.

All figures were generated using PyMOL (www.pymol.org). Secondary structure topology diagrams are based on output from the Pro-origami server (Stivala et al., 2011).

Plant extract protease assays

S. lycopersicum leaves (kind gift from A. Canto-Pastor and D. Baulcombe, University of Cambridge) were flash frozen in liquid nitrogen and ground by mortar and pestle in a lysis buffer containing 50 mM HEPES (pH 8.0), 150 mM NaCl, 25 mM sucrose, 5 mM DTT, 1 mM EDTA, and 1 mM PMSF. Insoluble material was removed following 16000 x g centrifugation at 4˚C for 10 min. The clarified lysate was incubated with recombinant XopD constructs at a 1 µM final concentration for 1 h at room temperature. Thirty micrograms of the resulting samples were separated by SDS-PAGE, transferred to nitrocellulose, and blotted for total ubiquitin (Ubi-1, Novus Biologicals) following Ponceau staining of total protein.

Quantitative cleavage assays

FlAsH-tagged diUb substrates were assembled enzymatically (UBE2N/UBE2V1 for K63 chains, UBE2R1 for K48 chains) by ligating a C-terminally FlAsH tagged Ub to 'blocked' Ub mutants (K63R for K63 chains, K48R for K48 chains). Unlabeled diUb chains were assembled analogously, replacing FlAsH-tagged Ub with a construct lacking its C-terminus (Ub aa 1-72). FlAsH-labeled diUb was held at a constant concentration of 75nM in all assays, and the remaining substrate consisted of unlabeled diUb. Assays were performed in 20 µL using black 384-well plates (Corning). FP was measured in a Pherastar plate reader (BMG Labtech) equipped for 485 nm excitation and 520 nm emission. SseL wild-type was used at 100 nM and 10 µM final concentration for K63 and K48 measurements, respectively. SseL Y244A was used at 1 µM final concentration in all assays. For each substrate concentration, initial rates, measured in triplicate, were subtracted from a substrate-only control and converted into velocities of substrate conversion. Kinetic analysis was performed using Graphpad Prism 6.

Construction of CE dendrogram

Given the significant sequence and structural diversities within the CE clan, we adopted a multipronged approach to build an accurate sequence alignment as described below. 1) Bonafide members of the CE superfamily of proteases were obtained from MEROPS, Pfam, and Interpro databases (Rawlings et al., 2014; Finn et al., 2014; Mitchell et al., 2015). 2) A "seed" structure-based sequence alignment was constructed based on available structures of CE superfamily domains such as SENP1, SENP2, NEDP1, L3 23K of human adenovirus 2 and XopD (PDB ID: 2XPH, 3ZO5, 2BKR, 1AVP, and 2OIX, respectively) (Rimsa et al., 2011; Alegre and Reverter, 2014; Shen et al., 2005; Ding et al., 1996; Chosed et al., 2007) and the presented structures of SseL, ChlaDUB1, and RickCE. The alignment was constructed using MUSTANG (Konagurthu et al., 2006) and DaliLite (Holm and Park, 2000) programs and was further manually refined based on residue conservation patterns and assignment of secondary structures from the above PDB structures. 3) The alignment was then expanded to include sequence data from other families within the CE clan such as YopJ and the Poxviridae proteases. We also included more sequence data from the families already represented in the seed alignment. An initial version of the expanded alignment was constructed using MUSCLE program (Edgar, 2004) with the option of profile-to-sequence alignment, which does not perturb the original seed alignment. This expanded alignment was further manually refined based on residue conservation patterns and secondary structure prediction by JPRED (Drozdetskiy et al., 2015) to obtain a final expanded alignment. 4) Once an accurate version of the expanded alignment was constructed, we performed database searches using HMMER, PSI-BLAST and HHpred (Finn et al., 2011; Altschul et al., 1997; Söding et al., 2005) to find more divergent homologs of known CE superfamily members. This process identified e.g. the SidE family as part of the CE clan. Representative members of SidE family were then included in the expanded alignment. 5) MEGA software (Tamura et al., 2007) was used to construct a dendrogram based on the expanded alignment of the CE clan using

the UPGMA method with a bootstrap procedure for 1000 trials. A maximum likelihood method was also employed, but did not reliably produce dendrograms with cohesive functional relationships, likely due to high levels of dissimilarity among branchpoints.

GST pull-down assays
GST-tagged CI-MPR peptide was prepared by ligating sequence for the 'FHDDSDEDLLHI' peptide (CI-MPR aa 2480-2491) into the pOPIN-K vector, which encodes an N-terminal GST-3C tag. The wild-type sequence, as well as the 'LL-AA' double mutant were expressed and purified alongside the empty pOPIN-K vector, which contains the 'GTVDPTGKRAVSATQLM' sequence at its C-terminus as a control. 50 µg of GST or GST-MPR were phosphorylated by incubation with 250 units of CK2, 5 mM $MgCl_2$, and 1 mM ATP at 30˚C for 30 min. For the pull-down assay, 50 µg of each GST-tagged protein was bound to 25 µL of Glutathione Sepharose 4B resin equilibrated in 20 mM Tris (pH 7.4), 100 mM NaCl, 5 mM ß-mercaptoethanol, and washed 3x with 250 µL additional cold buffer. 50 µg of SseL was added to a final concentration of 25 µM, and allowed to bind on ice for 30 min. Samples were washed 7x with 250 µL of cold buffer, and eluted with 25 µL buffer containing 10 mM GSH. Samples of SseL loading and GST elutions were resolved by SDS-PAGE and visualized by Coomassie staining.

NMR spectroscopy
Uniformly labeled ^{15}N-Ub was expressed in M9 minimal media supplemented with ^{15}N-NH_4Cl, and purified as described previously (Pickart and Raasi, 2005). All proteins were exchanged into 25 mM sodium phosphate (pH 7.0), 150 mM NaCl, 1 mM DTT. NMR spectra were recorded at 298K on either a Bruker Avance III 600 MHz or Avance2+ 700 MHz spectrometer, equipped with cryogenic triple resonance TCI probes. Data processing and analysis were performed in Topspin (Bruker) and NMRView (One Moon Scientific).

S. Typhimurium infection assays
The *S.* Typhimurium Δ*sseL* strain was transformed with pWSK29 plasmids containing SseL-2HA mutants (see table of strains below). SPI-2 dependent secretion of SseL was tested using the pH shift secretion assay (Yu et al., 2010). Briefly, overnight cultures were diluted 1:50 into Mg-MES (pH 5) and grown for 4 hours, then transferred to Mg-MES (pH 7.2) for 90 minutes. Secreted protein and bacterial cells were collected and resolved by SDS-PAGE and immunoblotted with DnaK (*Salmonella* intracellular chaperone, Enzo ADI-SPA-880-D), SseB (SPI-2 T3SS translocon protein, generated in Beuzón et al., 2002) and HA (SseL, HA.11 Cambridge Biosciences) antibodies.

HeLa cells were infected for 16 hours at a multiplicity of infection (MOI) of 200 with late exponential phase *S.* Typhimurium strains. Cells were collected in SDS sample buffer for total cell lysate or collected into Phosphate-Buffered Saline (PBS), pelleted at 300 x g and lysed in PBS + 0.1% triton. The lysate was clarified at 3,000 x g and the supernatant collected as the cytosolic fraction. Samples were resolved by SDS-PAGE and immunoblotted with HA.11 (SseL), DnaK (*S.* Typhimurium intracellular chaperone), or Actin antibodies. HeLa cells seeded on coverslips were infected as above and fixed with 3% Paraformaldehyde at 16 hpi. 0.1% saponin was used to permeabilize HeLa cells membrane but not bacterial membranes. Translocated SseL and *S.* Typhimurium were detected using HA (3F10 Roche) and CSA-1 (Insight Biotechnology) antibodies, respectively.

Strains used in this study:

Strain	Description	Source
ΔsseL	ΔsseL:Km in 12023	(Rytkönen et al., 2007)
ΔsseL pSseL 2HA	pWSK29*sseL*-2HA in ΔsseL	(Rytkönen et al., 2007)
ΔssaV pSseL 2HA	pWSK29*sseL*-2HA in ΔssaV	(Rytkönen et al., 2007)
ΔsseL pSseL 2HA Y183A	pWSK29*sseLY183A* -2HA in ΔsseL	This study
ΔsseL pSseL 2HA W105A	pWSK29*sseLW105A*-2HA in ΔsseL	This study

Supplemental References:

Adams, P.D., Afonine, P.V., Bunkoczi, G., Chen, V.B., Davis, I.W., Echols, N., Headd, J.J., Hung, L.W., Kapral, G.J., Grosse-Kunstleve, R.W., McCoy, A.J., Moriarty, N.W., Oeffner, R., Read, R.J., Richardson, D.C., Richardson, J.S., Terwilliger, T.C., and Zwart, P.H. (2010). PHENIX: a comprehensive Python-based system for macromolecular structure solution. Acta Crytallogr. D Biol. Crystallogr. *66*, 213-221.

Altschul, S.F., Madden, T.L., Schäffer, A.A., Zhang, J., Zhang, Z., Miller, W., and Lipman, D.J. (1997). Gapped BLAST and PSI-BLAST: a new generation of protein database search programs. Nucl. Acids Res. *25*, 3389-3402.

Battye, T.G.G., Kontogiannis, L., Johnson, O., Powel, H.R., and Leslie, A.G.W. (2011). iMOSFLM: a new graphical interface for diffraction-image processing with MOSFLM. Acta Crytallogr. D Biol. Crystallogr. *67*, 271-281.

Berrow, N.S., Alderton, D., Sainsbury, S., Nettleship, J., Assenberg, R., Rahman, N., Stuart, D.I., and Owens, R.J. (2007). A versatile ligation-independent cloning method suitable for high-throughput expression screening applications. Nucl. Acids Res. *35*, e45.

Beuzón, C.R., Banks, G., Deiwick, J., Hensel, M., and Holden, D.W. (2002). pH-dependent secretion of SseB, a product of the SPI-2 type III secretion system of *Salmonella typhimurium*. Mol. Microbiol. *33*, 806-816.

Borodovsky, A., Ovaa, H., Kolli, N., Gan-Erdene, T., Wilkinson, K.D., Ploegh, H.L., and Kessler, B.M. (2002). Chemistry-based functional proteomics reveals novel members of the deubiquitinating enzyme family. Chem. Biol. *9*, 1149-1159.

Drozdetskiy, A., Cole, C., Procter, J., and Barton, G.J. (2015). JPred4: a protein secondary structure prediction server. Nucl. Acids Res. *43*, W389-W394.

Edgar, R.C. (2004). MUSCLE: multiple sequence alignment with high accuracy and high throughput. Nucl. Acids Res. *32*, 1792-1797.

Emsley, P., Lohkamp, B., Scott, W.G., and Cowtan, K. (2010). Features and development of Coot. Acta Crytallogr. D Biol. Crystallogr. *66*, 486-501.

Evans, P.R. and Murshudov, G.N. (2013). How good are my data and what is the resolution? Acta Crytallogr. D Biol. Crystallogr. *69*, 1204-1214.

Finn, R.D., Clements, J., and Eddy, S.R. (2011). HMMER web server: interactive sequence similarity searching. Nucl. Acids Res. *39*, W29-W37.

Finn, R.D., Bateman, A., Clements, J., Coggill, P., Eberhardt, R.Y., Eddy, S.R., Heger, A., Hetherington, K., Holm, L., Mistry, J., Sonnhammer, E.L.L., Tate, J., and Punta, M. (2014). The Pfam protein families database. Nucl. Acids Res. *42*, D222-D230.

Holm, L. and Park, J. (2000). DaliLite workbench for protein structure comparison. Bioinformatics. *6*, 566-567.

Kabsch, W. (2010). XDS. Acta Crytallogr. D Biol. Crystallogr. *66*, 125-132.

Konagurthu, A.S., Whisstock, J.C., Stuckey, P.J., and Lesk, A.M. (2006). MUSTANG: a multiple structural alignment algorithm. Proteins. *64*, 559-574.

Licchesi, J.D.F., Mieszczanek, J., Mevissen, T.E.T., Rutherford, T.J., Akutsu, M., Virdee, S., El Oualid, F., Chin, J.W., Ovaa, H., Bienz, M., and Komander, D. (2012). An ankyrin-repeat ubiquitin-binding domain determines TRABID's specificity for atypical ubiquitin chains. Nat. Struct. Mol. Biol. *19*, 62-71.

McCoy, A.J., Grosse-Kunstleve, R.W., Adams, P.D., Winn, M.D., Storoni, L.C., and Read, R.J. (2007). Phaser crystallographic software. J. Appl. Cryst. *40*, 658-674.

Mitchell, A., et al. (2015). The InterPro protein families database: the classification resource after 15 years. Nucl. Acids Res. *43*, D213-D221.

Pickart, C.M. and Raasi, S. (2005). Controlled synthesis of polyubiquitin chains. Method. Enzymol. *399*, 21-36.

Rawlings, N.D., Waller, M., Barrett, A.J., and Bateman, A. (2014). MEROPS: the database of proteolytic enzymes, their substrates and inhibitors. Nucl. Acids Res. *42*, D503-D509.

Rimsa, V., Eadsforth, T., and Hunter, W.N. (2011). The role of Co^{2+} in the crystallization of human SENP1 and comments on the limitations of automated refinement protocols. Acta Crytallogr. F Struct. Biol. Cryst. Commun. *67*, 442-445.

Söding, J., Biegert, A., and Lupas, A.N. (2005). The HHpred interactive server for protein homology detection and structure prediction. Nucl. Acids Res. *33*, W244-W248.

Stivala, A., Wybrow, M., Wirth, A., Whisstock, J., and Stuckey, P. (2011). Automatic generation of protein structure cartoons with Pro-origami. Bioinformatics. *27*, 3315-3316.

Tamura, K., Dudley, J., Nei, M., and Kumar, S. (2007). MEGA4: Molecular Evolutionary Genetics Analysis (MEGA) software version 4.0. Mol. Biol. Evol. *24*, 1596-1599.

Terwilliger, T.C., Adams, P.D., Read, R.J., McCoy, A.J., Moriarty, N.W., Grosse-Kunstleve, R.W., Afonine, P.V., Zwart, P.H., and Hung, L.W. (2009). Decision-making in structure solution using Bayesian estimates of map quality: the PHENIX AutoSol wizard. Acta Crytallogr. D Biol. Crystallogr. *65*, 582-601.

Terwilliger, T.C., Grosse-Kunstleve, R.W., Afonine, P.V., Moriarty, N.W., Zwart, P.H., Hung, L.W., Read, R.J., and Adams, P.D. (2008). Iterative model building, structure refinement and density modification with the PHENIX AutoBuild wizard. Acta Crytallogr. D Biol. Crystallogr. *64*, 61-69.

Yu, X.J., McGourty, K., Liu, M., Unsworth, K.E., and Holden, D.W. (2010). pH sensing by intracellular *Salmonella* induces effector translocation. Science. *328*, 1040-1043.
